# Eco-Friendly Functionalization of Ynals with Thiols under Mild Conditions

**DOI:** 10.3390/ijms25179201

**Published:** 2024-08-24

**Authors:** Kamil Hanek, Patrycja Żak

**Affiliations:** Faculty of Chemistry, Adam Mickiewicz University in Poznań, Uniwersytetu Poznańskiego St. 8, 61-614 Poznan, Poland; kamil.hanek@amu.edu.pl

**Keywords:** thioester, ynal, sulfa-Michael adduct, N-heterocyclic carbene, organocatalysis

## Abstract

A new eco-friendly method for the synthesis of mono- and multifunctional organosulfur compounds, based on the process between ynals and thiols, catalyzed by bulky *N*-heterocyclic carbene (NHC), was designed and optimized. The proposed organocatalytic approach allows the straightforward formation of a broad range of thioesters and sulfenyl-substituted aldehydes in yields above 86%, in mild and metal-free conditions. In this study, thirty-six sulfur-based derivatives were obtained and characterized by spectroscopic methods.

## 1. Introduction

The knowledge about organosulfur compounds (OSCs) is of great importance because of a large number of their applications in medicine [1,2,3], pharmaceutics [4,5,6,7,8], polymer [9], and food industries [10]. Thus, the exploration of their chemistry and the search for new classes of materials containing sulfur atoms are often discussed by scientists all over the world.

**Scheme 1:**
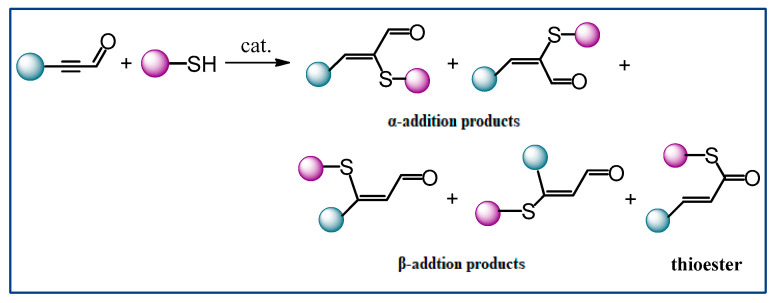
Main possible products of the functionalization of ynals with thiols.

Among all the organic sulfur derivatives, of particular interest are thioesters and sulfa-Michael addition (SMA) products, which can serve as building blocks in various processes [11,12,13,14]. They exhibit potential catalytic activity in cascade reactions [15], display antiproliferative properties [16], or act as SARS-CoV-2MPRO inhibitors [17]. Despite the great practical importance of materials of this type, most of the prominent methods for their synthesis present some serious drawbacks. For example, thioesters can be obtained by the nucleophilic substitution of the carbonyl group of acyl chlorides, carboxylic acids, or acid anhydrides with thiols [18] or reactions with aldehydes used as starting materials [19]. However, these reactions are possible to perform only with the use of transition metal complexes of copper and iron, oxidants, and at high temperatures [20,21,22]. Alternatively, thioesters can be obtained in the presence of transition metal complexes as catalysts in the thiocarbonylation of alkenes and alkynes, although, in most cases, these processes require harsh conditions, like high temperatures and/or elevated pressures [23,24]. SMA reactions remain less explored, mainly because of the high nucleophilicity of thiols causing difficulties in controlling the stereoselectivity [25]. They are generally performed at low temperatures, but unfortunately, the reactions require the use of a catalyst at high concentrations [26,27,28,29]. Such conditions are not considered ecologically friendly, hence organocatalytic reactions based on non-toxic NHCs are rising to prominence because of their successful application in the synthesis of both thioesters [30] and sulfenyl-substituted aldehydes [31,32,33,34,35]. The experiments also conducted in our group have proved that NHCs are powerful tools in the synthesis of organosulfur compounds from unsaturated scaffoldings with electron-withdrawing moieties. The research performed with enals led to obtaining new groups of sulfur compounds, e.g., silsesquioxane derivatives [36] and symmetric and unsymmetric bis(thioesters) [37]. Inspired by our previous work and bearing in mind the continuous demand for organosulfur moieties as well as the limited number of eco-friendly procedures for their synthesis, we turned our attention to ynals, which can be used as versatile synthons for the synthesis of various heterocycles (e.g., indolizines) [38]. They are the substrates that are more demanding for functionalization than α,β-unsaturated aldehydes because they comprise two reactive groups suitable for modification. Thus, in the reaction between ynals and mercaptans, besides regio- and stereoselectivity, chemoselectivity must also be controlled (Scheme 1).

To the best of our knowledge, there are no literature reports on the modifications of ynals with thiols. It is thus of great importance to design new catalytic systems that would permit the fully selective formation of expected products for a wide range of starting mercaptans and aldehydes containing C≡C bonds, as it would reveal a new strategy to obtain structurally diverse organosulfur compounds, including chiral alcohols.

Herein, we describe an effective synthetic pathway for obtaining a new class of organosulfur materials via reactions of ynals with thiols and dithiol in the presence of bulky NHC carbene. We focused our attention on designing an eco-friendly system enabling the introduction of one or more, the same or different, sulfur groups into the aldehyde structure. Finally, we synthesized, isolated, and characterized thirty-six carbonyl compounds not yet described in the literature.

## 2. Results and Discussion

### 2.1. Design and Optimization of the Reaction System

In the first stage of the study, a number of tests was carried out in order to find the optimal conditions of 1-octynal’s (**1a**) functionalization. Benzyl mercaptan (**2a**) was used as the reaction partner, because of its low price and high commercial availability. The tests were performed in the presence of bulky NHC salt–IPr*^OMe^·HCl (**NHC-1**) and their course was monitored by GC-MS analyses. The results presented in Table 1 reveal that the outcomes of the hydrothiolation process depends largely on the reaction media and temperatures. Among all the solvents tested, acetone was found to be the best one in terms of both activity and selectivity to the **P1** product (Table 1, Entries 8–11 and 15), which is especially important from an ecological perspective as acetone is classified as a green solvent [39]. An effective transformation of reagents was also observed when the process was carried out in MTBE or toluene, but then a mixture of two products was obtained (Table 1, Entries 4 and 7). The other solvents (DCE, DCM, and n-hexane) turned out to be ineffective in the tested model reaction (Table 1, Entries 1–3). Moreover, it was observed that the reaction efficiency depended also on the amount of solvent used. When the process was performed in solvent-free conditions, 40% of thioesterification product (**P1’**) was detected (Table 1, Entry 16). The same problem was observed for the tests conducted at temperatures above RT (Table 1, Entries 6–9). Catalytic screening demonstrated that catalyst loading affects the efficiency of the process, because a lowering of its concentration to 2.5 mol% leads to a reduction in the yield, despite a significant extension of the reaction time (Table 1, Entry 11). Carrying out the reaction without the additions of **NHC-1** (Table 1, Entry 13) or potassium hexamethyldisilazane (KHMDS) (Table 1, Entry 14) is impossible, which confirms that the catalyst and the base are indispensable for effective reactions. It was also found that the atmosphere in which the process was carried out had a significant impact on the efficiency of the reaction tested. This process must be performed under dry argon conditions. Otherwise, the main product of the reaction practically does not form (Table 1, Entry 12). We did not observe significant differences between the process carried out in the presence of carbene generated in situ and the reaction catalyzed by freshly isolated free carbene (Table 1, Entry 10 vs. Entry 15). Finally, the conditions of entry 10 were optimal as they allowed a quantitative conversion using relatively low catalyst loading.

### 2.2. Scope of the Reaction

**Figure 1:**
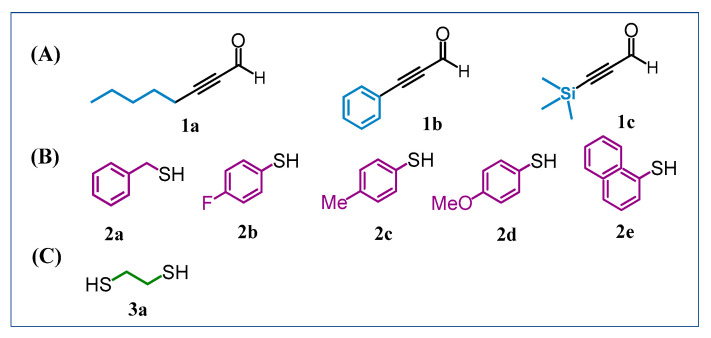
Substrates tested: (**A**) ynals, (**B**) thiols, and (**C**) dithiol.

The positive outcome obtained for the model reaction has encouraged us to determine the scope of the reaction. In the first series of experiments, we tested the reactivity of commercially available alkyl (**1a**), aryl (**1b**), and silyl (**1c**) propargyl aldehydes toward selected benzyl (**2a**) and aryl (**2b**–**e**) thiols as well as dithiol (**3a**) (Figure 1).

**Scheme 2:**
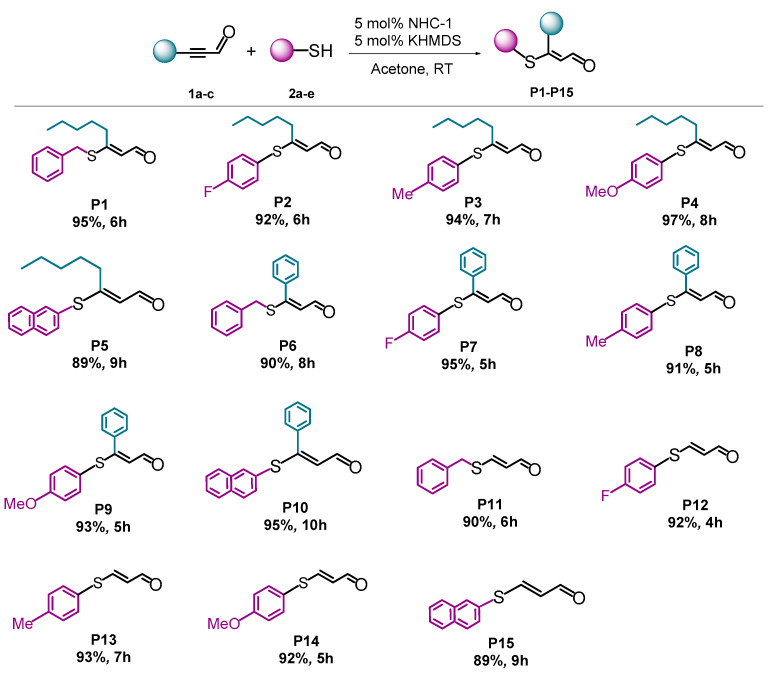
Functionalization of propargyl aldehydes (**1a**–**c**) with thiols (**2a**–**e**) (experimental conditions: acetone, RT, (**1a**–**c**):(**2a**–**e**) = 1:1, **NHC-1** = IPr*^OMe^·HCl, [**NHC-1**] = 5 mol%, and [KHMDS] = 5 mol%, argon). Isolated yields and reaction times are presented under the structures.

All results are presented in Scheme 2.

The proposed method can be successfully applied for the tested reagents, because all the products were obtained with excellent isolated yields (>89%) in very mild conditions and with full atom economy. When using 2-naphthalenethiol (**2e**), a slight increase in the reaction time was necessary to obtain the expected materials in quantitative yields. In each reaction, we observed the selective formation of the expected hydrothiolation product, whose geometry around the double bond was further confirmed by 2D NMR analyses (see ESI for details). Interestingly, the hydrothiolation of 3-(trimethylsilyl)-2-propynal (**1c**), irrespective of the thiol used, proceeded with the cleavage of the trimethylsilyl group (TMS). This result is particularly important in the context of the use of the obtained materials (**P11**–**P15**), which are convenient reagents for further modifications.

**Scheme 3:**
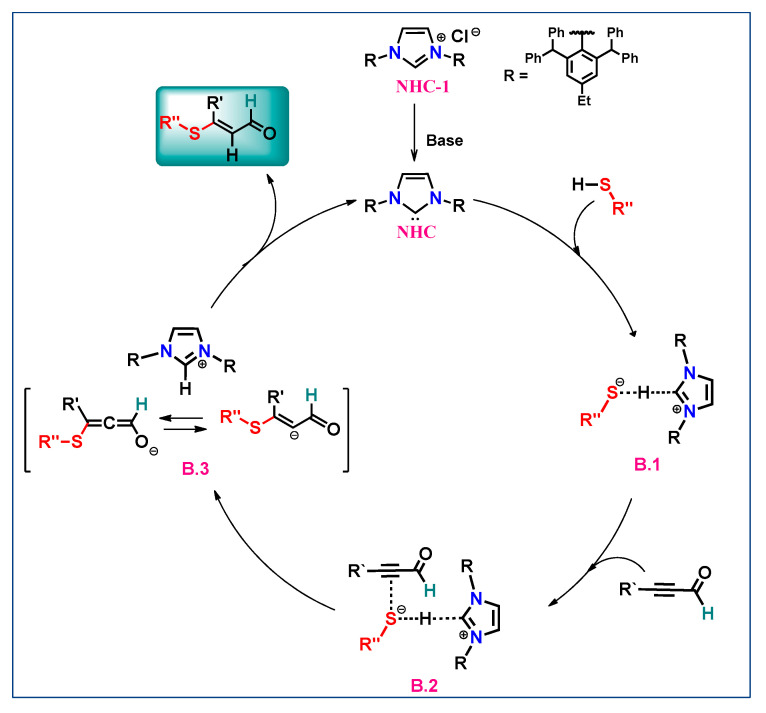
Proposed mechanism for the formation SMA products.

Based on the literature and our previous research concerning NHC-catalyzed thioester synthesis from α,β-unsaturated aldehydes with thiols [36], we proposed the mechanism depicted in Scheme 3.

Products **P1**–**P10** are formed as a result of NHC-catalyzed sulfa-Michael addition according to the proton transfer mechanism [31,40,41], which commences with the generation of free carbene (NHC) as a result of the reaction of its precursor (NHC-1) with KHMDS. NHC acts a Brønsted base, which deprotonates the acidic thiol molecule to form an NHC-thioxy intermediate (**B.1**). Because of the presence of the electron-withdrawing aldehyde group, the acetylenic moiety is activated and constitutes a perfect substrate for immediate conjugate addition [42] with the obtained NHC-thioxy intermediate [43] (**B.2**). The way in which ynal is coordinated strongly dependents on the steric properties of the organocatalyst. Because of the bulky NHC ligand, the thiol is pushed above the flat N-heterocycle upon the hydrogen bond formation between sulfur and hydrogen atoms. It permits the approach of the Michael acceptor from the top, with the steric group at the β carbon atom oriented away from the large aryl substituents attached to the NHC ligand [33,44]. Due to the presence of an oxygen atom, the formed intermediate can remain in equilibrium with allenic enolate [45] (**B.3**). Such a species is highly reactive and, immediately, *E*-products and regenerated free NHC are formed. As mentioned above, the use of aldehyde **1c** as a substrate led to unexpected results because, upon its modification, desilylation was observed. Unfortunately, at this moment, we are unable to clearly explain at what stage of the process the elimination of the TMS group occurs. However, we believe that theoretical research will help resolve this issue in the near future.

**Scheme 4:**
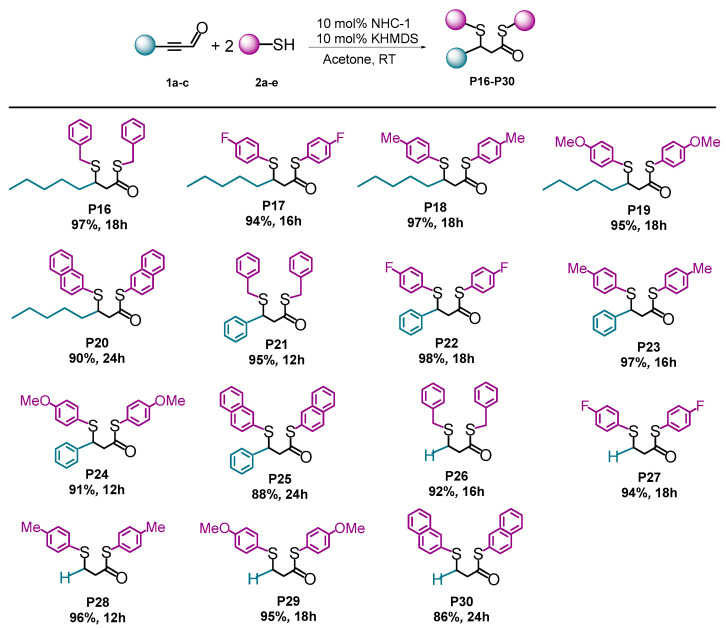
Bis-functionalization of propargyl aldehydes (**1a**–**c**) with thiols (**2a**–**e**) (experimental conditions: acetone, RT, (**1a**–**c**):(**2a**–**e**) = 1:2, **NHC-1** = IPr*^OMe^·HCl, [**NHC-1**] = 10 mol%, and [KHMDS] = 10 mol%, argon). Isolated yields and reaction times are presented under the structures.

Encouraged by the high activity of NHC-1 in the hydrothiolation of ynals (**1a**–**c**) with thiols (**2a**–**e**) used at an equimolar ratio, we examined the reactions with a two-fold excess of mercaptans. Thus, we treated 1 equiv. of aldehyde (**1a**–**c**) and 2 equiv. of thiol (**2a**–**e**) with NHC carbene generated from precursor NHC-1, and the reaction mixture was stirred at RT until the full conversion of ynal was detected by GC-MS analysis. All results are summarized in Scheme 4.

The performed tests revealed that, in the above conditions, the processes selectively produced bis-sulfenylated compounds in high isolated yields. No meaningful differences in the efficiency and selectivity of the process for the applied reagents were observed, except for 2-naphthalenethiol (**2e**). For this sterically crowded thiol, a longer time was indispensable to achieve a satisfactory result. Similarly, as for the reaction with the reagents at equimolar amounts, upon the functionalization of aldehyde **1c**, desilylation was observed.

**Figure 2:**
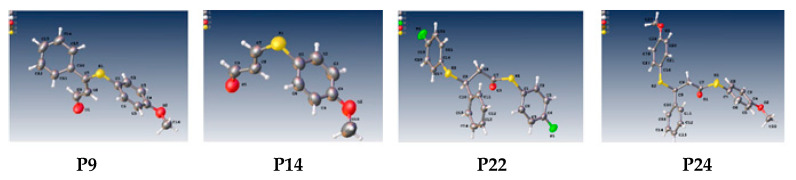
Perspective views of products **P9**, **P14**, **P22**, and **P24**.

All the products (**P1**–**P30**) were purified by column chromatography on silica gel using *n*-hexane or a 2:1 *v/v* mixture of n-hexane and DCM as eluents. Moreover, we were able to obtain crystals of the mono-sulfenylated products of 4-methoxythiophenol (**2d**) with phenylpropargyl aldehyde (**1b**) and 3-(trimethylsilyl)-2-propynal (**1c**) by dissolving **P9** and **P14** in *n*-hexane/DCM and the slow evaporation of the solvent. In a similar way, we obtained the crystals of two bis-sulfenylated products **P22** and **P24**. The molecular structures were determined by X-ray structural analyses and confirmed the formation of the expected materials (Figure 2) (see ESI for details).

**Scheme 5:**
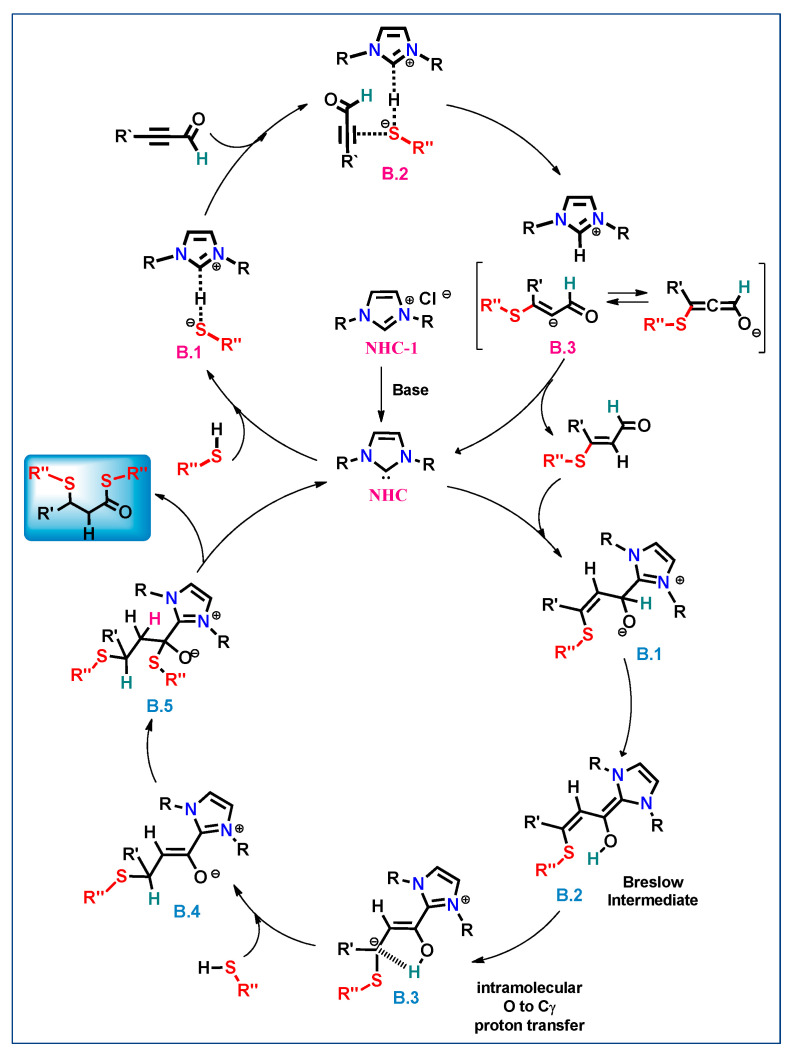
Proposed mechanism of bis-functionalized product formation.

Similarly to the assumed mechanism of monosubstituted product formation, we proposed a mechanism of bis-functionalized product formation based on the available literature (Scheme 5).

The first part of the mechanism runs in exactly the same way as that in Scheme 3, leading to the creation of a monosulfenylated product. Then, the thioesterification process occurs. The free carbene reacts with α,β-unsaturated aldehyde to produce intermediate **B.1**, which then undergoes proton transfer, producing a Breslow intermediate (**B.2**) [46,47,48]. The resulting intermediate remains in equilibrium with homoenolate (**B.3**) [49], which takes part in direct proton transfer from the hydroxyl group to the γ carbon [50,51]. Finally, the intermediate (**B.4**) reacts with thiol leading to catalyst regeneration.

**Scheme 6:**
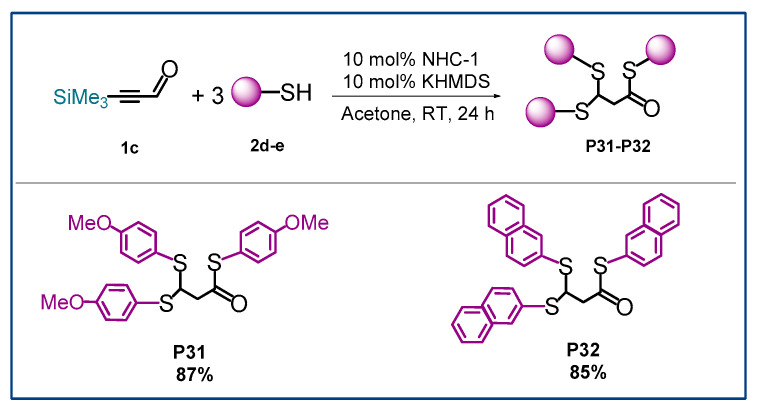
Triple functionalization of 1c with selected thiols (**2d**–**e**) (experimental conditions: acetone, RT, (**1c**):(**2d**–**e**) = 1:3, **NHC-1** = IPr*^OMe^·HCl, [**NHC-1**] = 10 mol%, and [KHMDS] = 10 mol%, argon). Isolated yields and reaction times are presented under the structure of each product.

In the next step, we checked the possibility of the formation of triple functionalized products. In the optimized reaction systems, the tests were performed with the use of a three-fold excess of thiols (**2d**–**e**) relative to 3-(trimethylsilyl)-2-propynal (**1c**). In both reactions, quantitative conversions of reagents were achieved within 24 h. The structures of products were determined by GC-MS and NMR analyses and are presented in Scheme 6.

**Scheme 7:**
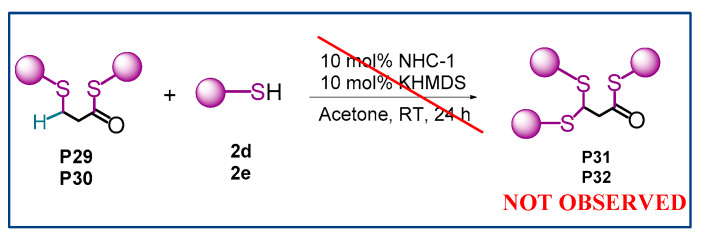
Functionalization of P29 and P30 with thiols (**2d, 2e**) (experimental conditions: acetone, RT, (P):(2) = 1:1, **NHC-1** = IPr*^OMe^·HCl, [**NHC-1**] = 10 mol%, and [KHMDS] = 10 mol%, argon).

The formation of (tris)sulfenylated products, although confirmed by spectroscopic NMR analyses, was rather unexpected, so we decided to run some additional experimental tests in order to obtain some insights into the reaction route (Scheme 7).

The results show that it is impossible to obtain such products by the gradual addition of 3 equiv. of thiol or as a result of the reaction of isolated (bis)sulfenylated product with 1 equiv. of thiol. Products **P31** and **P32** can be synthesized only by the implementation of a threefold excess of thiol, which leads to the conclusion that the concentration of thiol must have an impact on the reaction mechanism. In our opinion, in the case of a higher concentration of thiol in the reaction system, a monosulfenylated product is formed at first, followed by the addition of the second thiol equivalent to the newly formed carbon–carbon double bond, and finally, the thioesterification process occurs, according to the mechanism based on the Breslow intermediate, as described above.

**Scheme 8:**
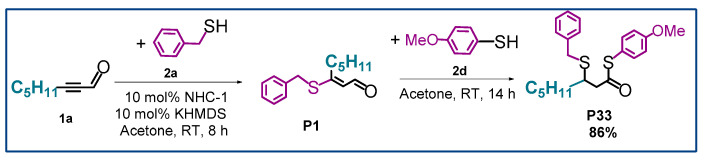
Functionalization of 1-octynal (**1a**) with two different thiols (**2a**, **2d**) (experimental conditions: acetone, RT, (**1a**):(**2a**):(**2d**) = 1:1:1, **NHC-1** = IPr*^OMe^·HCl, [**NHC-1**] = 10 mol%, and [KHMDS] = 10 mol%, argon). Isolated yield is presented under the structure of P33.

The successful coupling between unsaturated ynals (**1a**–**c**) with two- and three-fold excess amounts of thiols (**2a**–**e**) to yield multi-sulfenylated derivatives (**P16**–**P32**) prompted us to carry out two successive reactions with separate mercaptan products to check the feasibility of a one-pot protocol, leading to a novel class of unsymmetrically functionalized materials containing two sulfur atoms. Hence, we generated a free carbene in situ in the reaction environment, as a result of the deprotonation of a salt, NHC-1, and added equimolar amounts of **1a** and **2a**. The reaction mixture was stirred at RT until the full conversion of reagents was detected by GC-MS analysis. Then, the second type of thiol (**2d**) was added, and the catalytic system was left overnight. As presented in Scheme 8, we observed the selective formation of bis-functionalized product **P33** containing two different thiol moieties in high isolated yields.

**Scheme 9:**
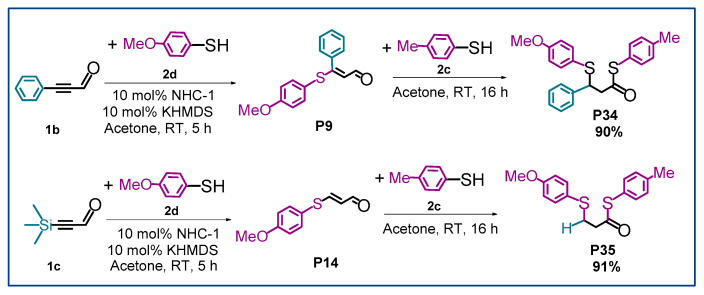
Functionalization of 1b and 1c with two different thiols (**2c**, **2d**) (experimental conditions: acetone, RT, (**1b-c**):(**2c**):(**2d**) = 1:1:1, **NHC-1** = IPr*^OMe^·HCl, [**NHC-1**] = 10 mol%, and [KHMDS] = 10 mol%, argon). Isolated yields are presented under the structures.

Analogously, we carried out the functionalization of **1b** and **1c** with **2c** and **2d** (Scheme 9). Highly gratifyingly, quantitative conversions were achieved within a reasonable period of time, irrespective of the nature of the aldehyde substituent. The hydrothiolation of 3-(trimethylsilyl)-2-propynal (**1c**) proceeded with the elimination of the trimethylsilyl group. Products **P34** and **P35** were obtained with the isolated yields of 90% and 91%, respectively, which confirm the universality of the proposed procedure.

**Scheme 10:**
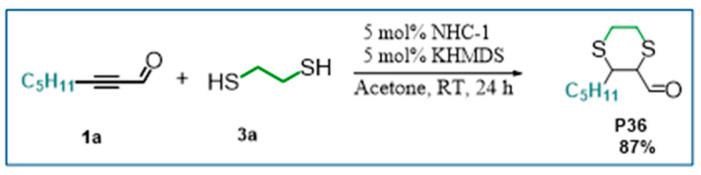
Functionalization of **1a** with 1,2-ethanedithiol (**3a**) (experimental conditions: acetone, RT, (**1a**):(**3a**) = 1:1, **NHC-1** = IPr*^OMe^·HCl, [**NHC-1**] = 5 mol%, and [KHMDS] = 5 mol%, argon). Isolated yield is presented under the structure of **P36**.

Finally, we turned our attention to the functionalization of ynals with dithiol. For this purpose, we performed the equimolar reaction between **1a** and 1,2-ethanedithiol (**3a**) in the presence of 5 mol% of **NHC-1** (Scheme 10). The reaction was highly selective, because we observed the exclusive formation of the cyclic product **P36**. The obtained compound can constitute a building block for the synthesis of, e.g., enamines, hemiacetals, or acetals.

### 2.3. The Preparative Scale for the Synthesis of Product ***P1***

**Scheme 11:**
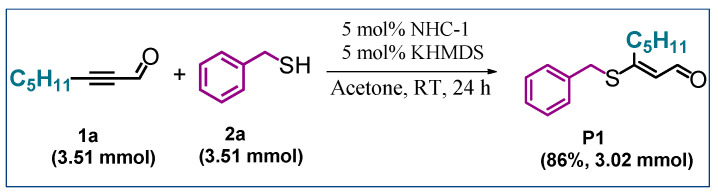
Scaled-up synthesis of **P1**.

To demonstrate the synthetic utility of the designed protocol, a gramscale hydrothiolation of **1a** with **2a** was performed (Scheme 11). The result obtained makes it clear that the proposed methodology has significant application potential.

In this study, thirty-six products were obtained, isolated, and characterized by spectroscopic methods (see ESI for details). These materials are air-stable, which makes them very attractive for further applications. The presented derivatives are new compounds that have never been published before.

## 3. Materials and Methods

### 3.1. General Methods and Chemicals

Unless otherwise indicated, all operations were carried out under a dry argon atmosphere, using standard Schlenk techniques. ^1^H NMR and ^13^C NMR spectra were recorded in CDCl_3_ on a Varian 400 operating at 402.6 and 101.2 MHz, respectively. HMBC and HSQC spectra were recorded on a Bruker Avance DRX 600 (Bruker, Billerica, MA, USA), operating at a frequency of 600.13 MHz (^1^H). Chemical shifts are reported in ppm with reference to the residual solvent peaks in ^1^H and ^13^C NMR. ^19^F NMR spectra were obtained using a Bruker Ascend^TM^ 400 NANOBAY, operating at 376 MHz. IR spectra were recorded on a Bruker IFS 66v/S spectrophotometer, and the scan range was 4000–400 cm^−1^. GC-MS analyses were performed on a Varian Saturn 2100T equipped with a DB-1 capillary column (30 m length, 0.25 mm internal diameter) and an ion trap detector. Thin-layer chromatography (TLC) was conducted on plates coated with a 250 μm thick silica gel layer and column chromatography was performed on silica gel 60 (70–230 mesh). ESI-MS spectra were obtained using a Synapt Gs-S HDMS (Waters, Milford, MA, USA) mass spectrometer with an electrospray ion source and quadrupole time-of-flight analyzer with resolving power (FWMH 38000). Acetonitrile was used as the sample solvent. The capillary voltage was set to 4.5 kV, sampling was set 40, and the source temperature was equal to 120 °C. The most abundant ions in the ESI-MS spectra were the sodiated and potassiated ions of the desired products.

All reagents, except the NHC carbene precursor, were purchased from commercial sources and used as received. NHC salt was prepared according to the literature procedure [52]. All the solvents, except THF, were dried over CaH_2_ prior to use and stored over 4 Å molecular sieves under argon. Dichloromethane was additionally passed through an alumina column and degassed by repeated freeze–pump–thaw cycles. THF was dried over sodium benzophenone ketyl and freshly distilled prior to use.

### 3.2. General Procedure for Catalytic Tests

#### 3.2.1. Mono- and Multifunctionalization of Aldehydes with (Di)thiols

An oven-dried 5 mL glass reactor equipped with a magnetic stirring bar was charged under argon with the NHC carbene precursor **NHC-1** (3.44 mg, 3.51 × 10^−6^ mol), KHMDS (0.70 mg, 3.51 × 10^−6^ mol), and acetone (1 mL). The reaction mixture was stirred at RT and, after 30 min, aldehyde **1a**–**c** (1 equiv., 7.01 × 10^−5^ mol), thiol **2a**–**e** (1 equiv., 7.01 × 10^−5^ mol, 2 equiv., 1.40 × 10^−4^ mol or 3 equiv., 2.10 × 10^−4^ mol), or dithiol **3a** (1 equiv., 7.01 × 10^−5^ mol) and an internal standard (decane or dodecane, 20 μL) were added under argon. The reaction mixture was heated at RT and its course was monitored by GC-MS.

#### 3.2.2. Bis-Functionalization of Aldehydes with Two Various Thiols

An oven-dried 5 mL glass reactor equipped with a magnetic stirring bar was charged under argon with the NHC carbene precursor **NHC-1** (3.44 mg, 3.51 × 10^−6^ mol), KHMDS (0.70 mg, 3.51 × 10^−6^ mol), and acetone (1 mL). The reaction mixture was stirred at RT and, after 30 min, aldehyde **1a**–**c** (1 equiv., 7.01 × 10^−5^ mol), thiol **2a**–**e** (1 equiv., 7.01 × 10^−5^ mol) and internal standard (decane or dodecane, 20 μL) were added under argon. The reaction mixture was heated at RT and, after 10 h, second thiol **2a**–**e** (1 equiv., 7.01 × 10^−5^ mol) was added under argon. The reaction mixture was heated at RT and its course was monitored by GC-MS.

### 3.3. General Procedure for the Synthesis of Products ***P1***–***P32*** and ***P36***

A 10 mL high-pressure Schlenk vessel equipped with a magnetic stirring bar and connected to the gas and vacuum line was charged with the NHC carbene precursor **NHC-1** (17.19 mg, 1.75 × 10^−5^ mol—5 mol% or 34.38 mg, and 3.5 × 10^−5^ mol—10 mol%), KHMDS (3.50 mg, 1.75 × 10^−5^ mol—5 mol% or 7.00 mg, and 3.5 × 10^−5^ mol—10 mol%), and acetone (2 mL) under argon. After 30 min of vigorous stirring the solution at RT, aldehyde **1a**–**c** (3.51 × 10^−4^ mol) and thiol **2a**–**e** (3.51 × 10^−4^ mol, 7.01 × 10^−4^ mol, or 1.05 × 10^−3^ mol) or dithiol **3a** (3.51 × 10^−4^ mol) were added. The reaction mixture was stirred at RT for 5–24 h. The solvent was then evaporated under vacuum and the residue was purified by column chromatography on silica gel using n-hexane or a 2:1 *v/v* mixture of *n*-hexane and DCM as eluents. The evaporation of the solvents afforded analytically pure compounds.

### 3.4. General Procedure for the Synthesis of Products ***P33***–***P35***

A 10 mL high-pressure Schlenk vessel equipped with a magnetic stirring bar and connected to the gas and vacuum line was charged with the NHC carbene precursor **NHC-1** (34.38 mg, 3.5 × 10^−5^ mol), KHMDS (7.00 mg, 3.5 × 10^−5^ mol), and acetone (2 mL). The reaction mixture was stirred at RT and, after 1 h, aldehyde **1a**–**c** (50 µL, 3.51 × 10^−4^ mol) and thiol **2a** or **2d** (3.51 × 10^−4^ mol) were added. The reaction mixture was stirred at RT for 5–8 h. Next, a second thiol, **2d** or **2c** (3.51 × 10^−4^ mol), was added. The reaction mixture was stirred at RT for 14–16 h. All the products were purified by column chromatography on silica gel using n-hexane or a 2:1 *v/v* mixture of *n*-hexane and DCM as eluents. The evaporation of the solvents afforded analytically pure compounds.

### 3.5. Synthesis of Product ***P1*** on a Preparative Scale

A 10 mL high-pressure Schlenk vessel equipped with a magnetic stirring bar and connected to a gas and vacuum line was charged under argon with dry acetone (3 mL), **NHC-1** (172.06 mg, 1.75 × 10^−4^ mol) and KHMDS (35.01 mg, 1.75 × 10^−4^ mol). The reaction mixture was stirred at RT for 1 h. After this time, octynal **1a** (500 µL, 3.51 × 10^−3^ mol) and benzyl mercaptan **2a** (410.90 µL, 3.51 × 10^−3^ mol) were added. The reaction mixture was stirred at RT for 24 h. Then, the solvent was then evaporated under vacuum and the residue was purified by column chromatography (silica gel 60/2:1 *v/v* mixture of *n*-hexane and DCM). The evaporation of the solvents produced an analytically pure product (yellow oil, 0.74 g, 86%).

## 4. Conclusions

To sum up, a new practical method for the synthesis of mono- and multifunctional organosulfur carbonyl compounds, based on the processes of hydrothiolation or hydrothiolation/thioesterification of ynals with thiols and catalyzed by bulky NHC carbene, was designed and optimized. By controlling the amounts of reagents, we have shown that, at first, the triple bond C≡C is activated, which leads to the formation of SMA adducts. It is succeeded by the nucleophilic substitution of the carbonyl atom with the hydrogenation of the C=C bond, leading to the creation of thioesters. The proposed method of synthesis also permits the introduction of two different sulfur groups into the ynals’ structure and leads to cyclic products as a result of the reaction of propargyl aldehydes with dithiols. For all combinations of reagents tested, the reactions proceeded effectively, with high chemoselectivity, leading to the exclusive formation of the expected materials. In the study presented, thirty-six sulfur-based derivatives were obtained and characterized by spectroscopic methods. Taking into account the combination of desirable features, such as high atom economy (the lack of by-products, equimolar ratios of substrates, ease of product isolation, and purification), operational simplicity (one-step process), high chemo-selectivity, mild reaction conditions (metal-free catalyst, green solvent, and room temperature), and commercial availability of the reagents, this reaction system is attractive for the synthesis of a new class of organosulfur materials with potential for practical applications, which perfectly cooperate with the rules of green chemistry [53]. Moreover, in light of the recent Nobel Prize for chemistry, the proposed method shows the nucleophilic addition to activated alkynes is congruent with Click Chemistry criteria [54].

## Figures and Tables

**Table 1:** Optimization of the reaction conditions.

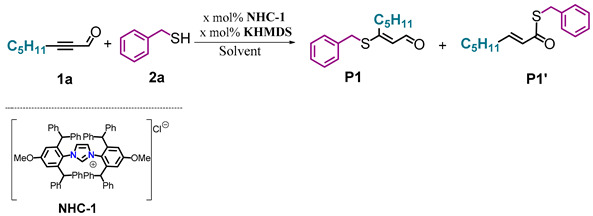						
Entry	Solvent	Temp. [°C]	NHC-1 [mol%]	Time ^[e]^ [h]	Conv. of 1a [%]	P1:P1’ ^[f]^ [%]
1	DCE	RT	5	48	3	-
2	DCM	RT	5	48	4	-
3	*n*-hexane	RT	5	24	2	-
4	MTBE	RT	5	24	98	65:35
5	Toluene	RT	5	48	3	-
6	Toluene	40	5	48	65	50:50
7	Toluene	60	5	48	95	40:60
8	Acetone	60	5	4	100	90:10
9	Acetone	40	5	5	100	96:4
**10**	**Acetone**	**RT**	**5**	**6**	**100**	**100:0**
11	Acetone	RT	2.5	24	91	100:0
12 ^[a]^	Acetone	RT	5	48	15	90:10
13 ^[b]^	Acetone	RT	-	48	0	-
14 ^[c]^	Acetone	RT	5	48	0	-
15 ^[d]^	Acetone	RT	5	6	95	100:0
16	-	RT	5	5	80	60:40

Reaction conditions: argon, [**1a**]:[**2a**] = 1:1, **NHC-1** = IPr*^OMe^·HCl, [**NHC-1**]:[KHMDS] = 1:1; ^[a]^ reaction conducted in air; ^[b]^ reaction conducted without **NHC-1**; ^[c]^ reaction conducted without KHMDS; ^[d]^ free carbene was added; ^[e]^ determined by GC-MS analysis; ^[f]^ determined by ^1^H NMR spectroscopic analysis.

## Data Availability

All data generated or analyzed during this study are included in this published article (and its Supplementary Information files: Analytical data of isolated products and catalyst, NMR spectra of isolated products: Figures S1–S78, and XRD analysis: Table S1, Figure S79–S81, for Figure S82 see ESI).

## Supplementary Materials

The following supporting information can be downloaded at: https://www.mdpi.com/article/10.3390/ijms25179201/s1. References [55,56,57,58,59] are cited in the supplementary materials.
