# Supplementary material for: Eco-Friendly Functionalization of Ynals with Thiols under Mild Conditions
Source: Int J Mol Sci. 2024 Aug 24;25(17):9201. doi: 10.3390/ijms25179201 (PMC11395323; doi:10.3390/ijms25179201)
Supplement: Supplementary file 1 [file ijms-25-09201-s001.zip › ijms-3152404-supplementary.pdf]

Supporting Information

for

Eco-friendly Functionalization of Ynals with Thiols under Mild Conditions

Kamil Hanek and Patrycja Żak[a,*]

[a] *Department of Organometallic Chemistry, Faculty of Chemistry, Adam Mickiewicz University in Poznan, Uniwersytetu Poznańskiego 8, 61-614 Poznań, Poland. E-mail: pkw@amu.edu.pl*

CONTENTS:

**1.**	**Analytical data of compounds**	**S-2**
	1.1. Analytical data of ***NHC-1***	S-2
	1.2. Analytical data of isolated products ***P1-P36***	S-2
**2.**	**NMR spectra of compounds**	**S-11**
	2.1. NMR spectra of ***NHC-1***	S-11
	2.2. NMR spectra of isolated products ***P1-P36***	S-12
**3.**	**XRD analyses**	**S-52**
**4.**	**References**	**S-54**

1. Analytical data of compounds

*1.1. Analytical data of **NHC-1***

Precursor of NHC-1	
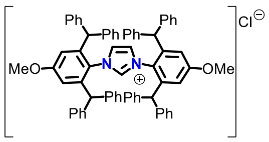 **NHC-1**	White solid, isolated yield: 85%; ^{1}H NMR (400 MHz, $CDCl_3$, 296K): δ (ppm) 3.51 (s, 6H, OCH_3), 5.30 (s, 4H, $CHPh_2$), 5.41 (s, 2H, $CH^{4,5}$ Im), 6.46 (s, 4H, CH_{Ar}), 6.83 (d, 8H, J_{HH} = 6.8 Hz, CH_{Ar}), 7.17 – 7.22 (m, 18H, CH_{Ar}), 7.25 - 7.32 (m, 16H, CH_{Ar}), 12.89 (s, 1H, CH^2 Im); ^{13}C NMR (100 MHz, $CDCl_3$, 296K): δ (ppm) 51.55 ($CHPh_2$), 55.19 (OCH_3), 115.61 (CH_{Ar}), 123.44 (NCH), 125.28 (CH_{Ar}), 126.93(CH_{Ar}), 127.01 (CH_{Ar}), 128.57 (CH_{Ar}), 128.67(CH_{Ar}), 129.14 (CH_{Ar}), 130.06 (CH_{Ar}), 141.73 (N-C_{IVAr}), 142.35 (C_{IVAr}), 142.68 (N-CH-N), 160.78 (O-$C_{IVp\text{-}Ar}$). These NMR data matched those reported in the literature.[S1]

*1.2. Analytical data of isolated products **P1-P36***

Mono-functionalized products P1-P15	
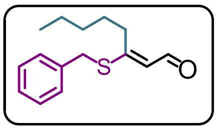 **Product P1**	Yellow oil, isolated yield: 95%; ^{1}H NMR (400 MHz, $CDCl_3$, 296K): δ (ppm) 0.91 (t, 3H, J_{HH} = 7.1 Hz, CH_3), 1.31-1.35 (m, 4H, CH_2), 1.58-1.62 (m, 2H, CH_2), 2.45-2.50 (m, 2H, CH_2), 4.07 (s, 2H, SCH_2), 6.06 (dt, 1H, J_{HH} = 6.9, 0.9 Hz, CH), 7.31-7.35 (m, 5H, C_6H_5), 10.00 (d, 1H, J_{HH} = 6.9 Hz, CH); ^{13}C NMR (100 MHz, $CDCl_3$, 296K): δ (ppm) 13.87 (CH_3), 22.27 (CH_2), 31.02 (CH_2), 31.37 (CH_2), 33.10 (CH_2), 36.46 (SCH_2), 107.04, 120.76, 127.81, 128.84 (d, J = 8.2 Hz), 134.25, 169.49, 186.70 (CO); MS m/z (rel, intensity): 57.10 (10), 91.20 (81), 95.10 (10), 157.00 (100), 158.00 (10), 248.00 (5, M^+); MS (ESI+): m/z 271 $[M+Na]^+$.
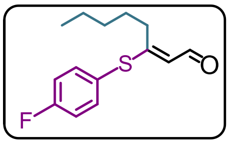 **Product P2**	Orange oil, isolated yield: 92%; ^{1}H NMR (400 MHz, $CDCl_3$, 296K): δ (ppm) 0.82 (t, 3H, J_{HH} = 7.1 Hz, CH_3), 1.10-1.22 (m, 4H, CH_2), 1.32-1.50 (m, 2H, CH_2), 2.13-2.22 (m, 2H, CH_2), 6.16 (d, 1H, J_{HH} = 6.6 Hz, CH), 7.07-7.11 (m, 2H, C_6H_4-F), 7.45-7.49 (m, 2H, C_6H_4-F), 10.12 (d, 1H, J_{HH} = 6.6 Hz, CH); ^{13}C NMR (100 MHz, $CDCl_3$, 296K): δ (ppm) 13.81 (CH_3), 22.18 (CH_2), 28.28 (CH_2), 30.89 (CH_2), 36.88 (CH_2), 116.62 (d, J = 22.1 Hz), 121.81, 127.03, 136.35 (d, J = 8.5 Hz), 137.72, 137.81, 163.15 (d, J = 216.7 Hz), 189.78 (CO); MS m/z (rel, intensity): 53.10 (23), 81.10 (42), 108.20 (16), 125.30 (32), 181.20 (14), 196.20 (18), 209.20 (32), 223.20 (15), 252.10 (35, M^+).
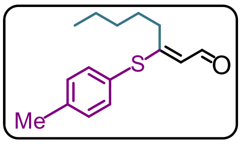 **Product P3**	Orange oil, isolated yield: 94%; ^{1}H NMR (400 MHz, $CDCl_3$, 296K): δ (ppm) 0.82 (t, 3H, J_{HH} = 7.1 Hz, CH_3), 1.11-1.20 (m, 4H, CH_2), 1.43-1.47 (m, 2H, CH_2), 2.16-2.21 (m, 2H, CH_2), 2.38 (s, 3H, CH_3), 6.14 (dt, 1H, J_{HH} = 6.9, 0.9 Hz, CH), 7.18 (d, 2H, J_{HH} = 7.9 Hz, C_6H_4-CH_3), 7.36 (d, 2H, J_{HH} = 8.0 Hz, C_6H_4-CH_3), 10.14 (d, 1H, J_{HH} = 6.9 Hz, CH); ^{13}C NMR (100 MHz, $CDCl_3$, 296K): δ (ppm) 13.82 (CH_3), 21.23 (CH_3), 22.20 (CH_2), 28.33 (CH_2), 30.89 (CH_2), 36.92 (CH_2), 112.68, 126.95, 130.12, 134.23,

	139.44, 165.24, 190.01 (*C*O); MS m/z (rel, intensity):53.10 (4), 81.10 (5) 91.30 (10), 123.20 (8), 124.20 (25), 125.20 (6), 149.30 (3) 247.40 (8), 248.30 (35, M^+).
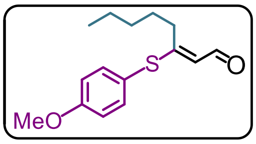 **Product P4**	Yellow oil, isolated yield: 97%; ^{1}H NMR (400 MHz, $CDCl_3$, 296K): δ (ppm) 0.82 (t, 3H, J_{HH} = 7.1 Hz, CH_3), 1.11-1.20 (m, 4H, CH_2), 1.36-1.47 (m, 4H, CH_2), 2.13-2.19 (m, 2H, CH_2), 3.84 (s, 3H, OCH_3), 6.10 (d, 1H, J_{HH} = 6.8 Hz, C*H*), 6.91 (d, 2H, J_{HH} = 8.7 Hz, C_6H_4-OCH_3), 7.41 (d, 2H, J_{HH} = 8.7 Hz, C_6H_4-OCH_3), 10.13 (d, 1H, J_{HH} = 6.8 Hz, C*H*); ^{13}C NMR (100 MHz, $CDCl_3$, 296K): δ (ppm) 13.82 (CH_3), 22.19 (CH_2), 28.34 (CH_2), 30.92 (CH_2), 36.75 (CH_2), 55.39 (OCH_3), 114.89, 125.88, 134.19, 136.30, 160.62, 189.73 (*C*O); MS m/z (rel, intensity): 53.10 (8), 108.20 (16), 121.20 (8), 125.20 (50), 139.30 (7), 140.20 (19), 247.20 (7) 263.30 (22), 264.20 (100, M^+).
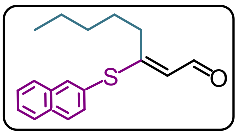 **Product P5**	White solid, isolated yield: 89%; ^{1}H NMR (400 MHz, $CDCl_3$, 296K): δ (ppm) 0.95 (t, 3H, J_{HH} = 7.1 Hz, CH_3), 1.36-1.44 (m, 4H, CH_2), 1.77-1.84 (m, 2H, CH_2), 2.80-2.85 (m, 2H, CH_2), 5.44 (d, 1H, J_{HH} = 7.9 Hz, C*H*), 7.45-7.48 (m, 1H, C*H*), 7.53-7.59 (m, 3H, C*H*), 7.82-7.90 (m, 4H, C*H*), 8.02-8.03 (m, 1H, C*H*), 9.79 (d, 1H, J_{HH} = 7.9 Hz, C*H*); ^{13}C NMR (100 MHz, $CDCl_3$, 296K): δ (ppm) 13.88 (CH_3), 22.30 (CH_2), 28.32 (CH_2), 30.82 (CH_2), 32.52 (CH_2), 122.12, 126.90, 127.66, 127.93, 129.70, 131.21, 133.75, 134.13, 135.77, 137.40, 170.95, 186.97 (*C*O); MS m/z (rel, intensity): 53.20 (29), 81.20 (38), 115.30 (47), 128.30 (47), 159.30 (20), 160.30 (100), 161.20 (26), 185.30 (30), 227.30 (17), 241.30 (15), 251.30 (46), 255.30 (39), 284.20 (51, M^+).
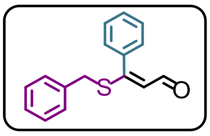 **Product P6**	Red solid, isolated yield: 90%; ^{1}H NMR (400 MHz, $CDCl_3$, 296K): δ (ppm) 4.10 (s, 2H, SCH_2), 6.17 (d, 1H, J_{HH} = 7.6 Hz, C*H*), 7.34-7.37 (m, 4H, C_6H_5), 7.40-7.46 (m, 5H, C_6H_5), 9.28 (d, 1H, J_{HH} = 7.6 Hz, C*H*); ^{13}C NMR (100 MHz, $CDCl_3$, 296K): δ (ppm) 37.62 (SCH_2), 122.94, 127.87, 128.52, 128.85, 128.92, 129.47, 130.24, 134.35, 134.90, 167.27, 189.76 (*C*O); MS m/z (rel, intensity): 50.10 (5), 65.20 (11), 77.20 (5), 91.30 (36), 121.20 (6), 135.20 (4) 163.10 (100), 164.00 (10), 165.00 (5), 254.20 (8, M^+).
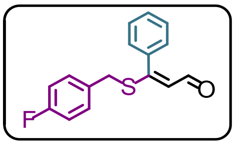 **Product P7**	Yellow oil, isolated yield: 95%; ^{1}H NMR (400 MHz, $CDCl_3$, 296K): δ (ppm) 5.84 (d, 1H, J_{HH} = 7.9 Hz, C*H*), 7.15-7.17 (m, 2H, C_6H_4-F), 7.45-7.52 (m, 5H, C_6H_5), 7.54-7.56 (m, 2H, C_6H_4-F), 9.27 (d, J_{HH} = 7.9 Hz, C*H*); ^{13}C NMR (100 MHz, $CDCl_3$, 296K): δ (ppm) 117.38 (d, J = 22.1 Hz), 123.41, 128.69, 129.48, 130.42, 134.40, 137.58 (d, J = 8.7 Hz), 163.97 (d, J = 252.2 Hz), 168.80, 189.82 (*C*O); ^{29}F NMR (376 MHz, $CDCl_3$, 296K): δ (ppm) -116.57; MS m/z (rel, intensity): 51.00 (9), 77.20 (17), 83.10 (9), 102.20 (6), 103.10 (33), 127.20 (8) 131.00 (100), 132.00 (10), 259.00 (1, M^+).

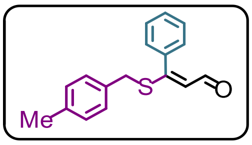<br>**Product P8**	Yellow oil, isolated yield: 91%; ^{1}H NMR (400 MHz, $CDCl_3$, 296K): δ (ppm) 2.41 (s, 3H, CH_3), 5.84 (d, 1H, J_{HH} = 8.0 Hz, CH), 7.26-7.29 (m, 2H, C_6H_4-CH_3), 7.43-7.48 (m, 5H, C_6H_5), 7.51-7.53 (m, 2H, C_6H_4-CH_3), 9.26 (d, J_{HH} = 8.0 Hz, CH); ^{13}C NMR (100 MHz, $CDCl_3$, 296K): δ (ppm) 21.34 (CH_3), 123.17, 125.30, 128.53, 129.46, 130.24, 134.78, 135.29, 140.85, 169.70, 189.92 (CO); IR: ν (cm^{-1}): 1658 (C=O); MS m/z (rel, intensity): 51.00 (6), 77.20 (12), 91.20 (3), 102.20 (4), 103.10 (22), 104.00 (3), 123.10 (3) 131.00 (100), 132.00 (10), 255.00 (2, M^+).
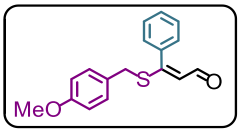<br>**Product P9**	Orange solid, isolated yield: 93%; ^{1}H NMR (400 MHz, $CDCl_3$, 296K): δ (ppm) 3.84 (s, 3H, OCH_3), 5.62 (d, 1H, J_{HH} = 8.0 Hz, CH), 6.97 (d, 2H, J_{HH} = 8.9 Hz, C_6H_4-OCH_3), 7.42-7.43 (m, 1H, C_6H_5), 7.44-7.46 (m, 3H, C_6H_5), 7.47-7.48 (m, 1H, C_6H_5), 7.50-7.52 (m, 2H, C_6H_4-OCH_3), 9.27 (d, J_{HH} = 8.0 Hz, CH); ^{13}C NMR (100 MHz, $CDCl_3$, 296K): δ (ppm) 55.44 (OCH_3), 115.65, 119.33, 123.07, 128.53, 129.42, 130.23, 134.74, 136.95, 161.35, 170.22, 189.85 (CO); MS m/z (rel, intensity): 50.10 (6), 51.10 (9), 77.20 (17), 103.10 (27),125.20 (8), 131.10 (100), 132.10 (10), 139.10 (12), 140.10 (15), 271.10 (1, M^+).
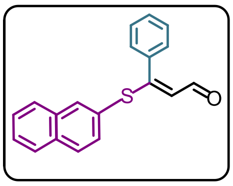<br>**Product P10**	Orange solid, isolated yield: 90%; ^{1}H NMR (400 MHz, $CDCl_3$, 296K): δ (ppm) 5.68 (d, 1H, J_{HH} = 8.0 Hz, CH), 7.45-7.50 (m, 3H, CH), 7.56-7.62 (m, 5H, C_6H_5), 7.85-7.93 (m, 3H, CH), 8.12 (s, 1H, CH), 9.29 (d, 1H, J_{HH} = 8.0 Hz, CH); ^{13}C NMR (100 MHz, $CDCl_3$, 296K): δ (ppm) 126.56, 127.14, 127.24, 127.56, 127.81, 127.86, 129.40, 129.79, 133.26, 133.34, 133.67, 156.62, 189.71 (CO); MS (ESI+): m/z 313 $[M+Na]^+$.
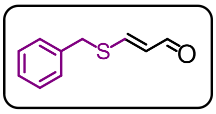<br>**Product P11**	Yellow oil, isolated yield: 92%; ^{1}H NMR (400 MHz, $CDCl_3$, 296K): δ (ppm) 4.08 (s, 2H, SCH_2), 6.22 (dd, 1H, J_{HH} = 15.1, 7.6 Hz, CH), 7.30-7.37 (m, 5H, CH), 7.56 (d, 1H, J_{HH} = 15.1 Hz, CH), 9.37 (d, 1H, J_{HH} = 7.6 Hz, CH); ^{13}C NMR (100 MHz, $CDCl_3$, 296K): δ (ppm) 36.81 (SCH_2), 126.41, 127.96, 128.76, 128.92, 134.72, 155.44, 189.78 (CO); MS m/z (rel, intensity): 58.00 (7), 65.00 (22), 87.00 (27), 89.00 (5), 91.00 (100), 91.90 (8), 118.00 (6), 145.00 (8), 177.80 (8, M^+).
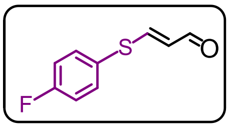<br>**Product P12**	Orange oil, isolated yield: 91%; ^{1}H NMR (400 MHz, $CDCl_3$, 296K): δ (ppm) 5.89 (dd, 1H, J_{HH} = 15.0, 7.7 Hz, CH), 7.13-7.16 (m, 2H, C_6H_4-F), 7.48-7.51 (m, 2H, C_6H_4-F), 7.52 (d, 1H, J_{HH} = 15.0 Hz, CH), 9.43 (d, 1H, J_{HH} = 7.7 Hz, CH); ^{13}C NMR (100 MHz, $CDCl_3$, 296K): δ (ppm) 117.33, 126.99, 135.93, 156.54, 163.71 (d, J = 251.7 Hz), 189.56 (CO); MS m/z (rel, intensity): 57.10 (41), 58.00 (46), 83.10 (62), 86.00 (100), 87.00 (34), 109.10 (40), 127.00 (55), 128.00 (94), 153.10 (26), 182.00 (88, M^+).

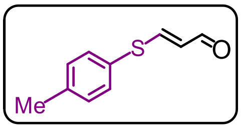<br>**Product P13**	Orange oil, isolated yield: 92%; ^{1}H NMR (400 MHz, $CDCl_3$, 296K): δ (ppm) 2.39 (s, 3H, CH_3), 5.91 (dd, 1H, J_{HH} = 14.9, 7.8 Hz, CH), 7.24 (d, 2H, J_{HH} = 7.8 Hz, C_6H_4-CH_3), 7.37 (d, 2H, J_{HH} = 7.8 Hz, C_6H_4-CH_3), 7.65 (d, 1H, J_{HH} = 14.9 Hz, CH), 9.41 (d, 1H, J_{HH} = 7.8 Hz, CH); ^{13}C NMR (100 MHz, $CDCl_3$, 296K): δ (ppm) 21.22 (CH_3), 125.68, 126.75, 130.64, 133.48, 140.24, 157.50, 189.73 (CO); MS m/z (rel, intensity): 45.10 (22), 58.00 (20), 86.00 (24), 91.20 (97), 92.00 (26), 124.10 (30), 134.20 (19), 163.20 (43), 177.20 (83), 177.90 (100, M^+).
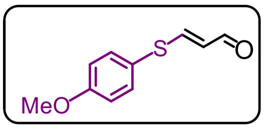<br>**Product P14**	Yellow oil, isolated yield: 93%; ^{1}H NMR (400 MHz, $CDCl_3$, 296K): δ (ppm) 3.83 (s, 3H, OCH_3), 5.84 (dd, 1H, J_{HH} = 14.9, 7.8 Hz, CH), 6.95 (d, 2H, J_{HH} = 8.9 Hz, C_6H_4-OCH_3), 7.40 (d, 2H, J_{HH} = 8.9 Hz, C_6H_4-OCH_3), 7.63 (d, 1H, J_{HH} = 14.9 Hz, CH), 9.41 (d, 1H, J_{HH} = 7.8 Hz, CH); ^{13}C NMR (100 MHz, $CDCl_3$, 296K): δ (ppm) 55.41 (OCH_3), 115.50, 119.42, 126.57, 135.52, 157.28, 161.07, 189.58 (CO); MS m/z (rel, intensity): 63.30 (3), 87.20 (3), 107.30 (4), 108.30 (11), 109.20 (2) 140.10 (3), 189.90 (3), 191.20 (4), 193.10 (18), 194.80 (100, M^+).
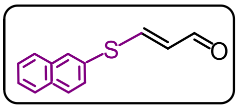<br>**Product P15**	Beige solid, isolated yield: 89%; ^{1}H NMR (400 MHz, $CDCl_3$, 296K): δ (ppm) 5.99 (dd, 2H, J_{HH} = 15.0, 7.7 Hz, CH), 7.50-7.52 (m, 1H, CH), 7.56-7.59 (m, 2H, CH), 7.74 (d, 1H, J_{HH} = 7.7 Hz, CH), 7.84-7.92 (m, 3H, CH), 8.02-8.04 (m, 1H, CH), 9.45 (d, 1H, J_{HH} = 7.7 Hz, CH); ^{13}C NMR (100 MHz, $CDCl_3$, 296K): δ (ppm) 127.15, 127.24, 127.56, 127.81, 128.02, 129.39, 129.78, 133.26, 133.67, 156.62, 189.72 (CO); MS (ESI+): m/z 237 $[M+Na]^+$.
**Multi-functionalized products P16-P36**	
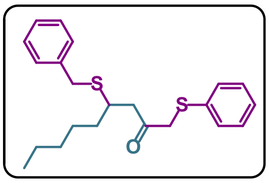<br>**Product P16**	Yellow oil, isolated yield: 97%; ^{1}H NMR (400 MHz, $CDCl_3$, 296K): δ (ppm) 0.85 (t, 3H, J_{HH} = 7.9 Hz, CH_3), 1.12-1.30 (m, 6H, CH_2), 1.45-1.53 (m, 2H, CH_2), 2.75-2.85 (m, 2H, CH_2), 3.02-3.08 (m, 1H, CH), 3.71 (s, 2H, SCH_2), 4.14 (s, 2H, SCH_2), 7.20-7.25 (m, 2H, C_6H_5), 7.27-7.32 (m, 8H, C_6H_5); ^{13}C NMR (100 MHz, $CDCl_3$, 296K): δ (ppm) 13.98 (CH_3), 22.45 (CH_2), 26.14 (CH_2), 31.38 (CH_2), 33.33 (CH_2), 34.57 (SCH_2), 35.69 (SCH_2), 41.79 (CH_2), 49.77 (CH), 126.98, 127.25, 128.44, 128.59, 128.79, 128.92, 137.45, 138.19, 196.78 (CO); MS m/z (rel, intensity): 55.00 (55), 65.00 (20), 91.20 (100), 124.10 (19), 125.00 (56), 249.00 (4, M^+-S-CH_2-C_6H_6), 281.00 (3, M^+- CH_2-C_6H_5); MS (ESI+): m/z 411 $[M+K]^+$.

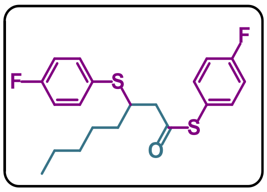 **Product P17**	Yellow oil, isolated yield: 94%; ^{1}H NMR (400 MHz, $CDCl_3$, 296K): δ (ppm) 0.89 (t, 3H, J_{HH} = 7.0 Hz, CH_3), 1.25-1.39 (m, 4H, CH_2), 1.46-1.66 (m, 4H, CH_2), 2.80-2.91 (m, 2H, CH_2), 3.44-3.51 (m, 1H, CH), 7.20-7.25 (m, 2H, C_6H_5), 6.99-7.05 (m, 2H, C_6H_4-F), 7.08-7.13 (m, 2H, C_6H_4-F), 7.33-7.38 (m, 2H, C_6H_4-F), 7.43-7.48 (m, 2H, C_6H_4-F); ^{13}C NMR (100 MHz, $CDCl_3$, 296K): δ (ppm) 13.97 (CH_3), 22.46 (CH_2), 26.42 (CH_2), 31.40 (CH_2), 34.25 (CH_2), 46.01 (CH_2), 48.82 (CH), 116.08 (d, J = 21.8 Hz), 116.46 (d, J = 21.8 Hz), 122.67 (d, J = 3.5 Hz), 128.64 (d, J = 3.5 Hz), 135.70 (d, J = 8.2 Hz), 136.46 (d, J = 8.6 Hz), 162.59 (d, J = 248.2 Hz), 163.47 (d, J = 250.3 Hz), 195.45 (CO); ^{29}F NMR (376 MHz, $CDCl_3$, 296K): δ (ppm) -110.90, -113.55; MS m/z (rel, intensity): 55.20 (74), 69.20 (12), 83.20 (16), 108.20 (19), 124.20 (16), 125.20 (100), 127.00 (25), 129.10 (35), 211.20 (22), 253.20 (10, M^{+}- S-C_6H_4-F).
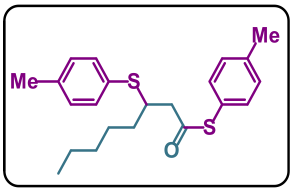 **Product P18**	Yellow oil, isolated yield: 97%; ^{1}H NMR (400 MHz, $CDCl_3$, 296K): δ (ppm) 0.91 (t, 3H, J_{HH} = 7.0 Hz, CH_3), 1.26-1.37 (m, 4H, CH_2), 1.47-1.68 (m, 4H, CH_2), 2.35 (s, 3H, CH_3), 2.39 (s, 3H, CH_3), 2.79-2.86 (m, 1H, CH_2), 2.90-2.96 (m, 1H, CH_2), 3.50-3.57 (m, 1H, CH), 7.14 (d, 2H, J_{HH} = 8.0 Hz, C_6H_4-CH_3), 7.22 (d, 2H, J_{HH} = 8.0 Hz, C_6H_4-CH_3), 7.28 (d, 2H, J_{HH} = 8.0 Hz, C_6H_4-CH_3), 7.37 (d, 2H, J_{HH} = 8.0 Hz, C_6H_4-CH_3); ^{13}C NMR (100 MHz, $CDCl_3$, 296K): δ (ppm) 13.98 (CH_3), 21.07 (CH_3), 21.28 (CH_3), 22.46 (CH_2), 26.43 (CH_2), 31.44 (CH_2), 34.12 (CH_2), 45.46 (CH_2), 48.98 (CH), 123.99, 129.69, 129.95, 133.37, 134.31, 137.55, 139.64, 196.03 (CO); MS m/z (rel, intensity): 45.00 (18), 53.10 (18), 55.10 (76), 79.20 (13), 91.20 (63), 123.20 (29), 124.00 (87), 125.00 (100), 207.10 (9), 249.10 (31, M^{+}- S-C_6H_4-Me).
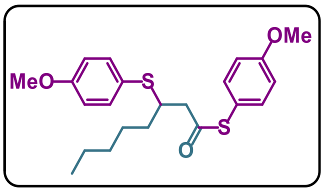 **Product P19**	Yellow oil, isolated yield: 95%; ^{1}H NMR (400 MHz, $CDCl_3$, 296K): δ (ppm) 0.89 (t, 3H, J_{HH} = 7.0 Hz, CH_3), 1.24-1.33 (m, 4H, CH_2), 1.47-1.60 (m, 4H, CH_2), 2.73-2.27 (m, 1H, CH_2), 2.84-2.90 (m, 1H, CH_2), 3.36-3.42 (m, 1H, CH), 3.81 (s, 3H, OCH_3), 3.82 (s, 3H, OCH_3), 6.86 (d, 2H, J_{HH} = 8.9 Hz, C_6H_4-OCH_3), 6.93 (d, 2H, J_{HH} = 8.9 Hz, C_6H_4-OCH_3), 7.29 (d, 2H, J_{HH} = 8.9 Hz, C_6H_4-OCH_3), 7.42 (d, 2H, J_{HH} = 8.9 Hz, C_6H_4-OCH_3); ^{13}C NMR (100 MHz, $CDCl_3$, 296K): δ (ppm) 13.99 (CH_3), 22.50 (CH_2), 26.46 (CH_2), 31.49 (CH_2), 34.12 (CH_2), 46.18 (CH_2), 48.63 (CH), 55.30 (OCH_3), 55.33 (OCH_3), 114.43, 114.85, 118.36, 123.72, 136.00, 136.14, 159.76, 160.68, 196.57 (CO); MS m/z (rel, intensity): 53.10 (33), 55.20 (55), 69.20 (19), 81.20 (13) 97.20 (33), 124.30 (15), 125.30 (69), 139.30 (27), 140.20 (100), 265.30 (6, M^{+}- S-C_6H_4-OMe).

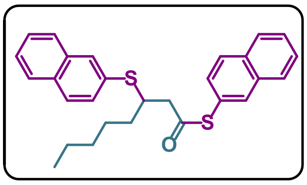 **Product P20**	Yellow oil, isolated yield: 90%; ^{1}H NMR (400 MHz, $CDCl_3$, 296K): δ (ppm) 0.92 (t, 3H, J_{HH} = 6.9 Hz, CH_3), 1.31-1.37 (m, 4H, CH_2), 1.55-1.77 (m, 4H, CH_2), 2.92-2.99 (m, 1H, CH_2), 2.84-2.90 (m, 1H, CH_2), 3.76-3.83 (m, 1H, C*H*), 7.38 (dd, 1H, J_{HH} = 8.5, 1.8 Hz, C*H*), 7.49-7.54 (m, 4H, C*H*), 7.56 (d, 1H, J_{HH} = 1.8 Hz, C*H*), 7.78-7.87 (m, 7H, C*H*), 7.97 (d, 1H, J_{HH} = 1.8 Hz, C*H*); ^{13}C NMR (100 MHz, $CDCl_3$, 296K): δ (ppm) 13.99 (CH_3), 22.49 (CH_2), 26.52 (CH_3), 31.51 (CH_2), 34.37 (CH_2), 45.09 (CH_2), 49.23 (*C*H), 127.74, 126.18, 126.50 (d, *J* = 4.4 Hz), 127.11, 127.42, 127.69 (d, *J* = 8.5 Hz), 127.88, 128.53, 128.72, 129.80, 130.73, 131.18, 131.47, 132.34, 133.29, 133.46, 133.62, 134.23, 195.74 (*C*O); MS m/z (rel, intensity): 51.00 (18), 65.00 (13), 77.20 (26), 81.10 (24), 103.10 (31), 116.20 (17), 128.20 (15), 131.20 (100), 160.20 (39), 285.20 (4, M^{+}- S-C_5H_3-C_5H_4).
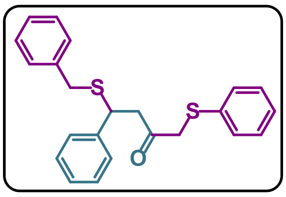 **Product P21**	Yellow oil, isolated yield: 95%; ^{1}H NMR (400 MHz, $CDCl_3$, 296K): δ (ppm) 3.07-3.13 (m, 2H, SCH_2), 3.47-3.59 (m, 2H, CH_2), 4.07 (s, 2H, SCH_2), 4.58 (t, 1H, J_{HH} = 7.7 Hz, C*H*), 7.14-7.24 (m, 5H, C_6H_5), 7.24-7.26 (m, 2H, C_6H_5), 7.27-7.30 (m, 4H, C_6H_5), 7.31-7.37 (m, 4H, C_6H_5); ^{13}C NMR (100 MHz, $CDCl_3$, 296K): δ (ppm) 33.22 (SCH_2), 35.79 (SCH_2), 45.24 (CH_2), 49.93 (*C*H), 127.00, 127.15, 127.51, 127.89, 128.40, 128.48, 128.54, 128.67, 128.88, 137.18, 137.56, 140.46, 195.39 (*C*O); MS m/z (rel, intensity): 45.00 (8), 50.10 (7), 51.10 (10), 65.10 (12), 77.20 (18), 91.20 (36), 103.20 (30), 131.20 (100), 132.20 (9), 252.20 (5, M^{+}- S-CH_2-C_6H_5).
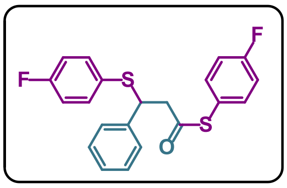 **Product P22**	White solid, isolated yield: 98%; ^{1}H NMR (400 MHz, $CDCl_3$, 296K): δ (ppm) 3.19-3.26 (m, 2H, CH_2), 4.61 (t, 1H, J_{HH} = 7.6 Hz, C*H*), 6.90-7.00 (m, 2H, C_6H_4-F), 7.03-7.09 (m, 2H, C_6H_4-F), 7.17-7.21 (m, 2H, C_6H_4-F), 7.22-7.26 (m, 4H, C_6H_4-F and C_6H_5), 7.27-7.31 (m, 3H, C_6H_5); ^{13}C NMR (100 MHz, $CDCl_3$, 296K): δ (ppm) 48.95 (CH_2), 49.98 (*C*H), 115.99 (d, *J* = 21.9 Hz), 116.46 (d, *J* = 22.2 Hz), 127.74, 128.51, 136.27 (d, *J* = 8.4 Hz), 136.44 (d, *J* = 8.7 Hz), 139.73, 162.89 (d, *J* = 248.8 Hz), 163.52 (d, *J* = 250.3 Hz), 194.49 (*C*O); ^{29}F NMR (376 MHz, $CDCl_3$, 296K): δ (ppm) -110.83, -112.77; MS m/z (rel, intensity): 51.10 (10), 77.20 (18), 83.20 (10), 103.20 (37), 127.20 (9), 131.00 (100), 132.00 (10), 259 (8, M^{+}-S-C_6H_4-F).
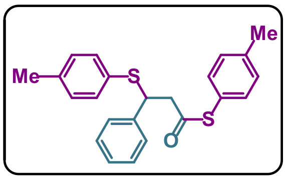 **Product P23**	White solid, isolated yield: 97%; ^{1}H NMR (400 MHz, $CDCl_3$, 296K): δ (ppm) 2.32 (s, 3H CH_3), 2.36 (s, 3H CH_3), 3.22-3.26 (m, 2H, CH_2), 4.61 (t, 1H, J_{HH} = 7.6 Hz, C*H*), 7.07 (d, 2H, J_{HH} = 7.6 Hz, C_6H_4-CH_3), 7.14-7.19 (m, 4H, C_6H_4-CH_3), 7.22-7.225 (m, 2H, C_6H_4-CH_3), 7.27-7.32 (m, 4H, C_6H_5), 7.38-7.42 (m, 1H, C_6H_5); ^{13}C NMR (100 MHz, $CDCl_3$, 296K): δ (ppm) 21.11 (CH_3), 21.27 (CH_3), 49.21 (CH_2), 49.55 (*C*H), 127.52, 127.79, 128.41, 128.95, 129.65,

	129.94, 130.02, 130.64, 133.73, 134.29, 134.54, 139.68, 141.31, 195.07 (*C*O); MS m/z (rel, intensity): 50.20 (4), 51.20 (6), 77.20 (13), 91.20 (5), 102.20 (5), 103.20 (21), 123.20 (4), 131.00 (100), 132.00 (9), 255 (6, M^{+}- S-C_6H_4-Me).
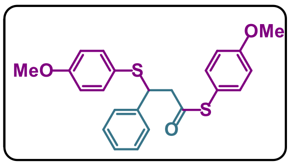 **Product P24**	Orange solid, isolated yield: 91%; ^{1}H NMR (400 MHz, $CDCl_3$, 296K): δ (ppm) 3.20-3.24 (m, 2H, CH_2), 3.78 (s, 3H, OCH_3), 3.80 (s, 3H, OCH_3), 4.68 (t, 1H, J_{HH} = 7.6 Hz, C*H*), 6.78 (d, 2H, J_{HH} = 8.9 Hz, C_6H_4-OCH_3), 6.89 (d, 2H, J_{HH} = 8.9 Hz, C_6H_4-OCH_3), 7.18-7.22 (m, 4H, C_6H_4-OCH_3), 7.23-7.25 (m, 2H, C_6H_5), 7.26-7.31 (m, 3H, C_6H_5); ^{13}C NMR (100 MHz, $CDCl_3$, 296K): δ (ppm) 31.19 (CH_2), 43.09 (*C*H), 55.30 (br s, OCH_3), 114.67, 114.82, 117.98, 124.83, 134.18, 136.01, 159.36, 160.65, 196.75 (*C*O); MS m/z (rel, intensity): 51.20 (6), 77.20 (11), 102.20 (5), 103.20 (29), 104.20 (4), 131.20 (100), 132.20 (10), 139.20 (10), 140.10 (9), 271.10 (2, M^{+} - S-C_6H_4-OMe).
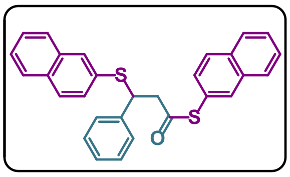 **Product P25**	Beige solid, isolated yield: 88%; ^{1}H NMR (400 MHz, $CDCl_3$, 296K): δ (ppm) 3.30-3.39 (m, 2H, CH_2), 4.90 (t, 1H, J_{HH} = 7.5 Hz, C*H*), 7.27-7.38 (m, 6H, C*H*), 7.41-7.44 (m, 1H, C*H*), 7.46-7.54 (m, 4H, C*H*), 7.71-7.87 (m, 8H, C*H*); ^{13}C NMR (100 MHz, $CDCl_3$, 296K): δ (ppm) 49.12 (CH_2), 49.49 (*C*H), 124.54, 126.39, 126.47 (d, *J* = 5.0 Hz), 127.13, 127.56, 127.64, 127.73 (d, *J* = 4.2 Hz), 127.80, 127.93, 128.47, 128.58, 128.72, 130.09, 130.69, 130.94, 132.19, 132.55, 133.30, 133.43, 133.50, 134.23, 139.87, 194.83 (*C*O); MS (ESI+): m/z 489 $[M+K]^{+}$.
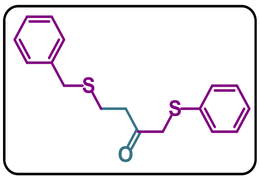 **Product P26**	Yellow oil, isolated yield: 92%; ^{1}H NMR (400 MHz, $CDCl_3$, 296K): δ (ppm) 2.71-2.74 (m, 2H, CH_2), 2.77-2.80 (m, 2H, CH_2), 3.72 (s, 2H, SCH_2), 4.14 (s, 2H, SCH_2), 7.23-7.26 (m, 8H, C_6H_5), 7.28-7.32 (m, 2H, C_6H_5); ^{13}C NMR (100 MHz, $CDCl_3$, 296K): δ (ppm) 26.54 (SCH_2), 33.27 (SCH_2), 36.45 (CH_2), 43.48 (*C*H), 127.11, 127.28, 128.57 (d, *J* = 7.8 Hz), 128.80 (d, *J* = 2.7 Hz), 137.26, 137.87, 196.86 (*C*O); MS m/z (rel, intensity): 45.00 (15), 55.00 (18), 63.00 (6), 65.00 (17), 89.00 (9), 91.00 (100), 117.00 (7), 124.00 (10), 178.00 (8, M^{+}- S-CH_2-C_6H_5); MS (ESI+): m/z 341 $[M+K]^{+}$.
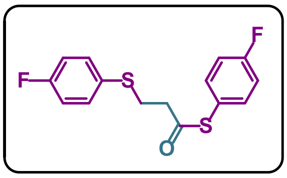 **Product P27**	Yellow oil, isolated yield: 94%; ^{1}H NMR (400 MHz, $CDCl_3$, 296K): δ (ppm) 2.87-2.94 (m, 2H, CH_2), 3.12-3.20 (m, 2H, CH_2), 7.01-7.05 (m, 2H, C_6H_4-F), 7.09-7.13 (m, 2H, C_6H_4-F), 7.21-7.24 (m, 2H, C_6H_4-F), 7.39-7.43 (m, 2H, C_6H_4-F); ^{13}C NMR (100 MHz, $CDCl_3$, 296K): δ (ppm) 30.54 (CH_2), 43.09 (CH_2), 116.25 (d, *J* = 22.1 Hz), 116.50 (d, *J* = 22.0 Hz), 133.35 (d, *J* = 8.1 Hz), 133.52 (d, *J* = 8.3 Hz), 136.48 (d, *J* = 8.1 Hz), 136.67 (d, *J* = 8.3 Hz), 162.26 (d, *J* = 247.7 Hz), 163.55 (d, *J* = 250.5 Hz), 195.47 (*C*O); ^{29}F NMR (376 MHz, $CDCl_3$, 296K): δ (ppm) -110.79, -114.29, -116.57; MS m/z (rel, intensity): 56.90 (23), 57.90 (36), 61.00 (22), 73.00

	(42), 83.00 (58), 85.80 (87), 109.90 (31), 126.90 (35), 128.00 (80), 182.80 (58, M^{+}-S-C_6H_4-F).
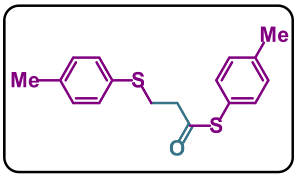<br>**Product P28**	Yellow oil, isolated yield: 96%; ^{1}H NMR (400 MHz, $CDCl_3$, 296K): δ (ppm) 2.35 (s, 3H, CH_3), 2.39 (s, 3H, CH_3), 2.88-2.95 (m, 2H, CH_2), 3.14-3.22 (m, 2H, CH_2), 7.13-7.16 (m, 2H, C_6H_4-CH_3), 7.21-7.24 (m, 2H, C_6H_4-CH_3), 7.27-7.34 (m, 4H, C_6H_4-CH_3); ^{13}C NMR (100 MHz, $CDCl_3$, 296K): δ (ppm) 21.00 (CH_3), 21.28 (CH_3), 29.89 (CH_2), 43.17 (CH_2), 123.27, 129.81, 129.99, 131.06, 134.45, 136.94, 139.73, 196.15 (*C*O); MS m/z (rel, intensity): 45.00 (16), 47.10 (8), 73.20 (18), 74.10 (4), 75.20 (31), 91.20 (4), 167.20 (100), 168.20 (14), 169.30 (5), 179.20 (7, M^{+} - S-C_6H_4-Me).
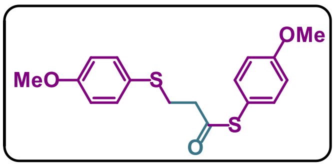<br>**Product P29**	Yellow oil, isolated yield: 95%; ^{1}H NMR (400 MHz, $CDCl_3$, 296K): δ (ppm) 2.85-2.88 (m, 2H, CH_2), 3.09-3.12 (m, 2H, CH_2), 3.81 (s, 3H, OCH_3), 3.82 (s, 3H, OCH_3), 6.87 (d, 2H, J_{HH} = 8.9 Hz, C_6H_4-OCH_3), 6.93 (d, 2H, J_{HH} = 8.9 Hz, C_6H_4-OCH_3), 7.29 (d, 2H, J_{HH} = 8.9 Hz, C_6H_4-OCH_3), 7.40 (d, 2H, J_{HH} = 8.9 Hz, C_6H_4-OCH_3); ^{13}C NMR (100 MHz, $CDCl_3$, 296K): δ (ppm) 31.19 (CH_2), 43.09 (*C*H), 55.30 (br s, OCH_3), 114.67, 114.82, 117.98, 124.83, 134.18, 136.01, 159.36, 160.65, 196.75 (*C*O); IR: ν (cm^{-1}): 1703 (C=O); MS m/z (rel, intensity): 45.10 (28), 47.00 (27), 65.20 (9) 67.20 (9), 73.20 (11), 75.10 (77), 95.10 (100), 177.20 (15.4), 185.10 (16), 195.10 (7, M^{+}- S-C_6H_4-OMe).
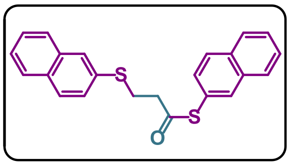<br>**Product P30**	White solid, isolated yield: 86%; ^{1}H NMR (400 MHz, $CDCl_3$, 296K): δ (ppm) 3.02-3.10 (m, 2H, CH_2), 3.34-3.40 (m, 2H, CH_2), 7.42 (dd, 1H, J_{HH} = 8.5, 1.8 Hz, C*H*), 7.48-7.56 (m, 5H, C*H*), 7.78-7.87 (m, 7H, C*H*), 7.89-7.92 (m, 1H, C*H*); ^{13}C NMR (100 MHz, $CDCl_3$, 296K): δ (ppm) 29.06 (CH_2), 43.22 (CH_2), 124.51, 126.53, 126.63, 127.17 (d, *J* = 4.5 Hz), 127.71 (d, *J* = 5.0 Hz), 127.85, 127.93, 128.33, 128.68, 128.78, 130.18, 130.73, 132.03, 132.27, 132.82, 133.31, 133.46, 133.67, 134.32, 195.87 (*C*O); MS (ESI+): m/z 413 $[M+K]^{+}$.
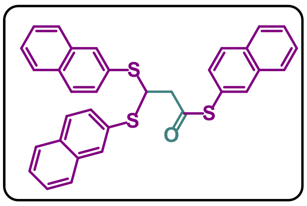<br>**Product P31**	White solid, isolated yield: 86%; ^{1}H NMR (400 MHz, $CDCl_3$, 296K): δ (ppm) 3.03-3.42 (m, 2H, CH_2), 4.90 (t, 1H, J_{HH} = 7.9 Hz, C*H*), 7.22-7.25 (m, 1H, C*H*), 7.28-7.37 (m, 5H, C*H*), 7.43 (dd, 1H, J_{HH} = 8.5, 1.8 Hz, C*H*), 7.45-7.58 (m, 5H C*H*), 7.66-7.96 (m, 9H C*H*); ^{13}C NMR (100 MHz, $CDCl_3$, 296K): δ (ppm) 49.12 (CH_2), 49.49 (*C*H), 124.54, 126.39, 126.47 (d, *J* = 4.9 Hz), 127.13, 127.24, 127.40, 127.56, 127.63, 127.73 (d, *J* = 4.3 Hz), 127.80, 127.93, 128.47, 128.58, 128.72, 128.90, 130.08, 130.68, 130.94, 132.18, 132.55, 133.30, 133.42, 133.50, 134.23, 139.86, 194.83 (*C*O); IR: ν (cm^{-1}): 1650 (C=O); MS (ESI+): m/z 572 $[M+K]^{+}$.

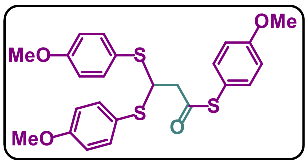 **Product P32**	Yellow oil, isolated yield: 94%; ^{1}H NMR (400 MHz, $CDCl_3$, 296K): δ (ppm) 2.77-2.79 (m, 2H, CH_2), 3.82 (s, 9H, OCH_3), 4.58 (t, 1H, J_{HH} = 7.0 Hz, CH), 6.87 (d, 6H, J_{HH} = 8.9 Hz, C_6H_4-OCH_3), 7.44 (d, 6H, J_{HH} = 8.9 Hz, C_6H_4-OCH_3); ^{13}C NMR (100 MHz, $CDCl_3$, 296K): δ (ppm) 31.22 (CH_2), 43.11 (CH), 48.34 (OCH_3), 53.41 (OCH_3) 55.32 (OCH_3), 114.63, 114.69, 114.85, 122.98, 134.13, 136.04, 136.47, 160.32, 198.89 (CO); MS (ESI+): m/z 512 $[M+K]^+$.
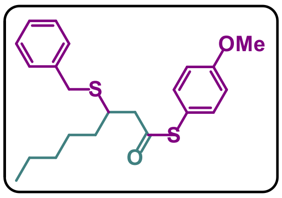 **Product P33**	Yellow oil, isolated yield: 86%; ^{1}H NMR (400 MHz, $CDCl_3$, 296K): δ (ppm) 0.89 (t, 3H, J_{HH} = 7.0 Hz, CH_3), 1.24-1.32 (m, 4H, CH_2), 1.50-1.62 (m, 4H, CH_2), 2.72-2.81 (m, 1H, CH_2), 2.84-2.90 (m, 1H, CH_2), 3.32-3.46 (m, 1H, CH), 3.81 (s, 3H, OCH_3), 3.82 (s, 2H, SCH_2), 6.86 (d, 2H, J_{HH} = 8.9 Hz, CH), 6.93 (d, 2H, J_{HH} = 8.9 Hz, CH), 7.27-7.32 (m, 3H, CH), 7.42 (d, 2H, J_{HH} = 8.7 Hz, CH); ^{13}C NMR (100 MHz, $CDCl_3$, 296K): δ (ppm) 14.02 (CH_3), 22.52 (CH_2), 26.47 (CH_2), 31.49 (CH_2), 34.08 (CH_2), 46.16 (SCH_2), 48.80 (CH_2), 55.31 (CH), 55.34 (OCH_3), 114.51, 114.84, 118.28, 123.60, 128.09, 128.26, 129.25, 129.89, 136.02, 136.19, 196.67 (CO); MS (ESI+): m/z 427 $[M+Na]^+$.
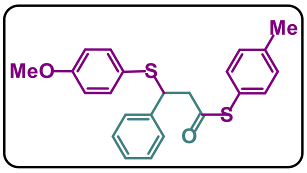 **Product P34**	Yellow solid, isolated yield: 90%; ^{1}H NMR (400 MHz, $CDCl_3$, 296K): δ (ppm) 2.29-2.37 (m, 3H, CH_3), 3.17-3.25 (m, 2H, CH_2), 3.76-3.83 (m, 3H, OCH_3), 4.54-4.69 (m, 1H, CH), 6.78 (d, 1H, J_{HH} = 8.9 Hz, CH), 6.88 (d, 1H, J_{HH} = 8.9 Hz, CH), 7.06 (d, 1H, J_{HH} = 7.8 Hz, CH), 7.12-7.25 (m, 8H, CH), 7.27-7.31 (m, 2H, CH); ^{13}C NMR (100 MHz, $CDCl_3$, 296K): δ (ppm) 21.28 (CH_3), 43.04 (CH_2), 49.54 (CH), 55.25 (OCH_3), 114.40, 114.80, 127.49 (d, J = 6.7 Hz), 127.80, 128.38 (d, J = 5.6 Hz), 129.56, 129.94, 133.72, 134.30, 135.95, 136.43, 139.69, 140.14, 159.99, 160.66, 195.09 (CO); IR: ν (cm^{-1}): 1625 (C=O); MS (ESI+): m/z 433 $[M+K]^+$.
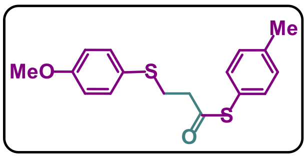 **Product P35**	Orange solid, isolated yield: 91%; ^{1}H NMR (400 MHz, $CDCl_3$, 296K): δ (ppm) 2.33-2.38 (m, 3H, CH_3), 2.84-2.92 (m, 2H, CH_2), 3.08-3.13 (m, 1H, CH_2), 3.14-3.19 (m, 1H, CH_2), 3.80-3.83 (m, 3H, OCH_3), 6.87 (d, 1H, J_{HH} = 8.9 Hz, CH), 6.93 (d, 1H, J_{HH} = 8.9 Hz, CH), 7.13 (d, 1H, J_{HH} = 8.0 Hz, CH), 7.21 (d, 1H, J_{HH} = 8.0 Hz, CH), 7.24-7.26 (m, 1H, CH), 7.28-7.32 (m, 2H, CH), 7.40 (d, 1H, J_{HH} = 8.9 Hz, CH); ^{13}C NMR (100 MHz, $CDCl_3$, 296K): δ (ppm) 21.30 (CH_3), 31.26 (CH_2), 43.08 (CH), 55.34 (OCH_3), 114.73, 114.89, 129.85, 130.02, 131.11, 134.22, 134.38, 136.04, 159.44, 160.73, 196.71 (CO); MS (ESI+): m/z 341 $[M+Na]^+$.

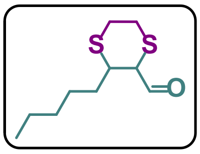 **Product P36**	Colourless oil, isolated yield: 86%; ^{1}H NMR (400 MHz, $CDCl_3$, 296 K): δ (ppm) 0.88 (t, 3H, J_{HH} = 6.9 Hz, C*H*$_3$), 1.27-1.33 (m, 4H, C*H*$_2$), 1.44-1.52 (m, 2H, C*H*$_2$), 1.92-2.00 (m, 2H, C*H*$_2$), 2.72-2.86 (m, 1H, C*H*), 2.91-2.92 (m, 1H, C*H*), 3.29-3.39 (m, 4H, C*H*$_2$), 9.79 (t, 1H, J_{HH} = 2.4 Hz, C*H*); ^{13}C NMR (100 MHz, $CDCl_3$, 296K): δ (ppm) 13.93 (*C*H$_3$), 22.45 (*C*H$_2$), 22.50 (*C*H$_2$), 26.37 (*C*H$_2$), 31.65 (*C*H$_2$), 40.18 (S*C*H$_2$), 45.31 (S*C*H$_2$), 54.36 (*C*H), 65.99 (*C*H), 200.98 (*C*O); IR: ν (cm^{-1}): 1688 (C=O);MS (ESI+): m/z 273 [M+2O+K]$^{+}$.

2. NMR spectra of compounds

2.1. NMR spectra of ***NHC-1***

NHC-1

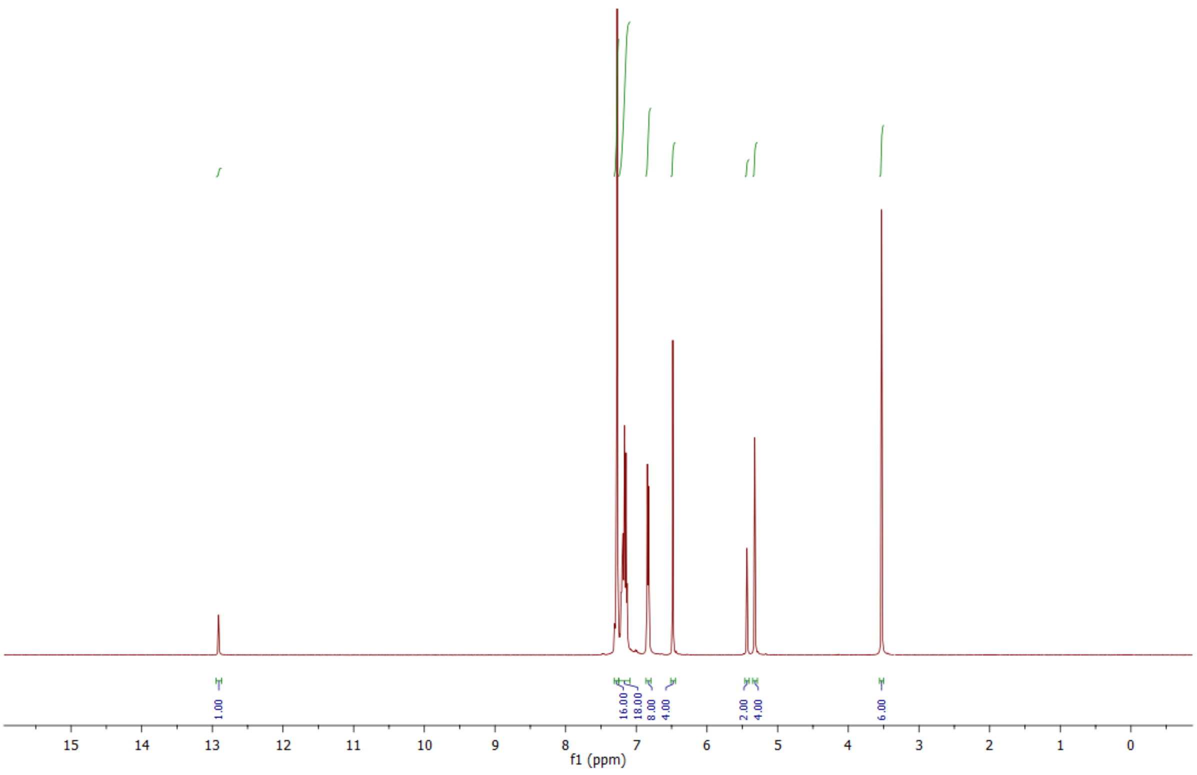

Figure S1. ^{1}H NMR (400 MHz, $CDCl_3$) of product **NHC-1**

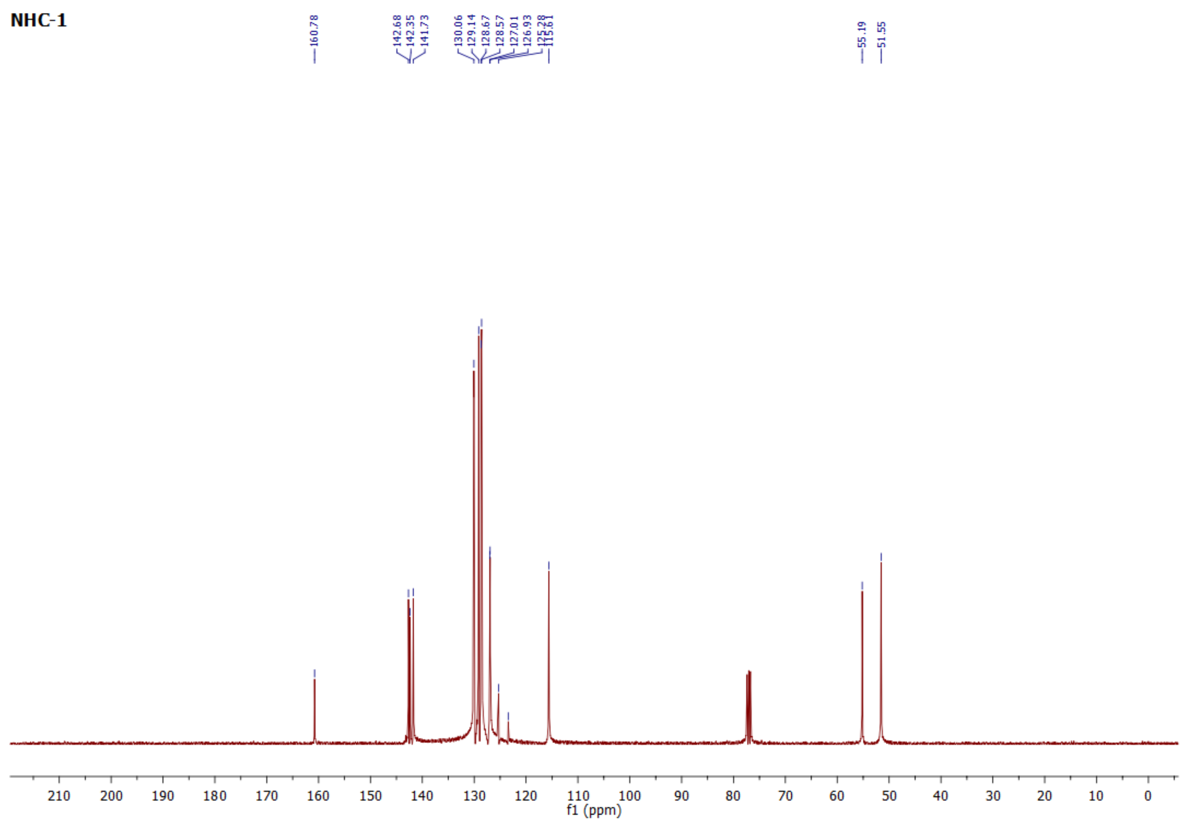

Figure S2. ^{13}C NMR (101 MHz, $CDCl_3$) of product **NHC-1**

*2.2. NMR spectra of isolated products **P1-P36***

Product P1

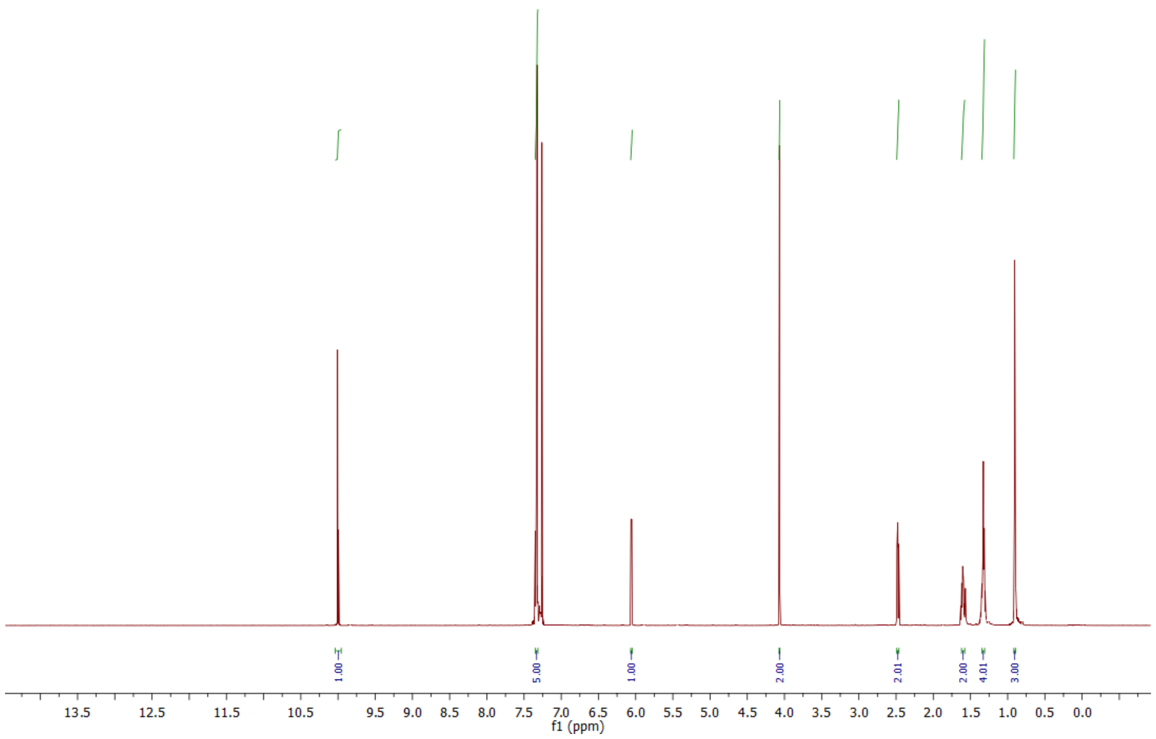

Figure S3. ^{1}H NMR (400 MHz, $CDCl_3$) of product **P1**

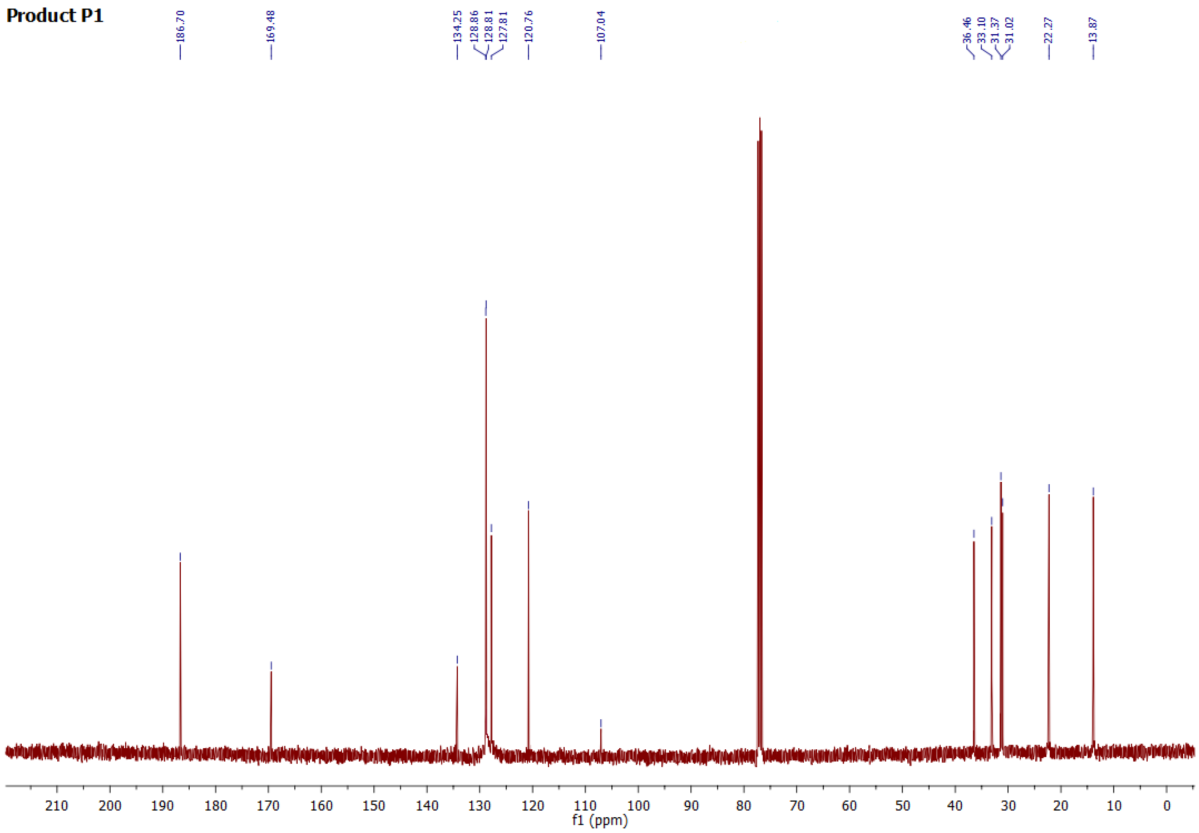

Figure S4. ^{13}C NMR (101 MHz, $CDCl_3$) of product **P1**

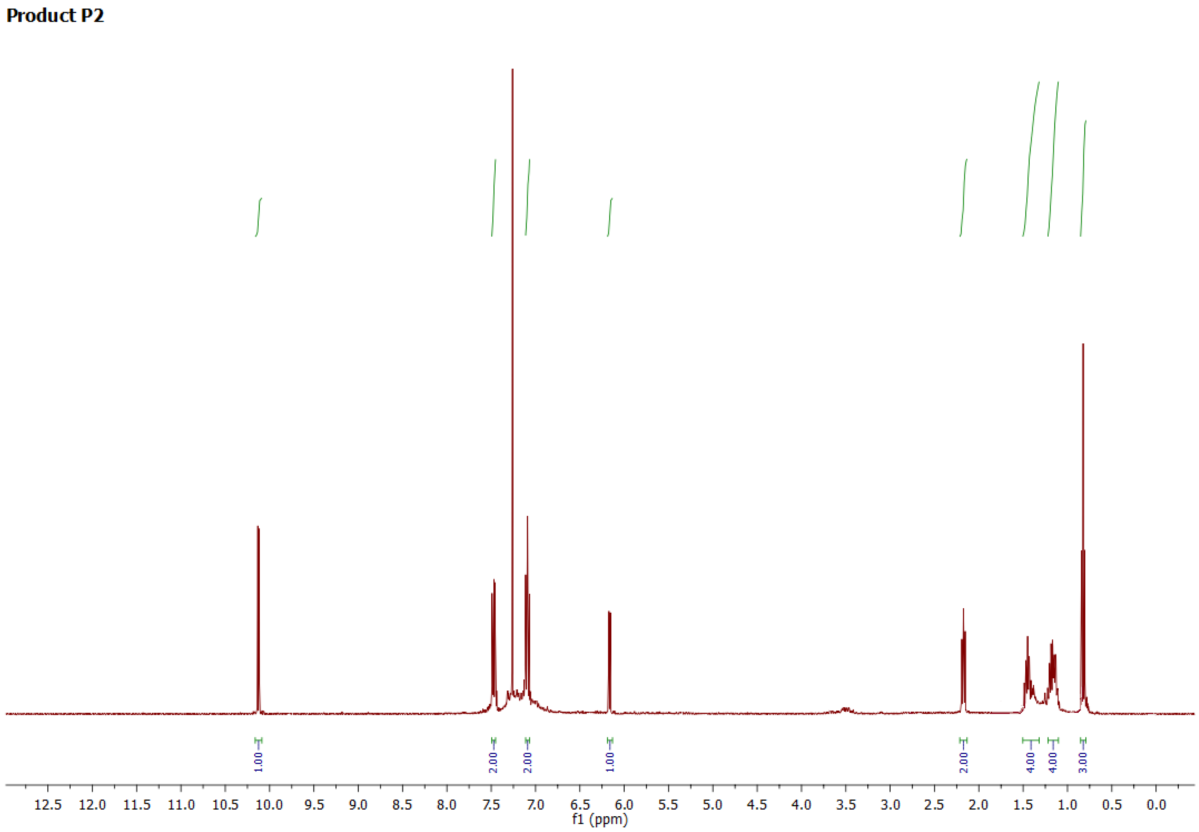

Figure S5. ^{1}H NMR (400 MHz, $CDCl_3$) of product **P2**

Product P2

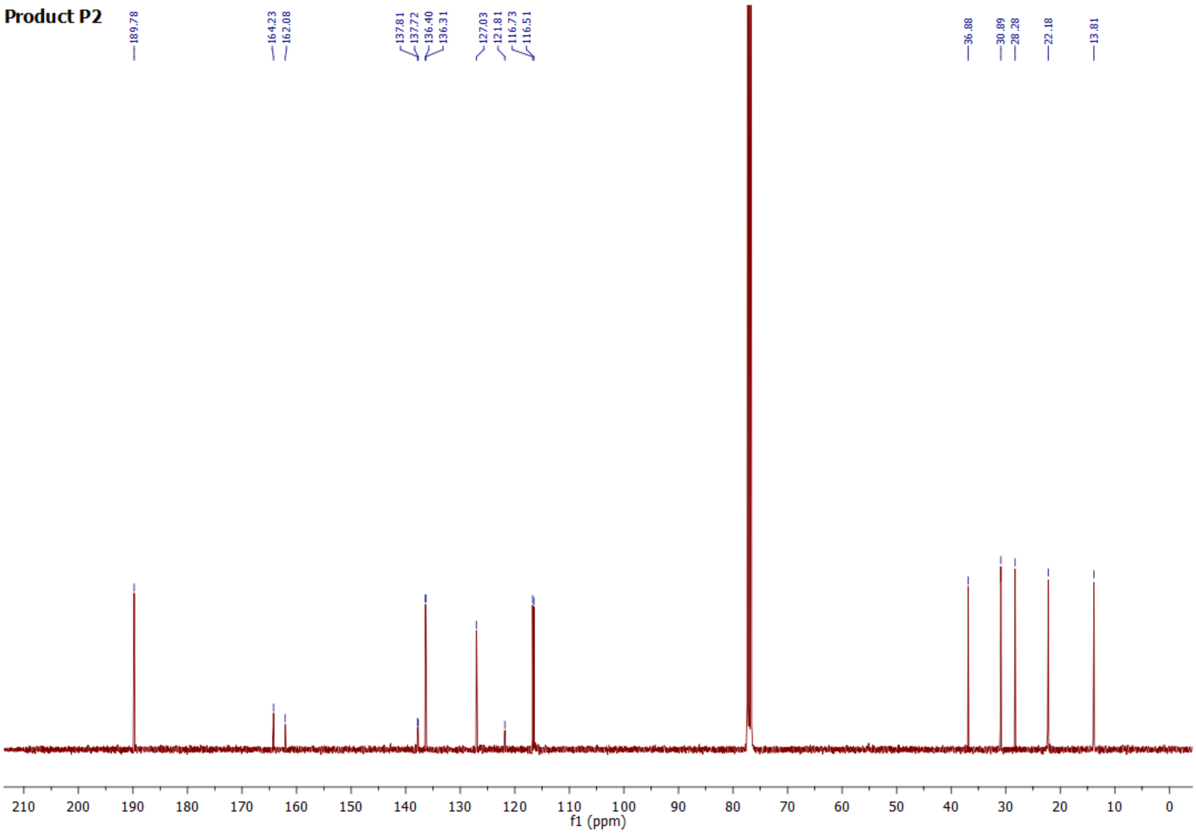

Figure S6. ^{13}C NMR (101 MHz, $CDCl_3$) of product **P2**

Product P3

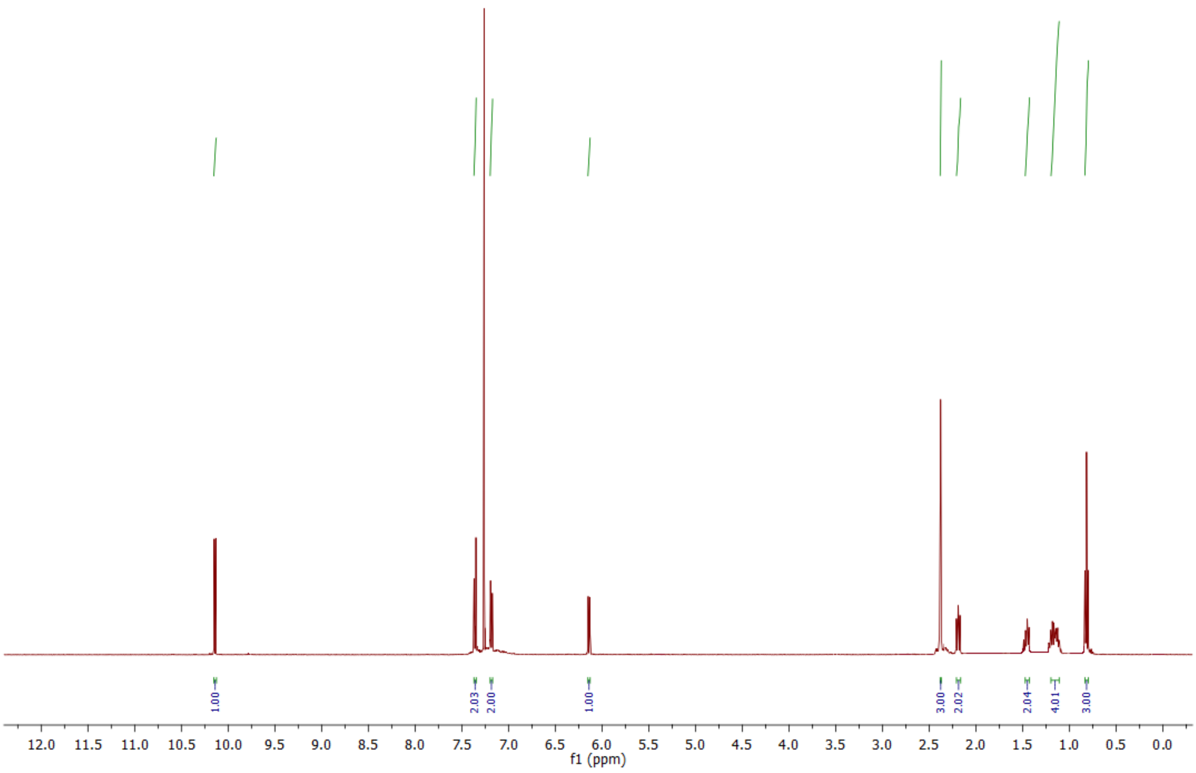

Figure S7. ^{1}H NMR (400 MHz, $CDCl_3$) of product **P3**

Product P3

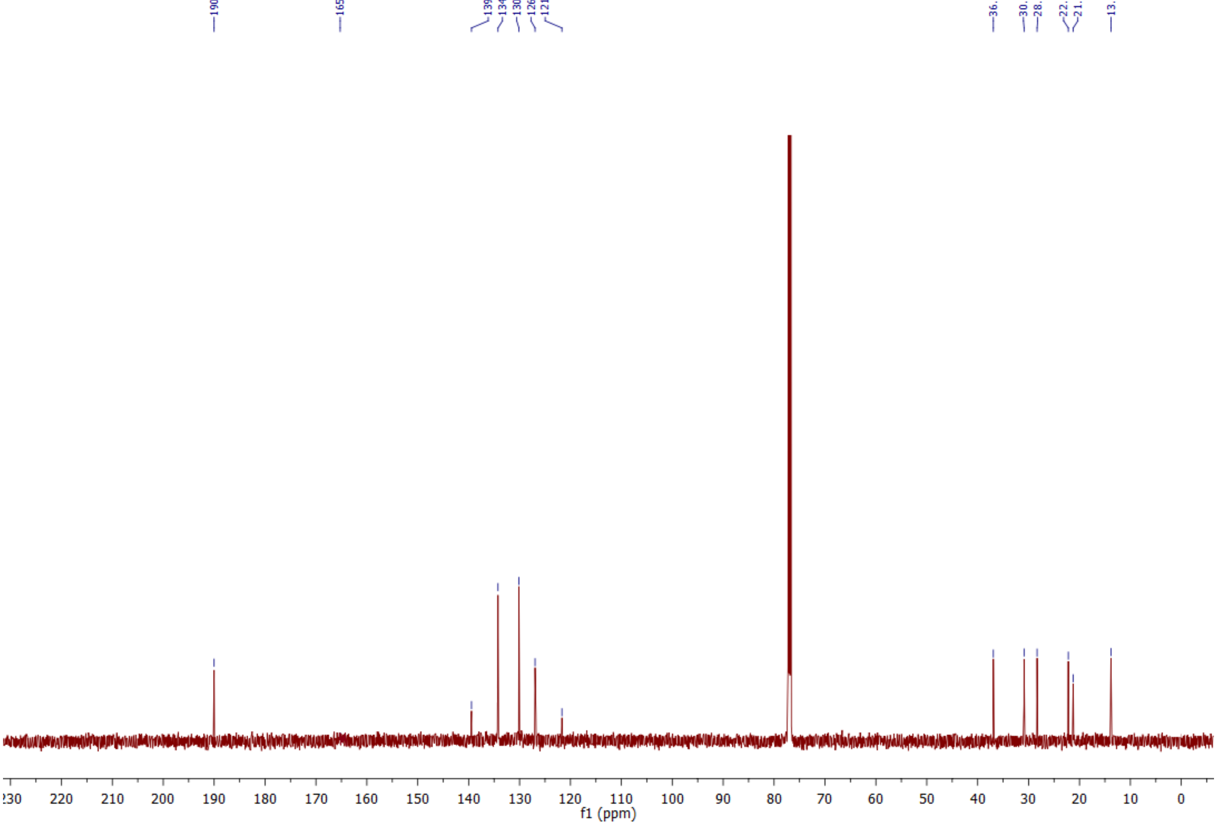

Figure S8. ^{13}C NMR (101 MHz, $CDCl_3$) of product **P3**

Product P4

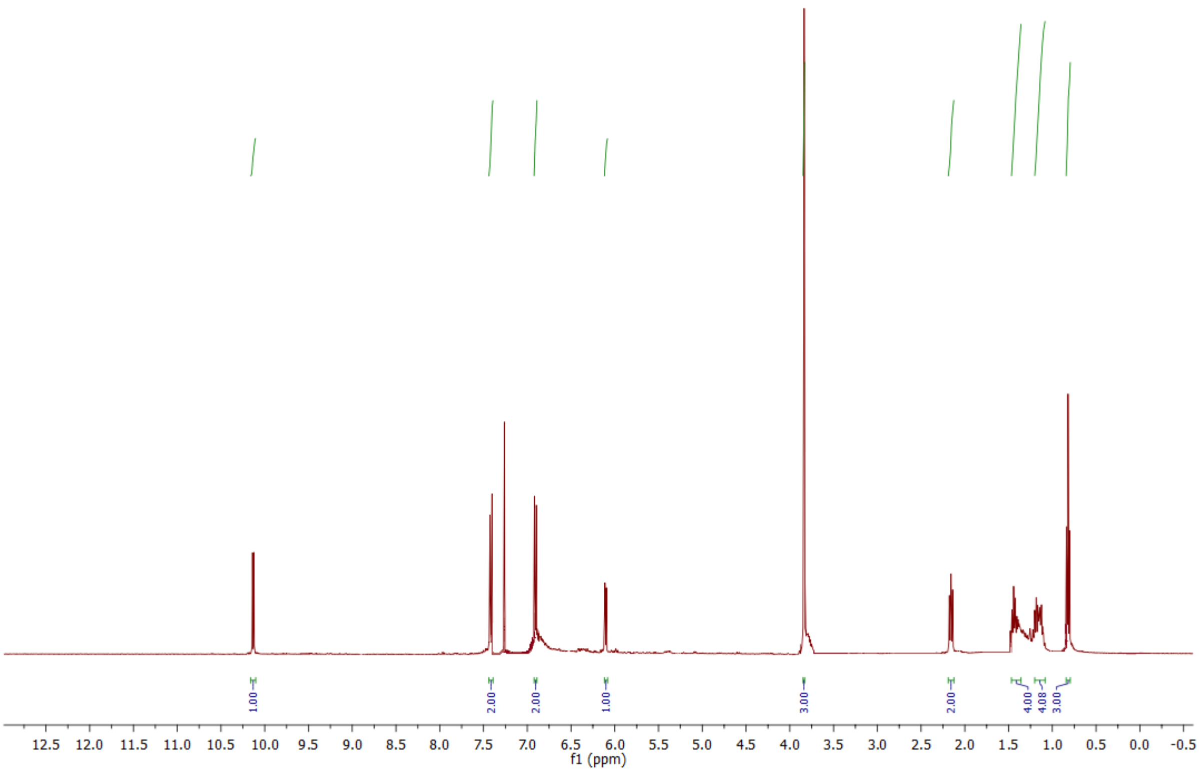

Figure S9. ^{1}H NMR (400 MHz, $CDCl_3$) of product **P4**

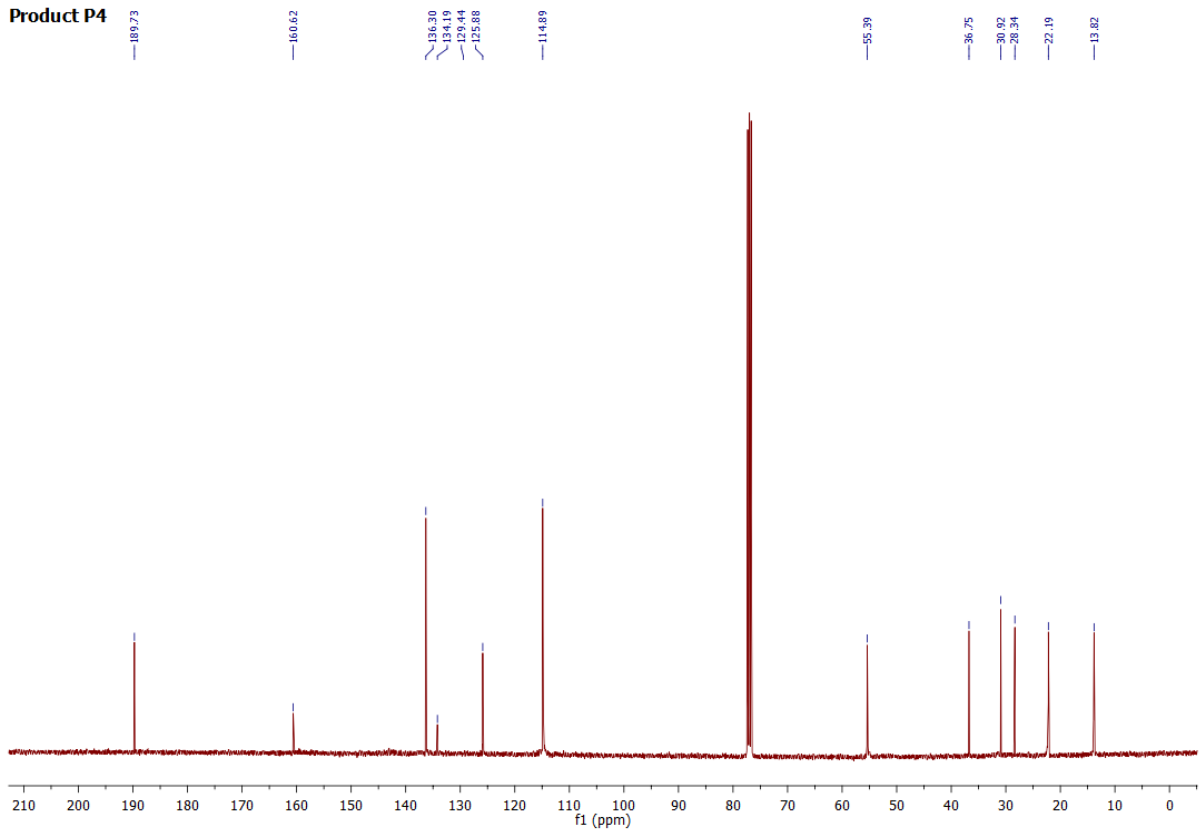

Figure S10. ^{13}C NMR (101 MHz, $CDCl_3$) of product **P4**

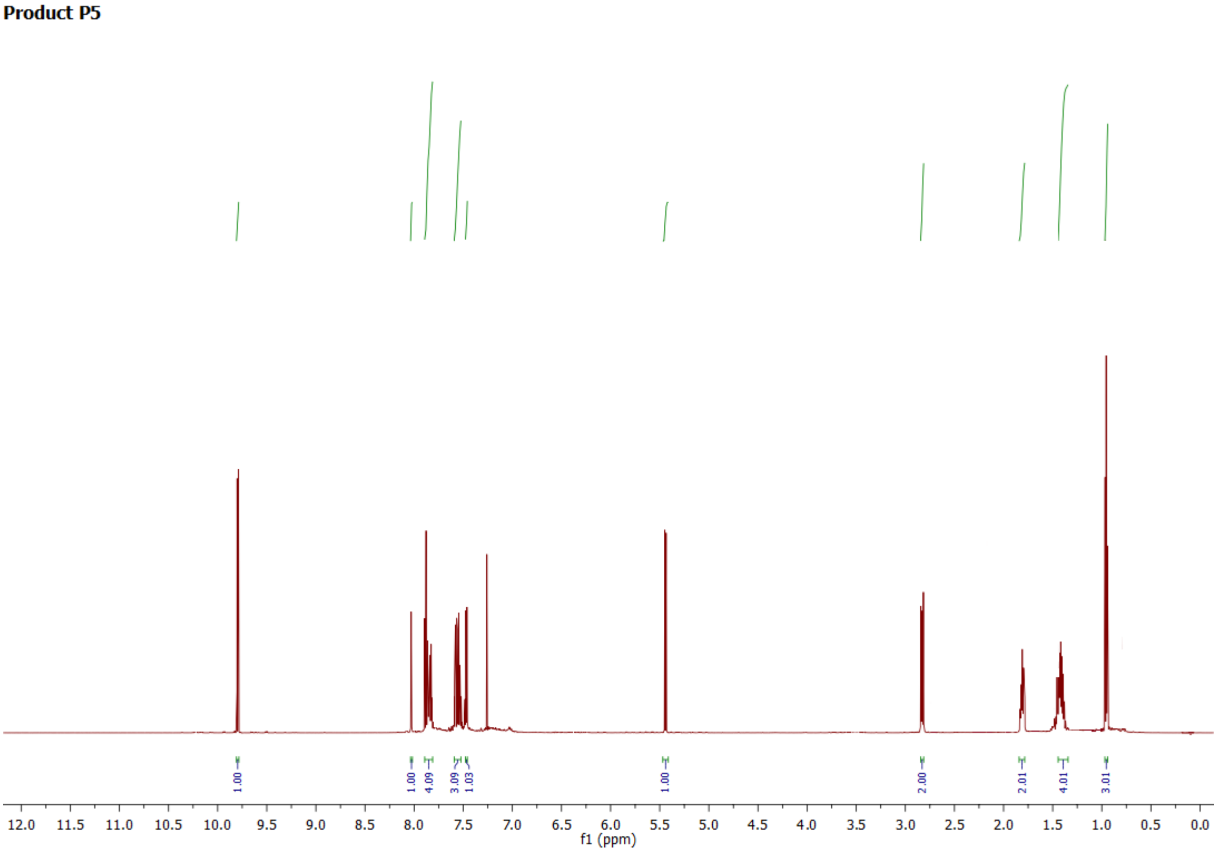

Figure S11. ^{1}H NMR (400 MHz, $CDCl_3$) of product **P5**

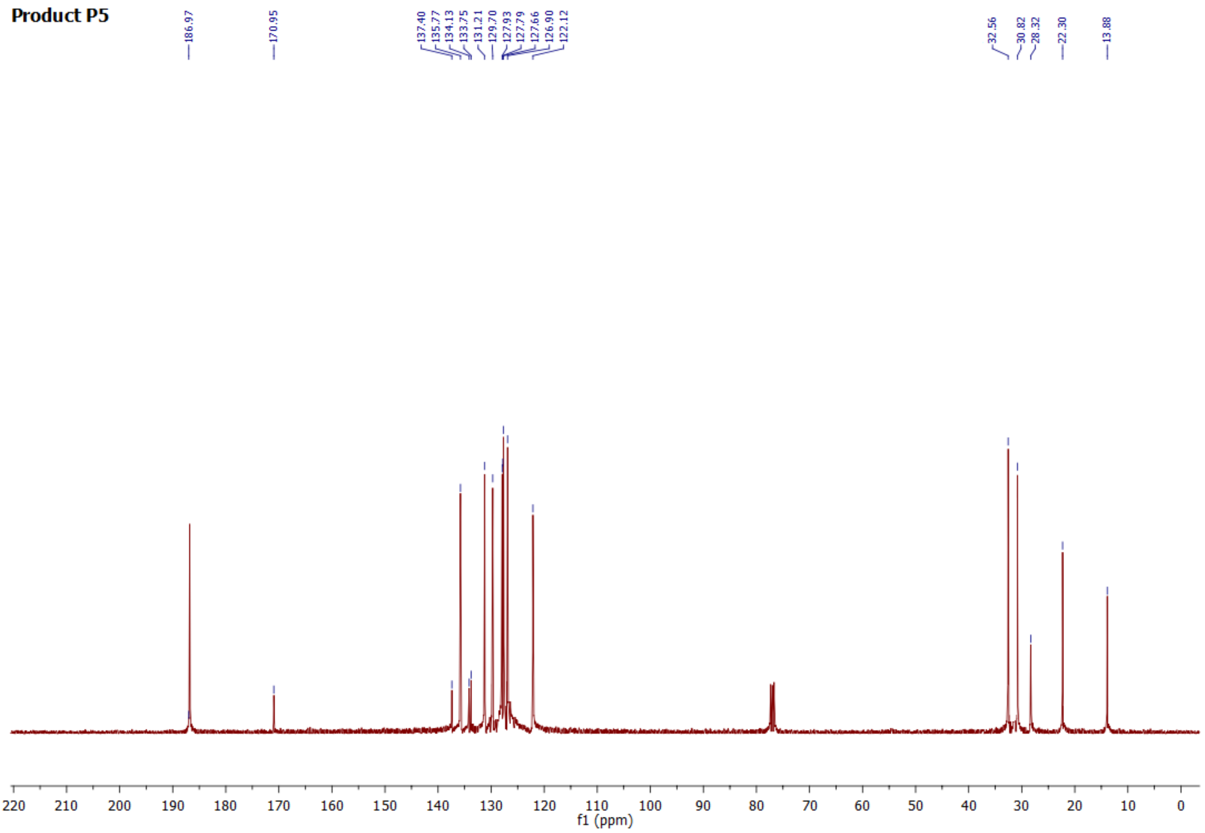

Figure S12. ^{13}C NMR (101 MHz, $CDCl_3$) of product **P5**

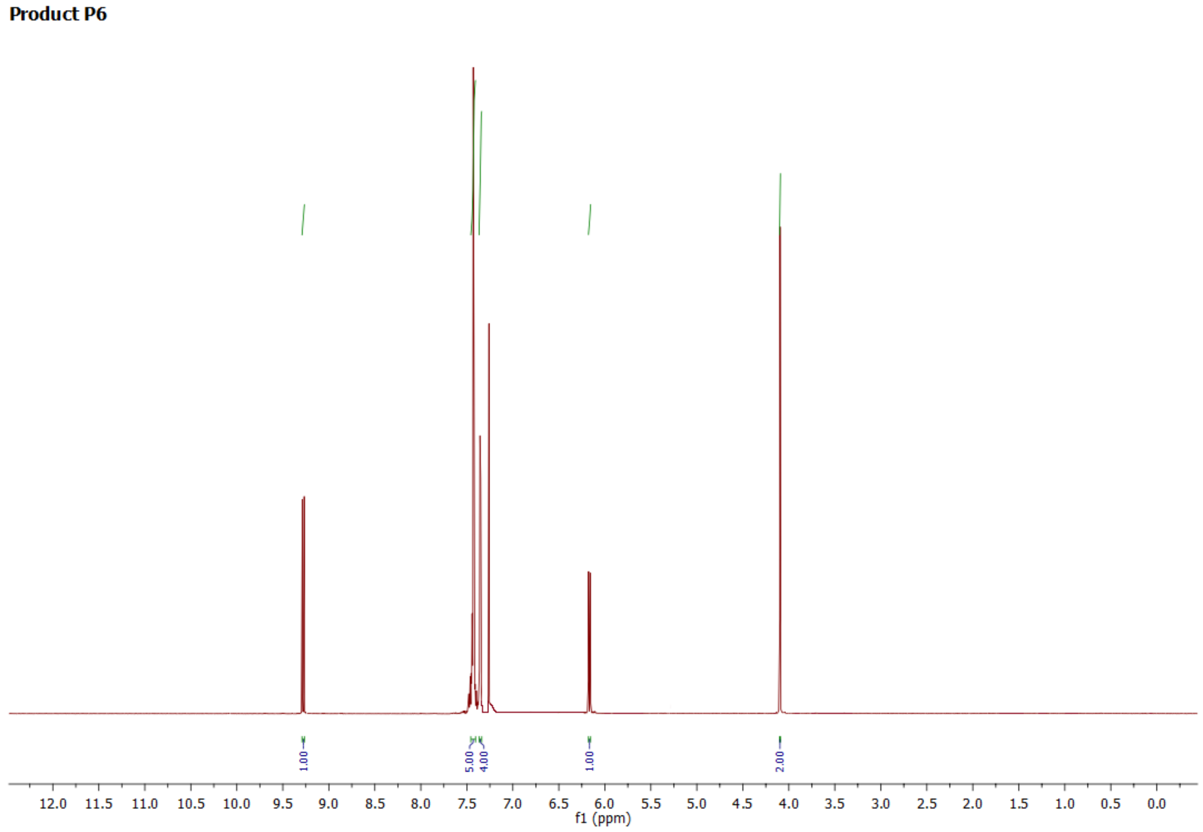

Figure S13. ^{1}H NMR (400 MHz, $CDCl_3$) of product **P6**

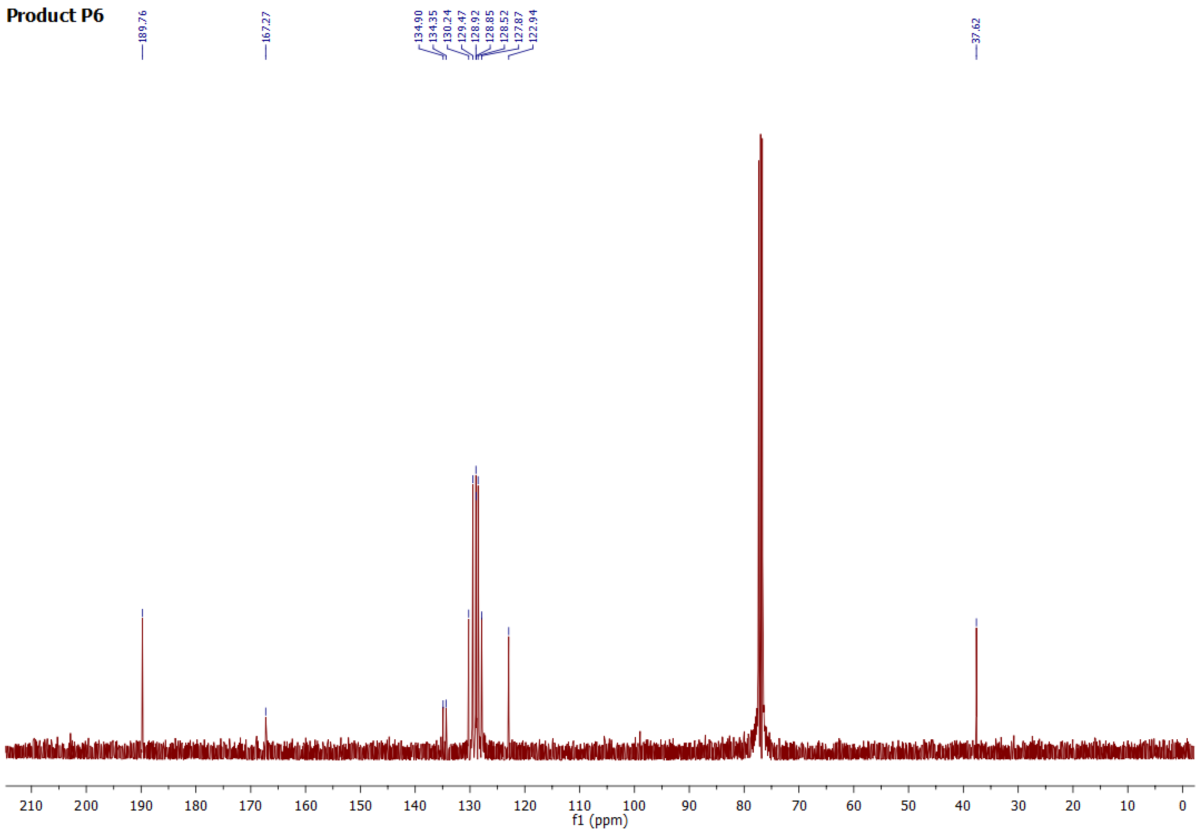

Figure S14. ^{13}C NMR (101 MHz, $CDCl_3$) of product **P6**

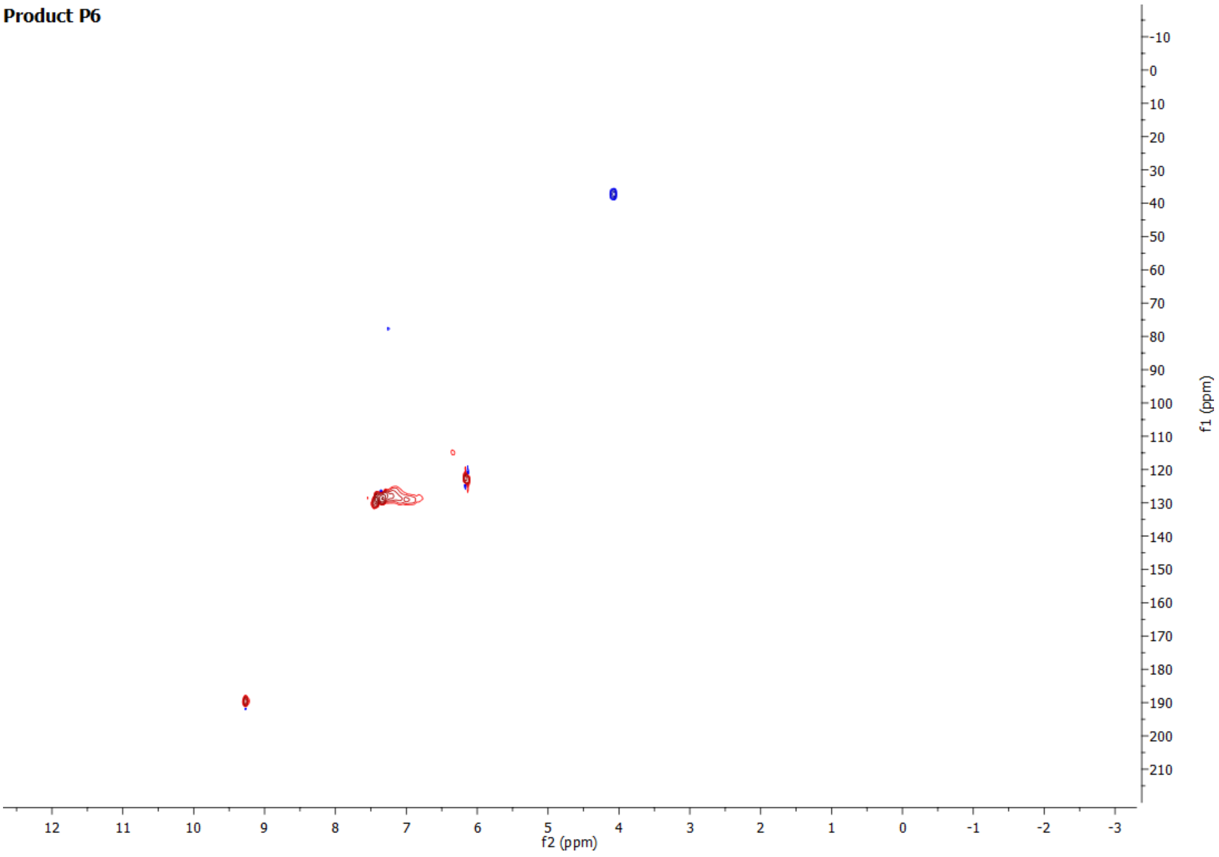

Figure S15. ^{1}H ^{13}C HSQC (600 MHz, $CDCl_3$) of product **P6**

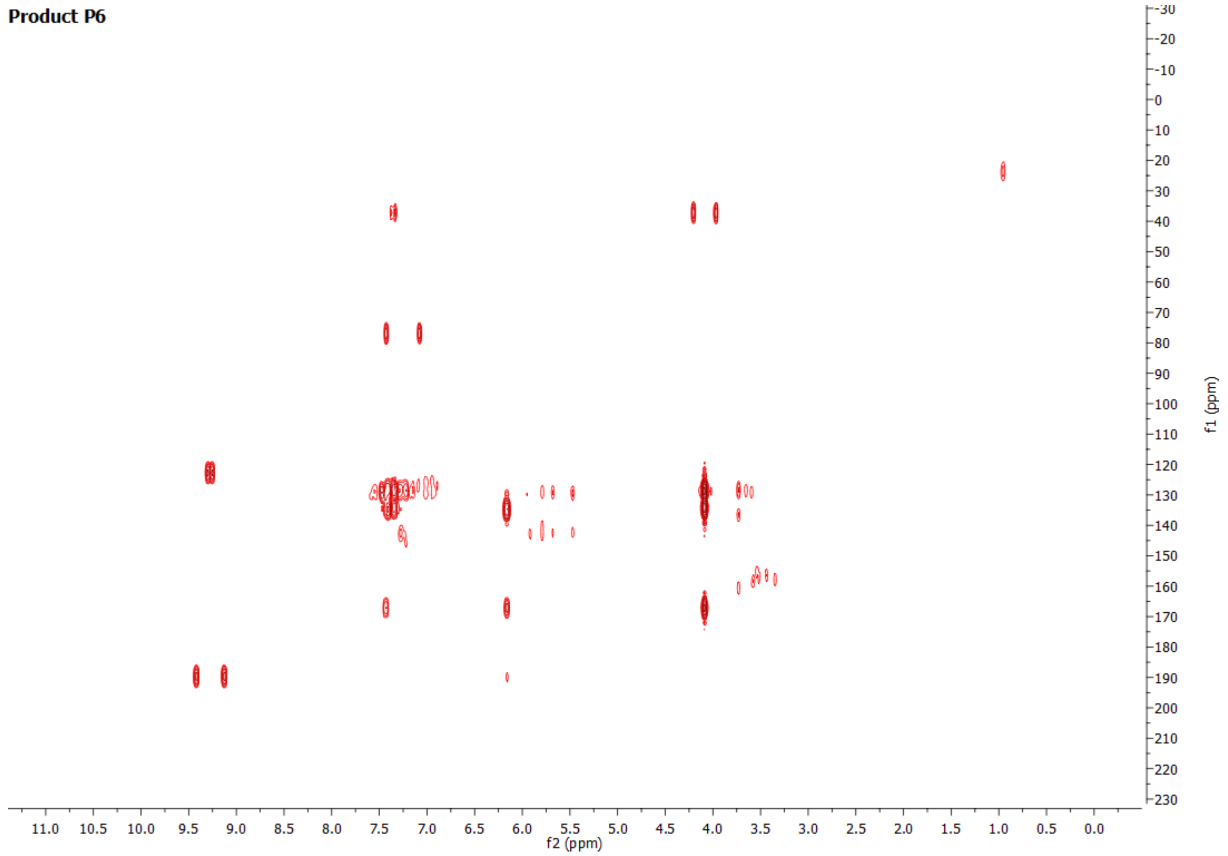

Figure S16. ^{1}H ^{13}C HMBC (600 MHz, $CDCl_3$) of product **P6**

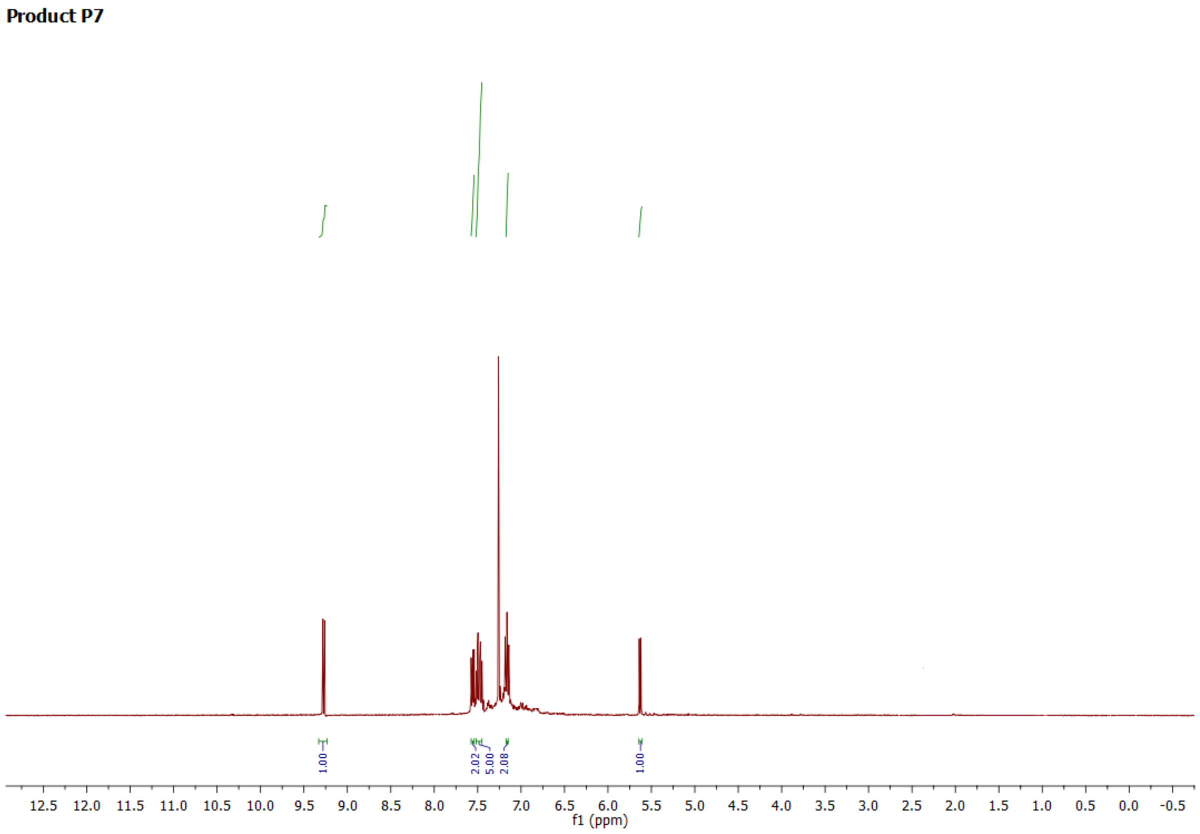

Figure S17. ^{1}H NMR (400 MHz, $CDCl_3$) of product **P7**

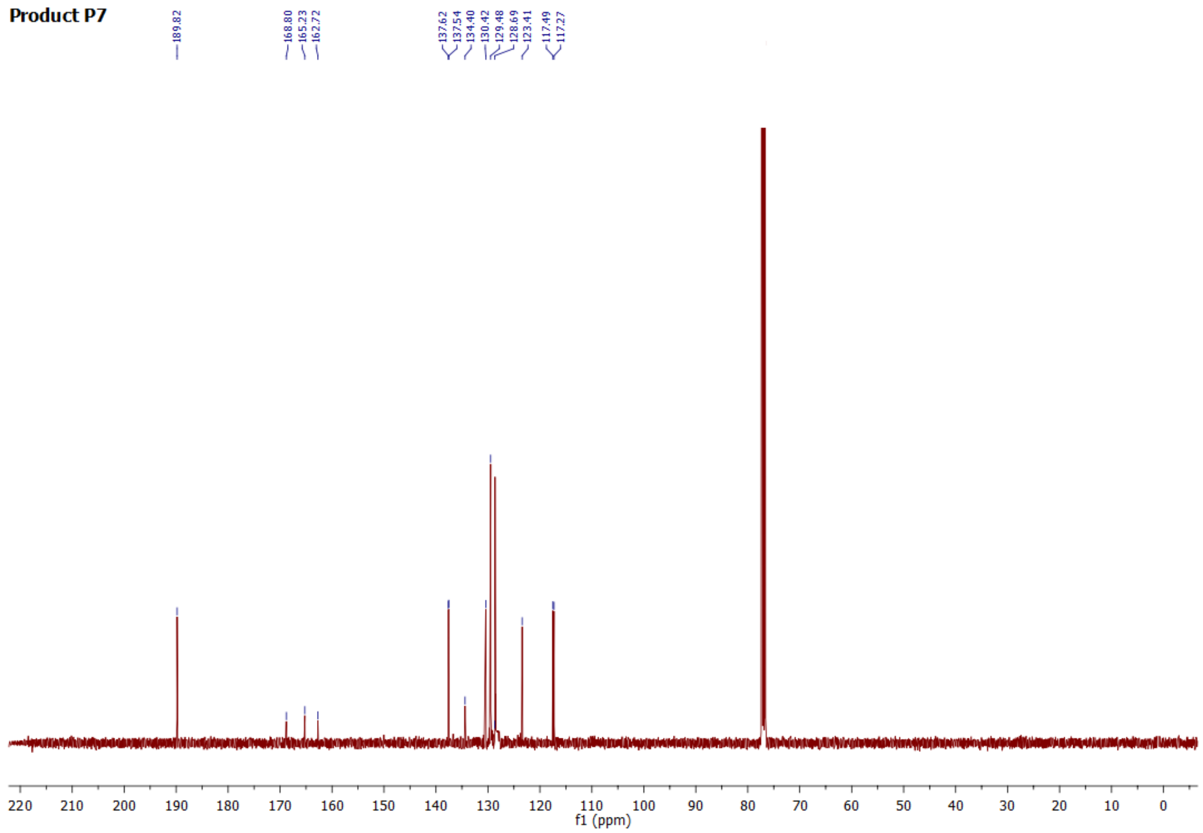

Figure S18. ^{13}C NMR (101 MHz, $CDCl_3$) of product **P7**

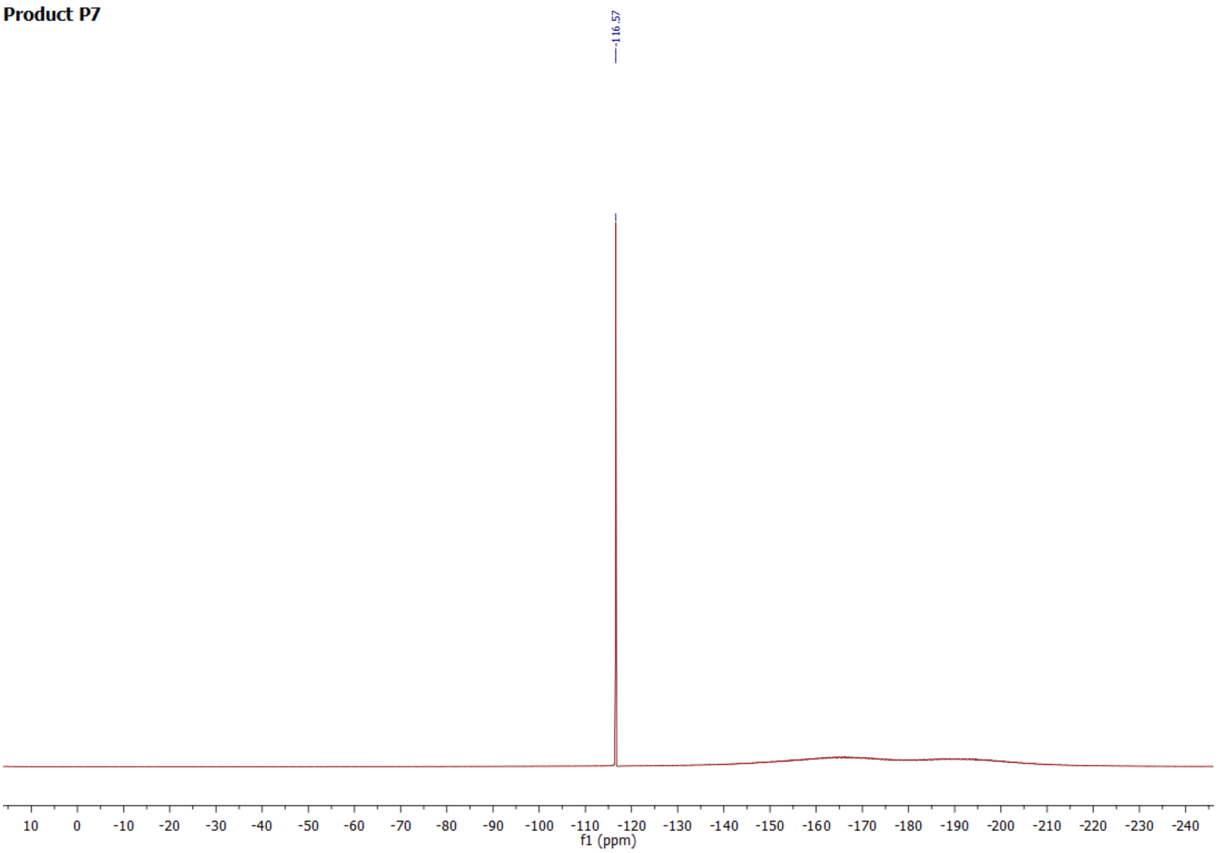

Figure S19. ^{29}F NMR (376 MHz, $CDCl_3$) of product **P7**

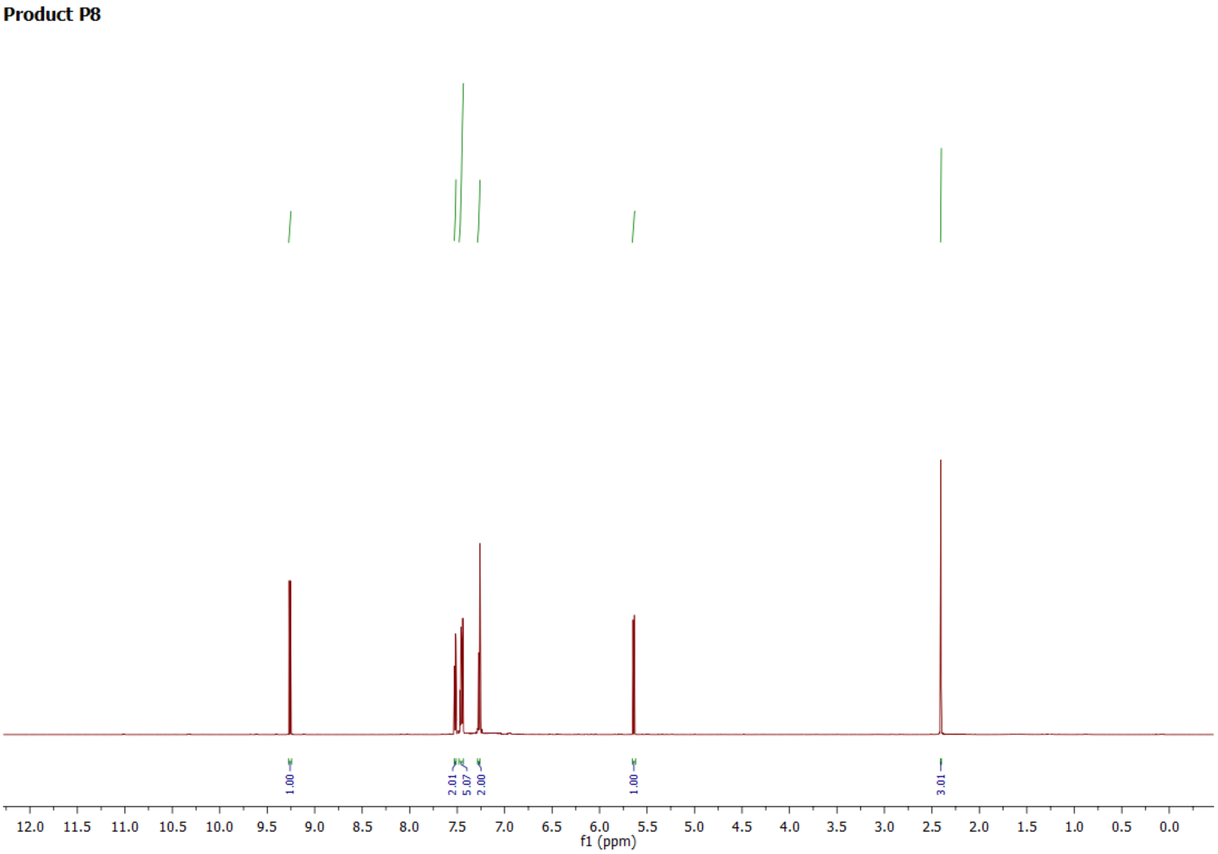

Figure S20. ^{1}H NMR (400 MHz, $CDCl_3$) of product **P8**

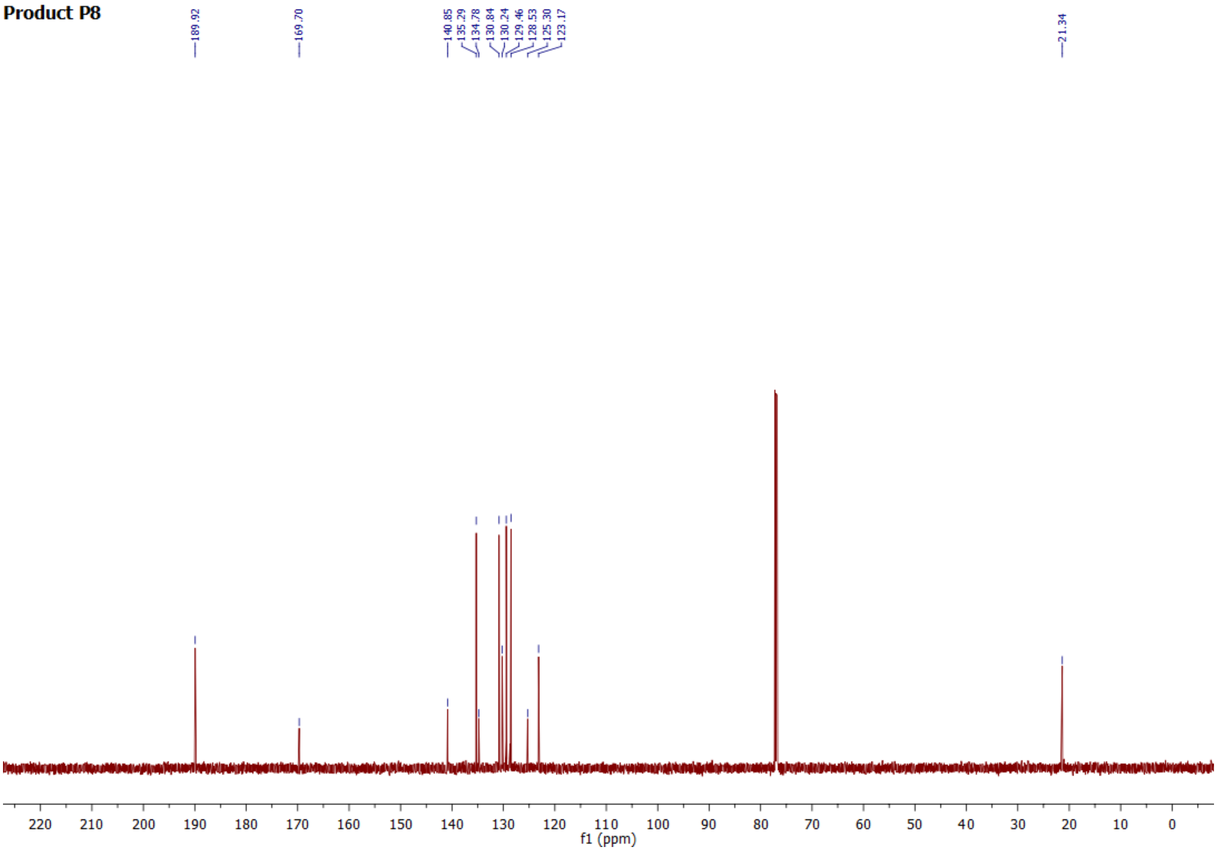

Figure S21. ^{13}C NMR (101 MHz, $CDCl_3$) of product **P8**

Product P9

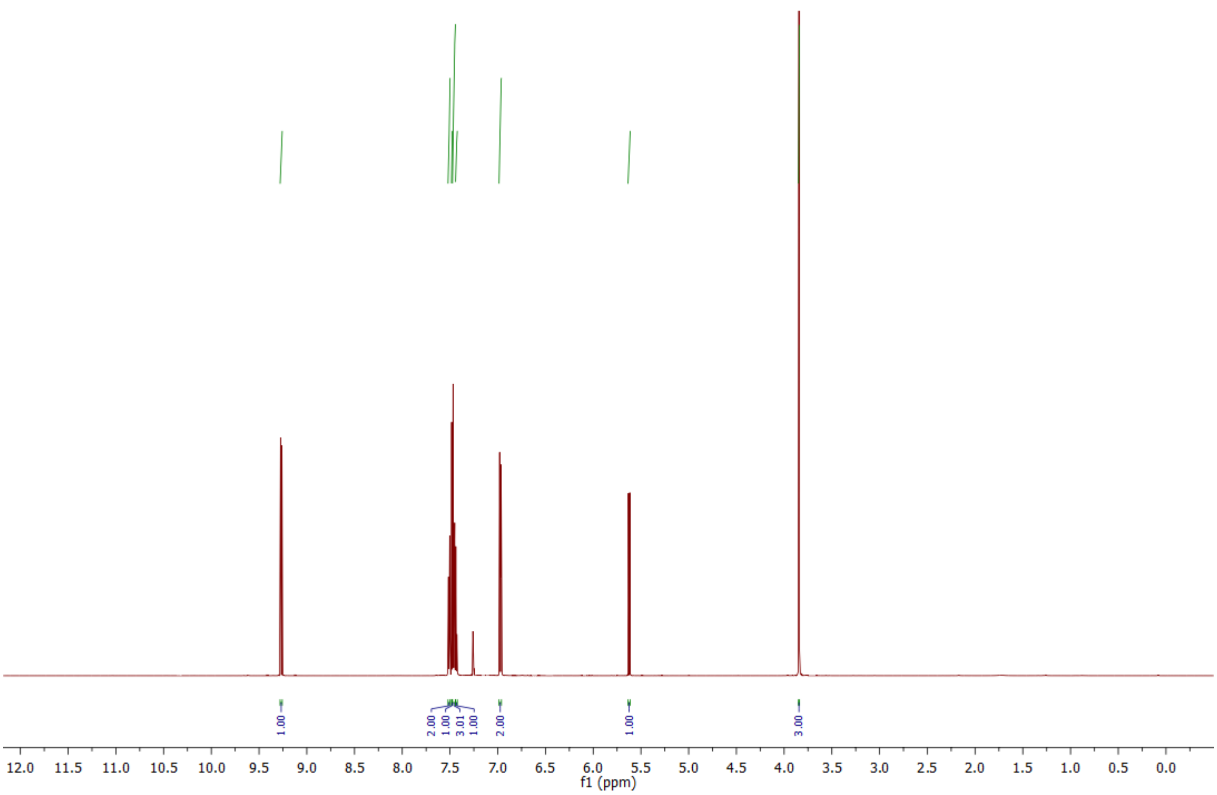

Figure S22. ^{1}H NMR (400 MHz, $CDCl_3$) of product **P9**

Product P9

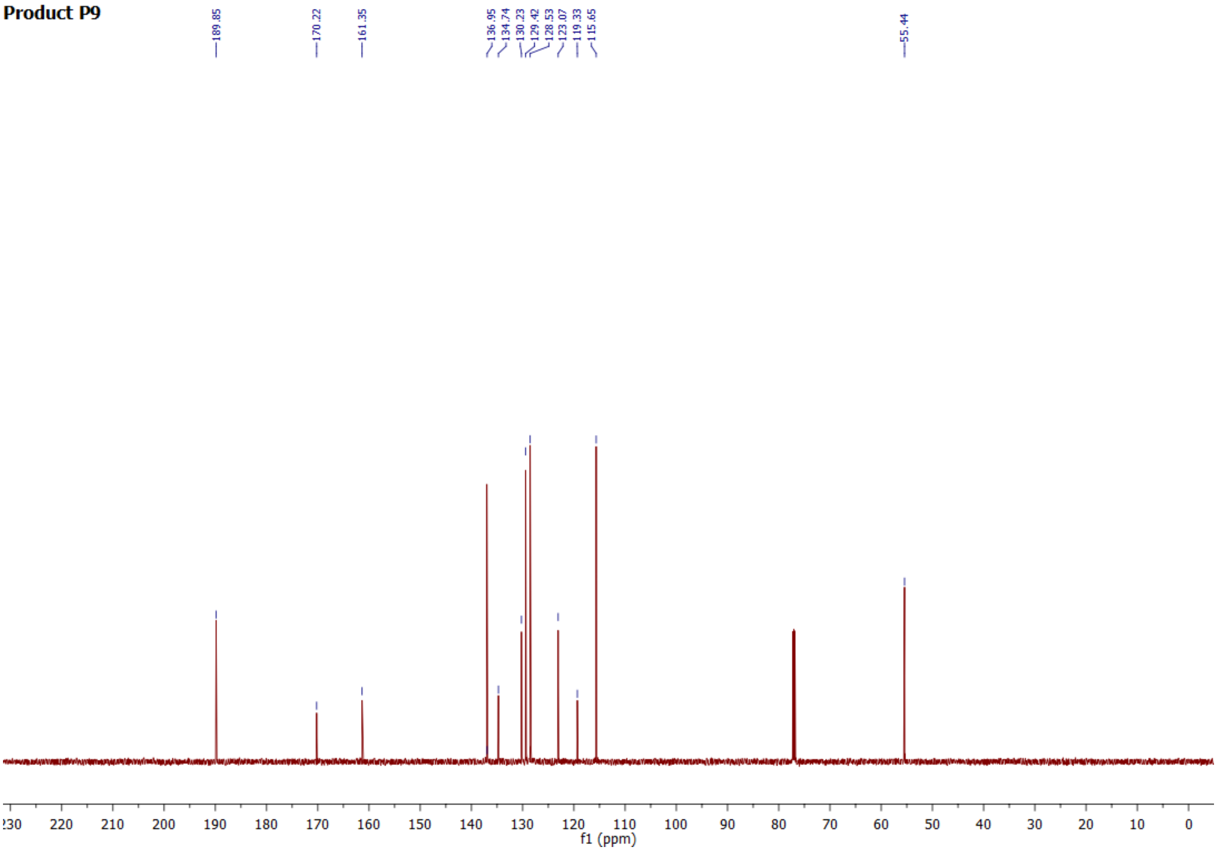

Figure S23. ^{13}C NMR (101 MHz, $CDCl_3$) of product **P9**

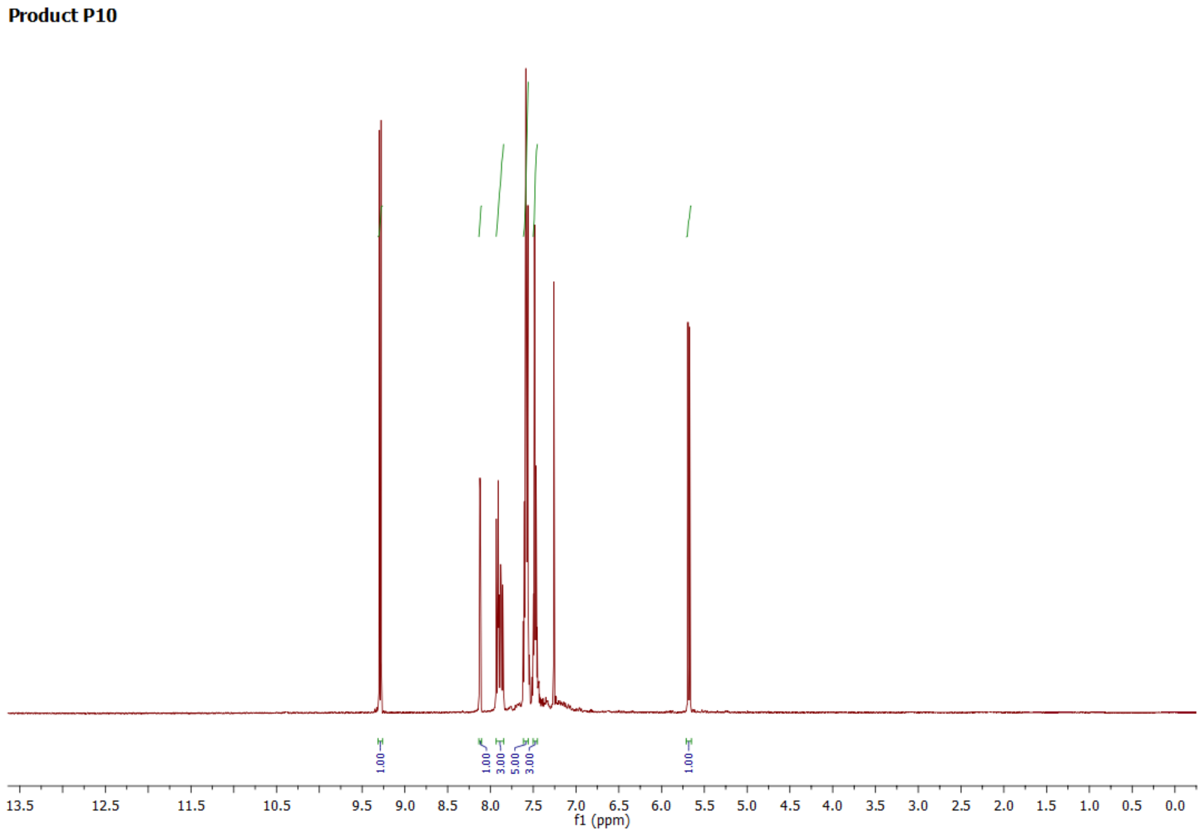

Figure S24. ^{1}H NMR (400 MHz, $CDCl_3$) of product **P10**

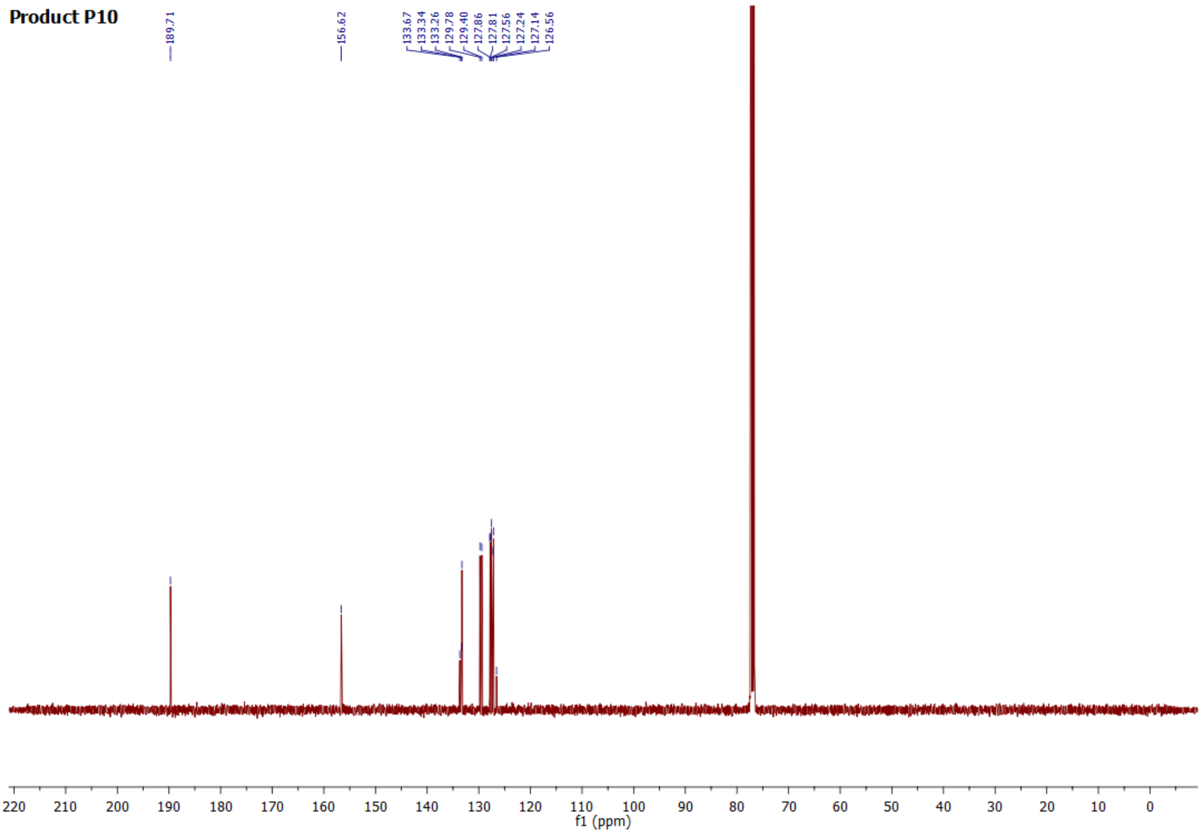

Figure S25. ^{13}C NMR (101 MHz, $CDCl_3$) of product **P10**

Product P11

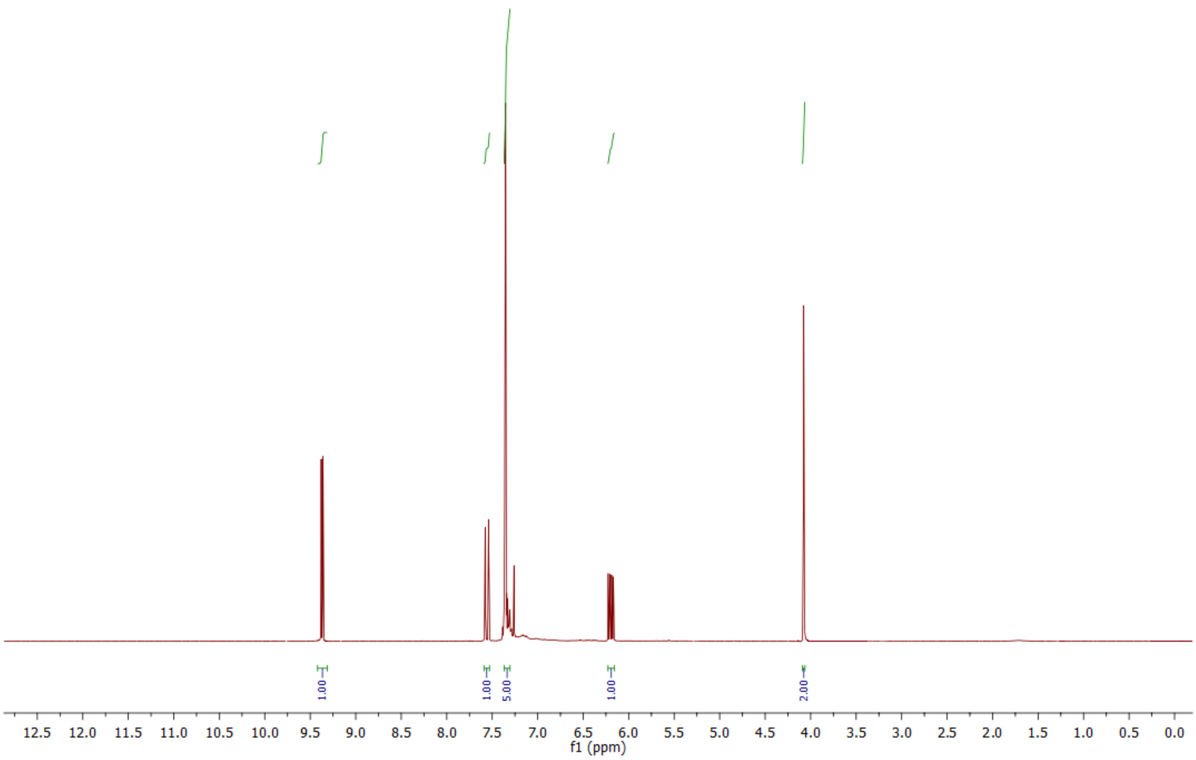

Figure S26. ^{1}H NMR (400 MHz, $CDCl_3$) of product **P11**

Product P11

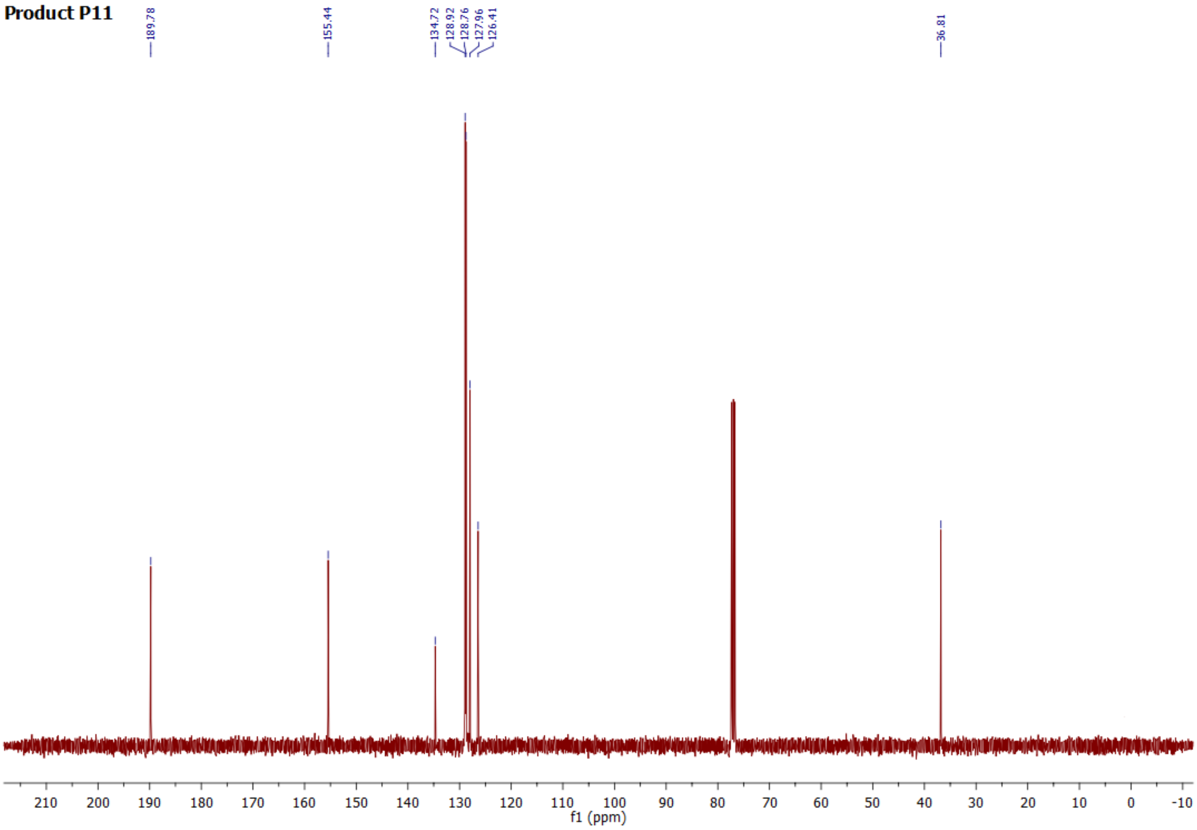

Figure S27. ^{13}C NMR (101 MHz, $CDCl_3$) of product **P11**

Product P12

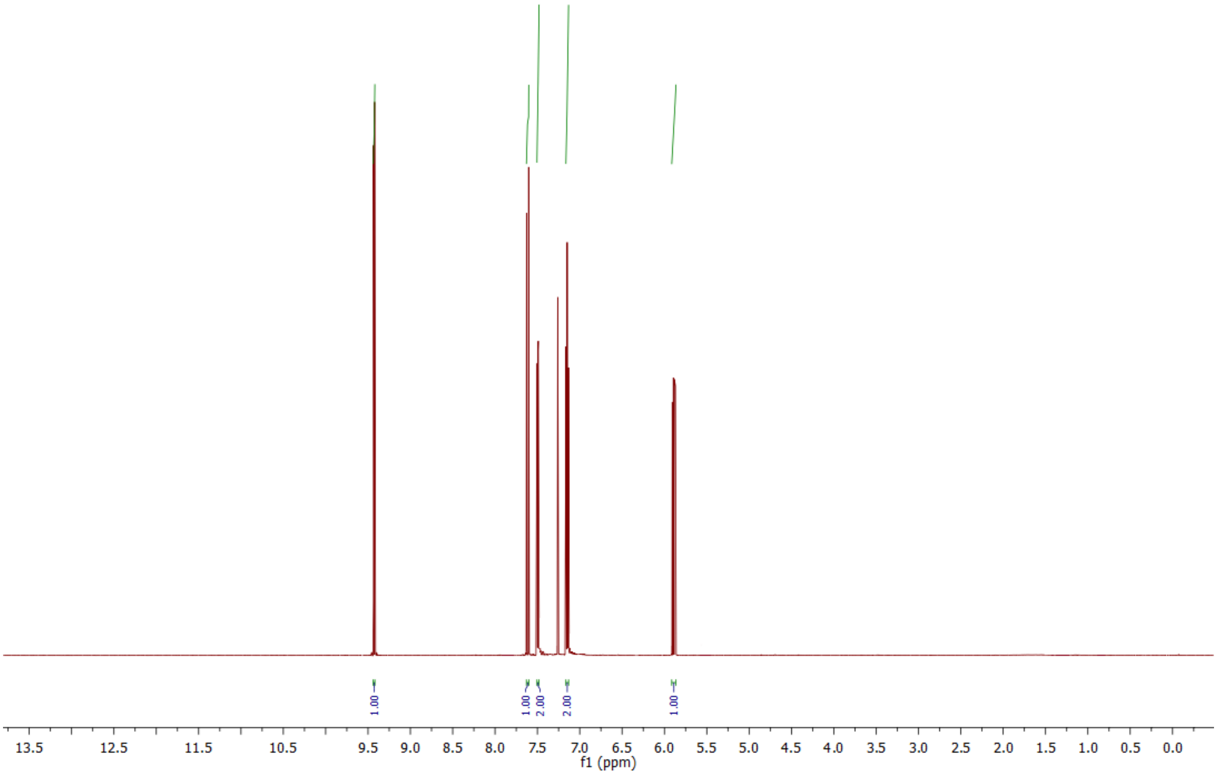

Figure S28. ^{1}H NMR (400 MHz, $CDCl_3$) of product **P12**

Product P12

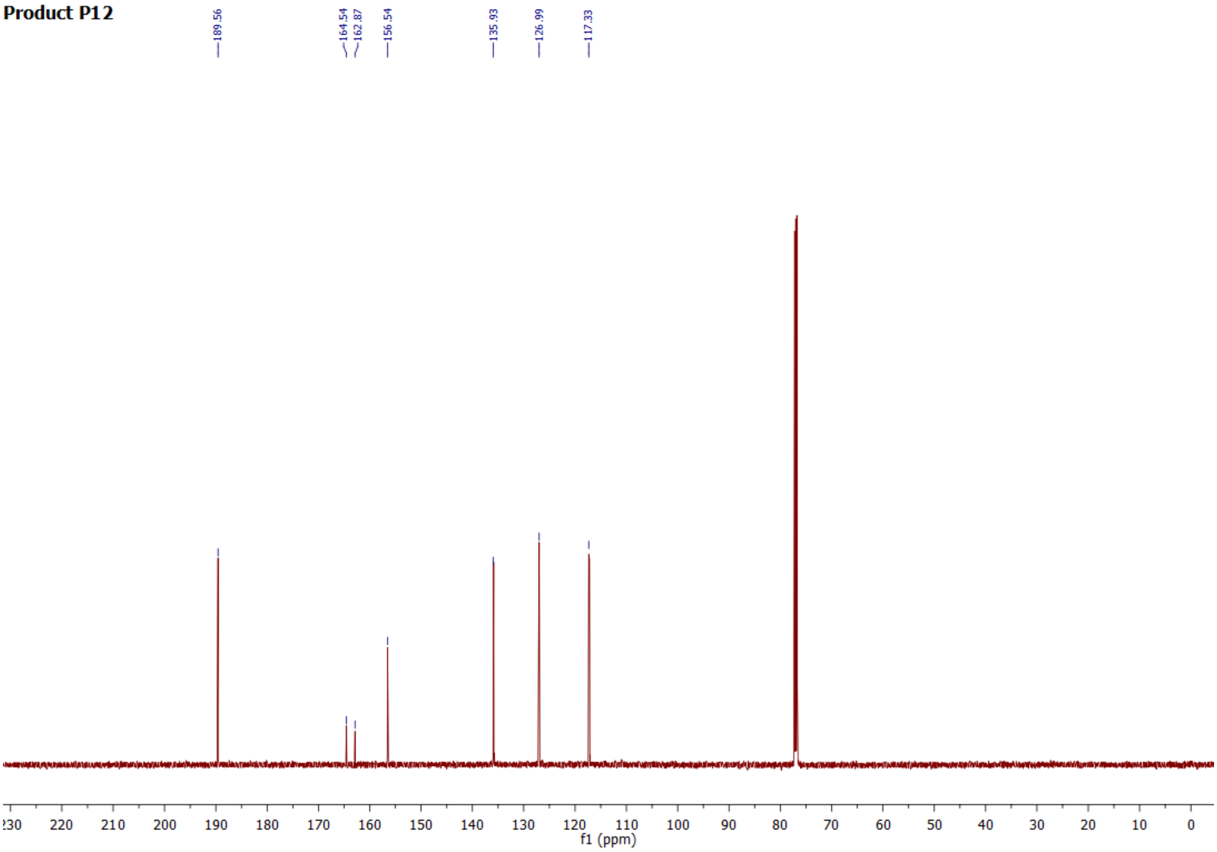

Figure S29. ^{13}C NMR (101 MHz, $CDCl_3$) of product **P12**

Product P13

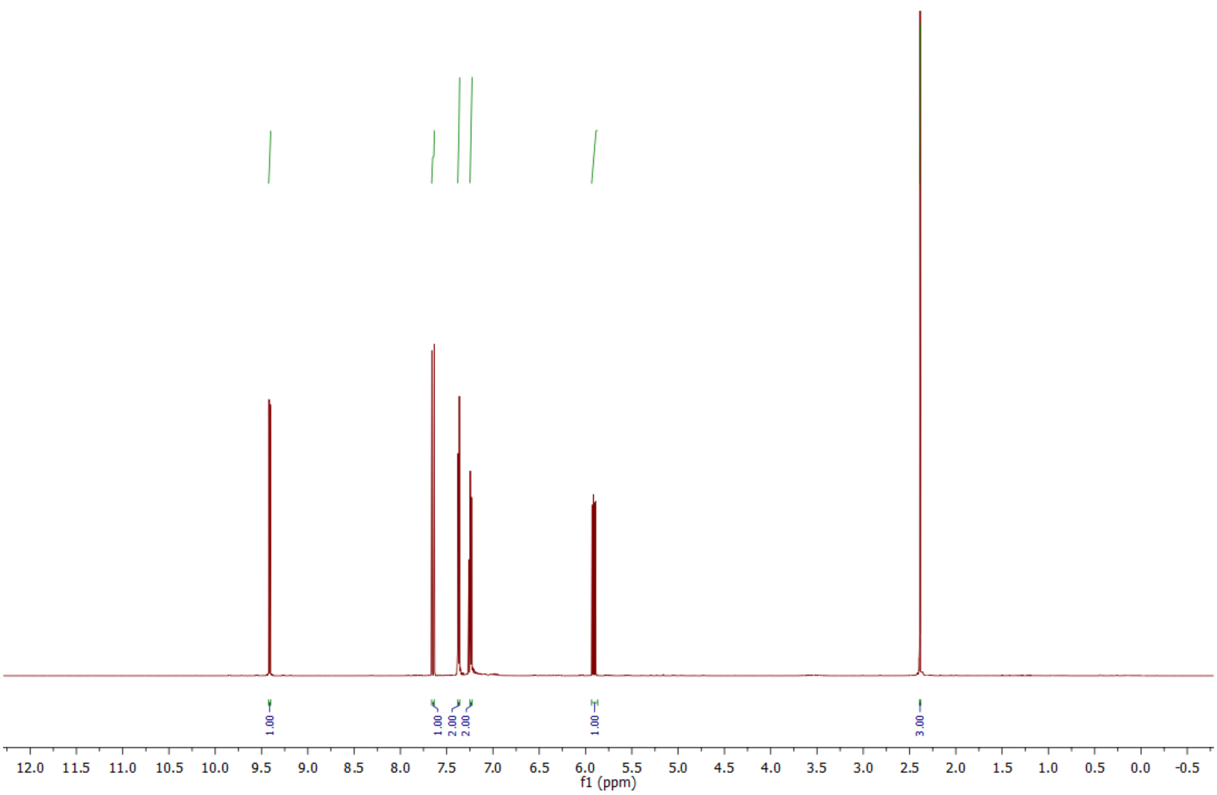

Figure S30. ^{1}H NMR (400 MHz, $CDCl_3$) of product **P13**

Product P13

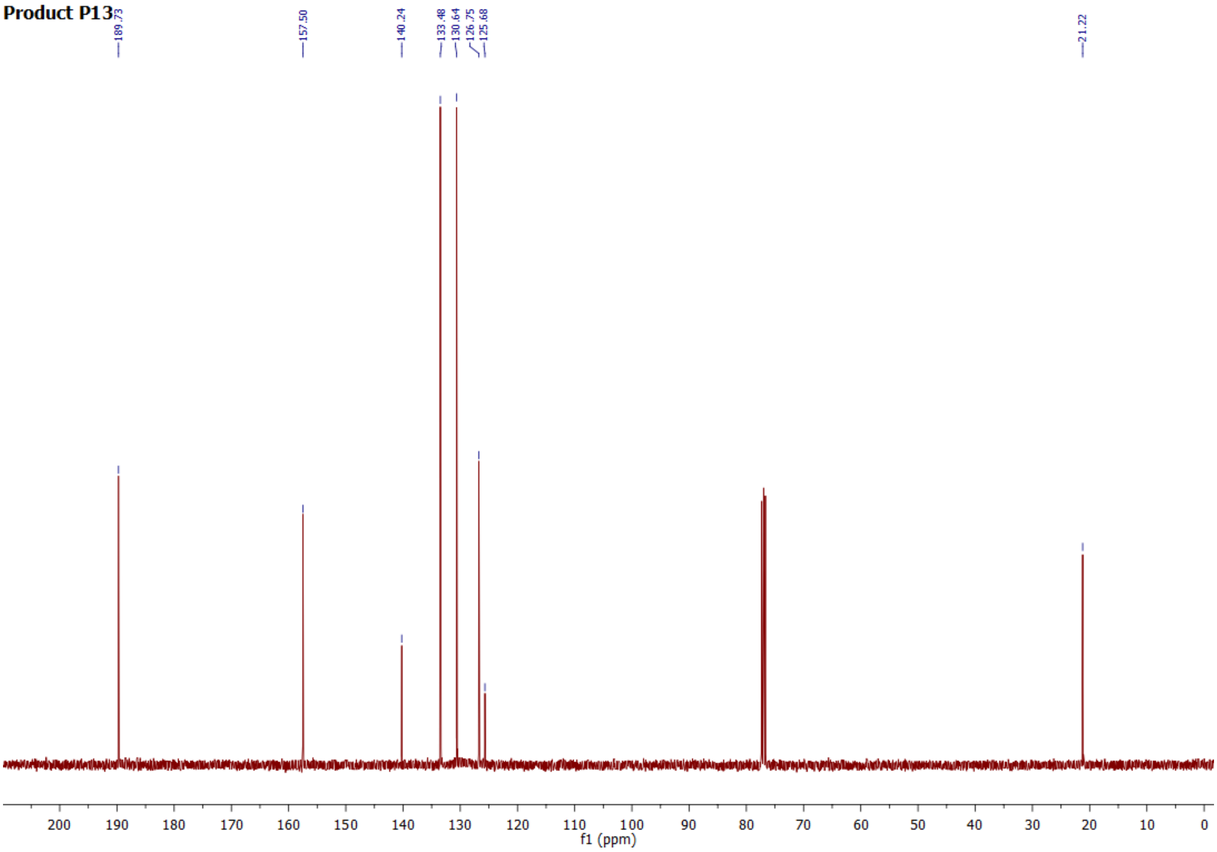

Figure S31. ^{13}C NMR (101 MHz, $CDCl_3$) of product **P13**

Product P14

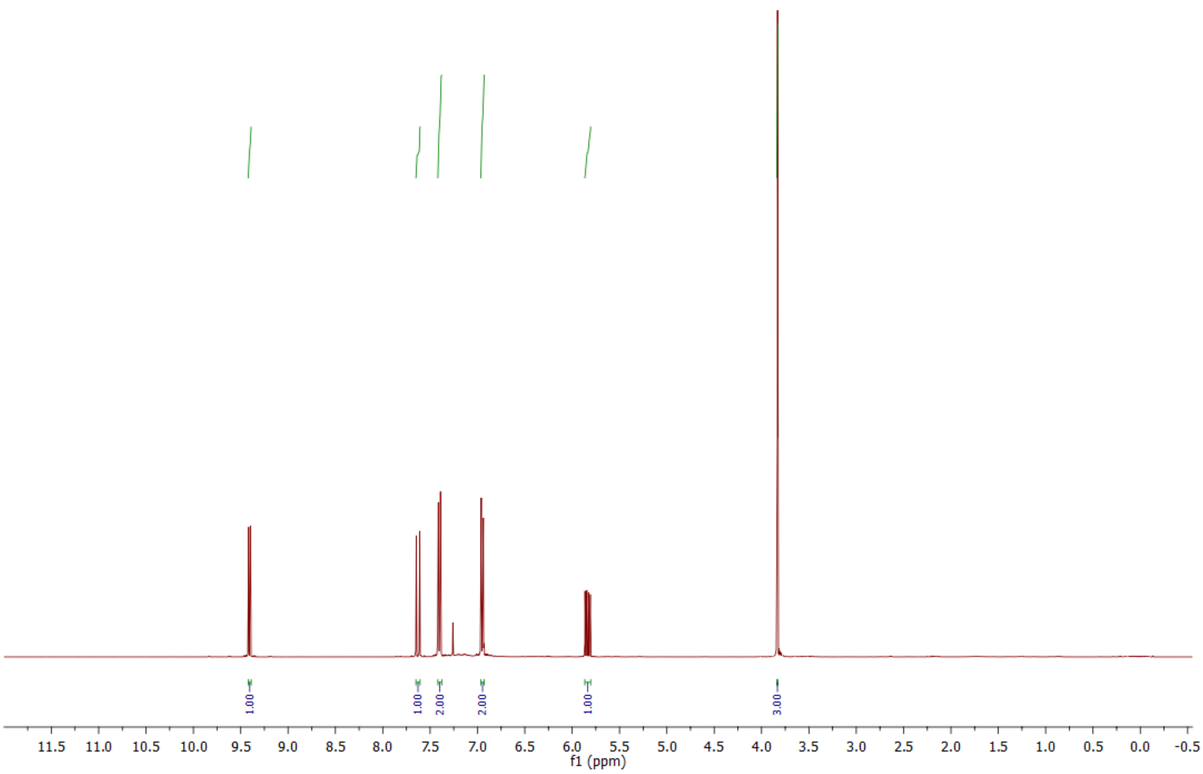

Figure S32. ^{1}H NMR (400 MHz, $CDCl_3$) of product **P14**

Product P14

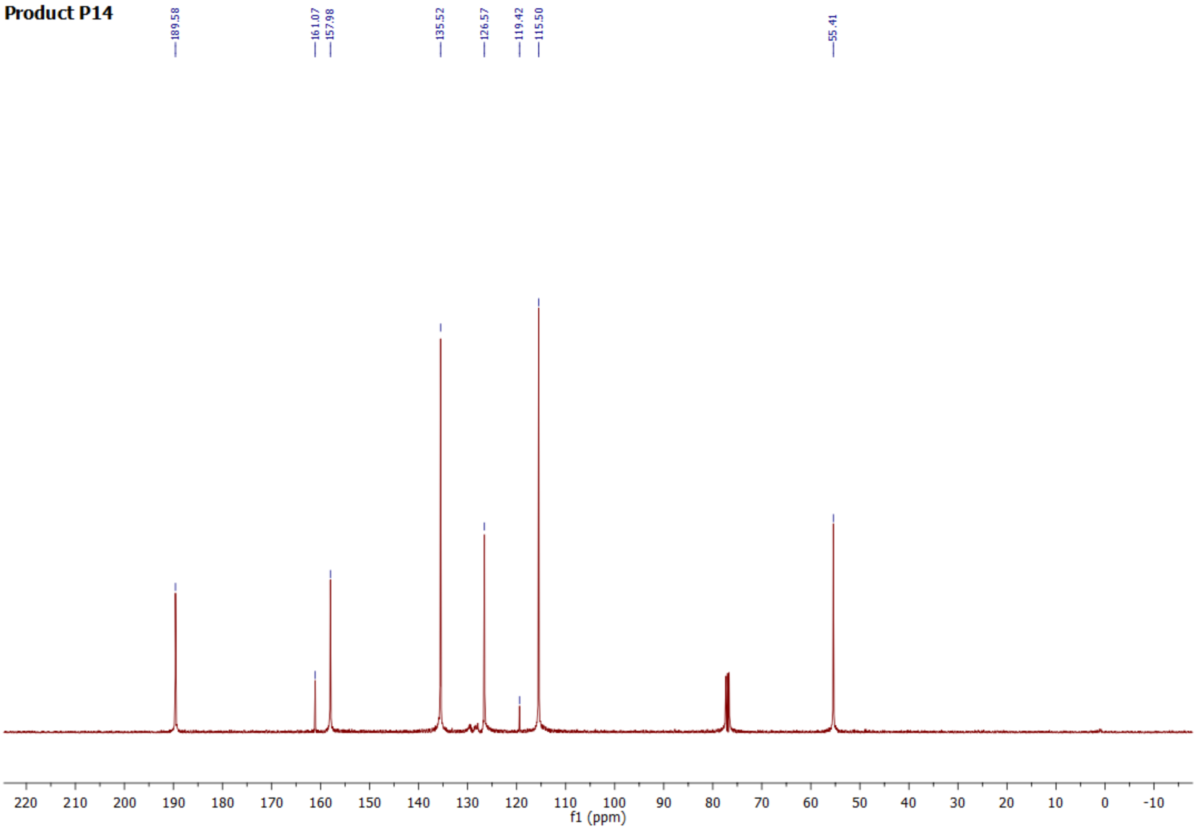

Figure S33. ^{13}C NMR (101 MHz, $CDCl_3$) of product **P14**

Product P15

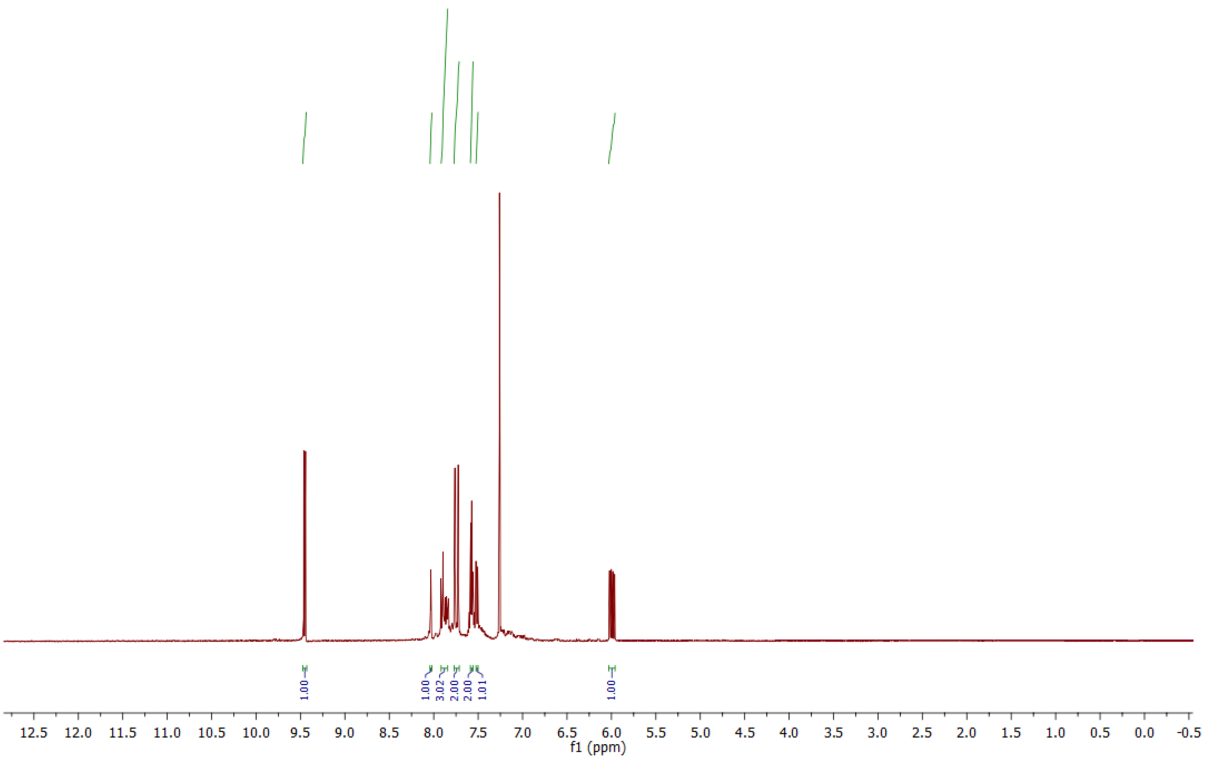

Figure S34. ^{1}H NMR (400 MHz, $CDCl_3$) of product **P15**

Product P15

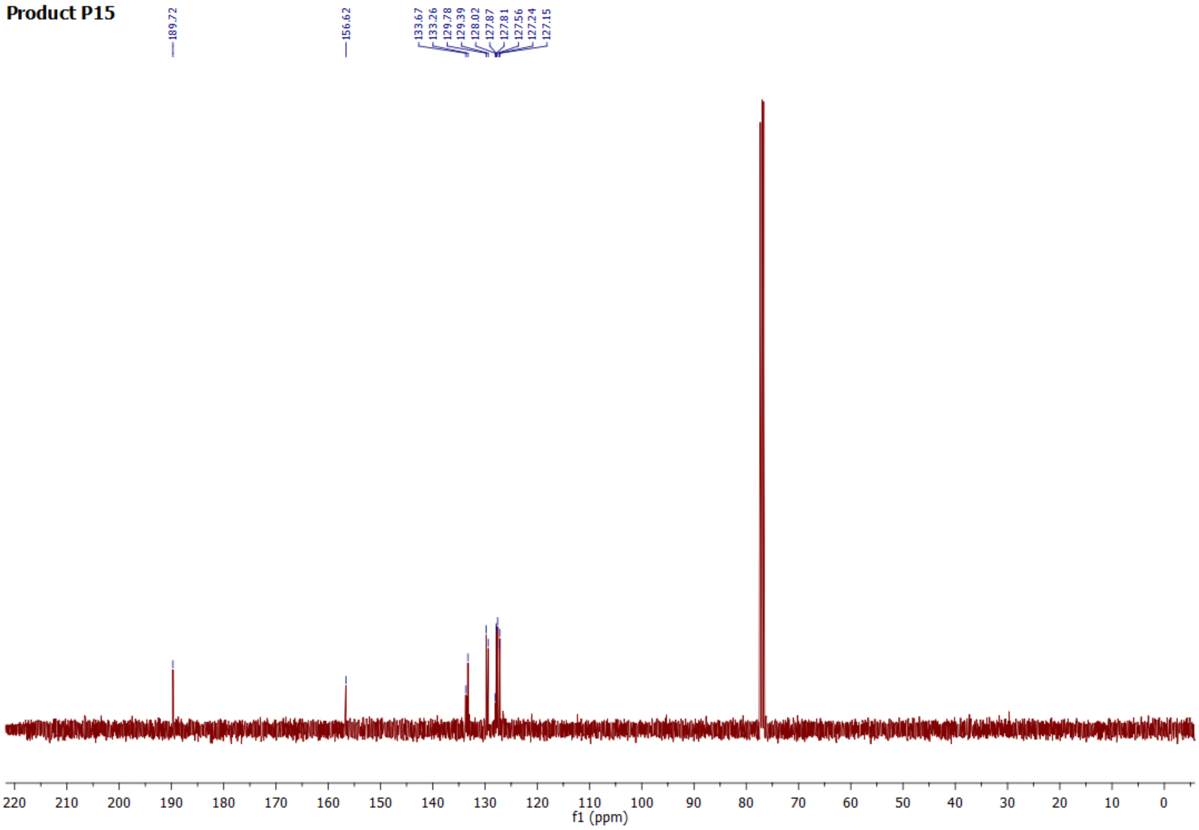

Figure S35. ^{13}C NMR (101 MHz, $CDCl_3$) of product **P15**

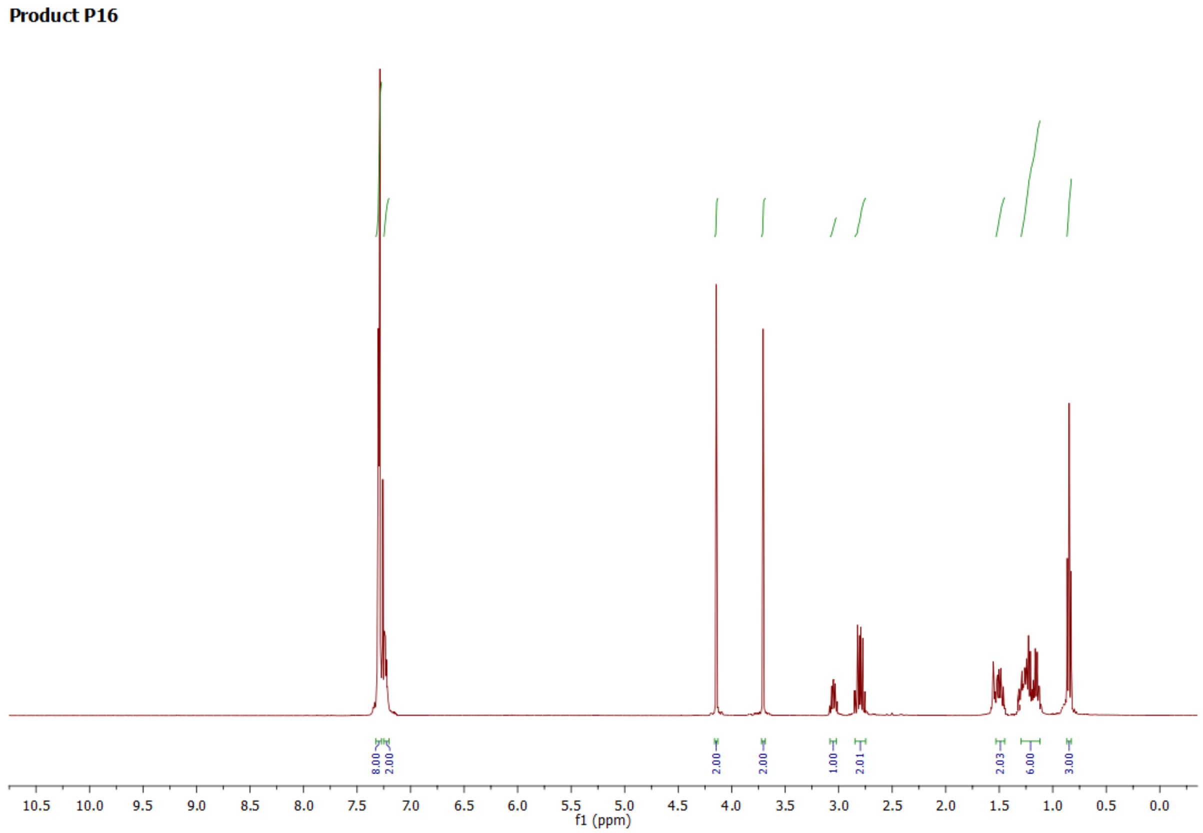

Figure S36. ^{1}H NMR (400 MHz, $CDCl_3$) of product **P16**

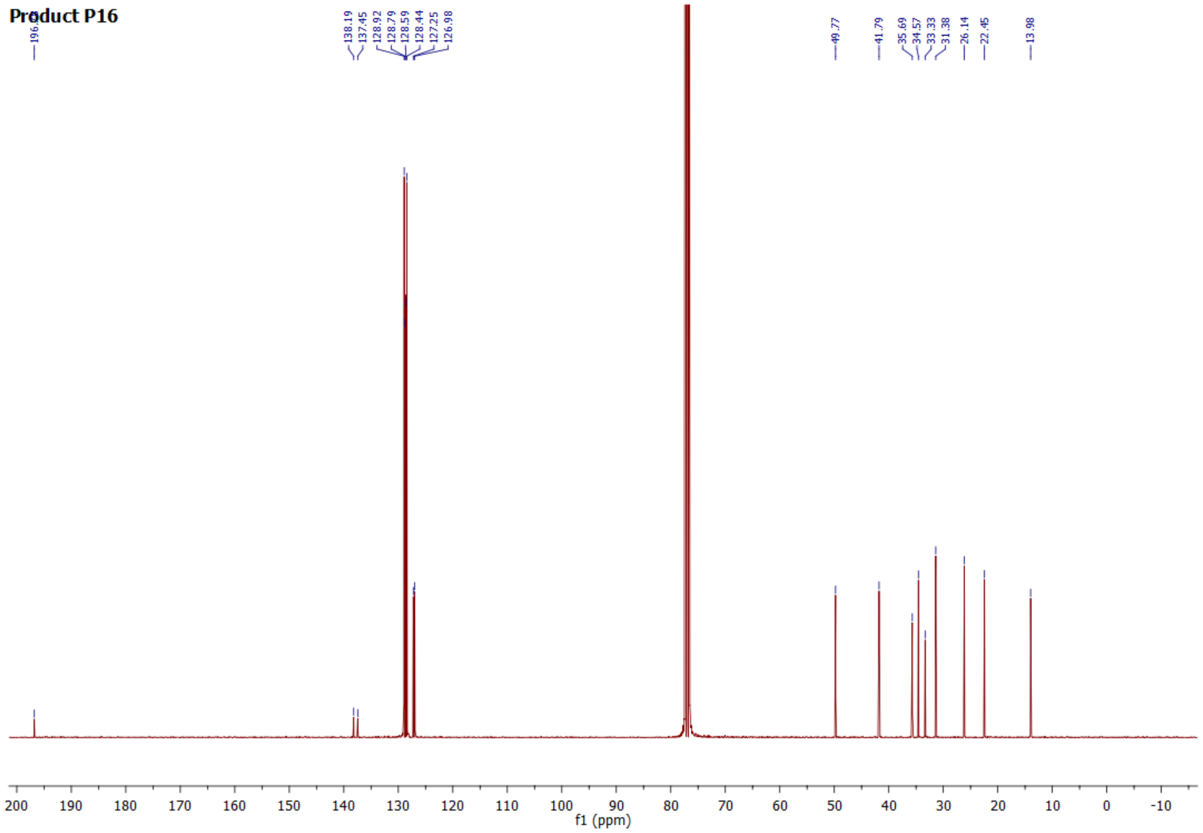

Figure S37. ^{13}C NMR (101 MHz, $CDCl_3$) of product **P16**

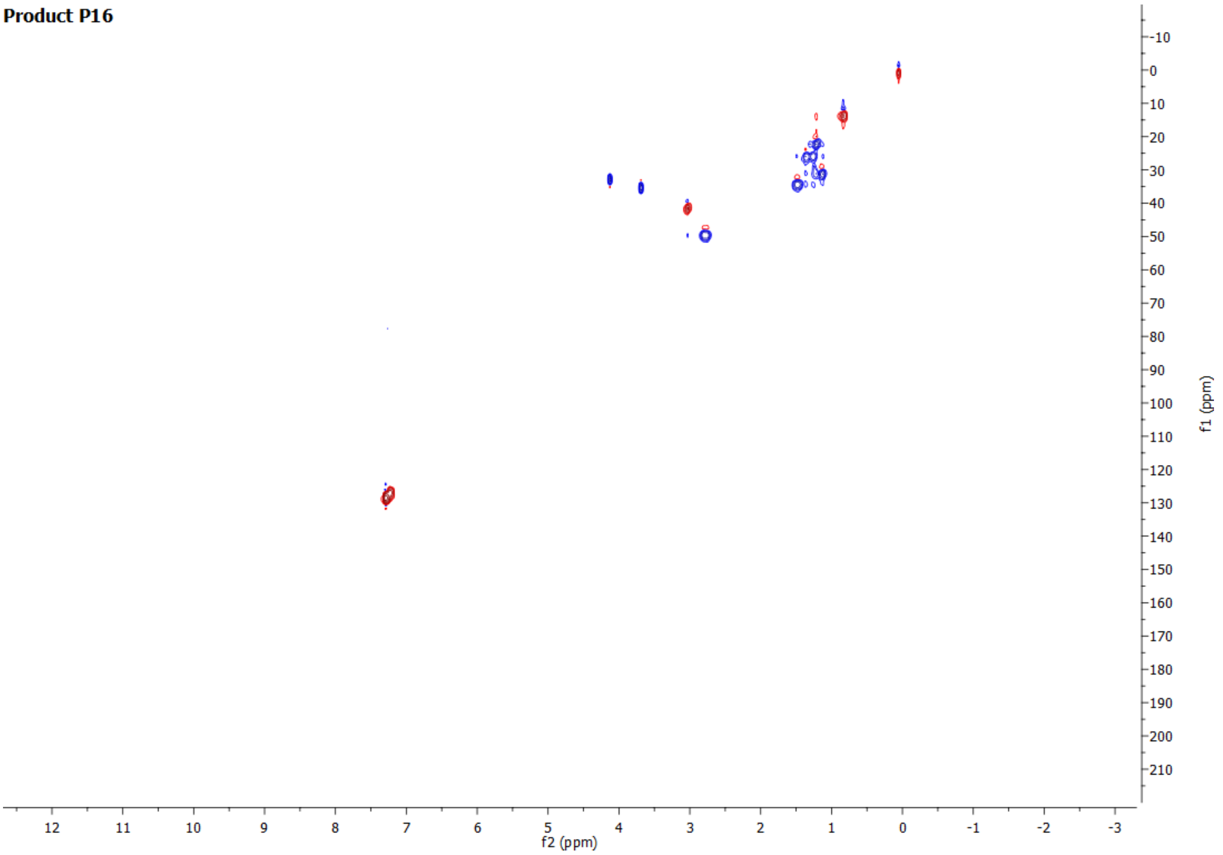

Figure S38. ^{1}H ^{13}C HSQC (600 MHz, $CDCl_3$) of product **P16**

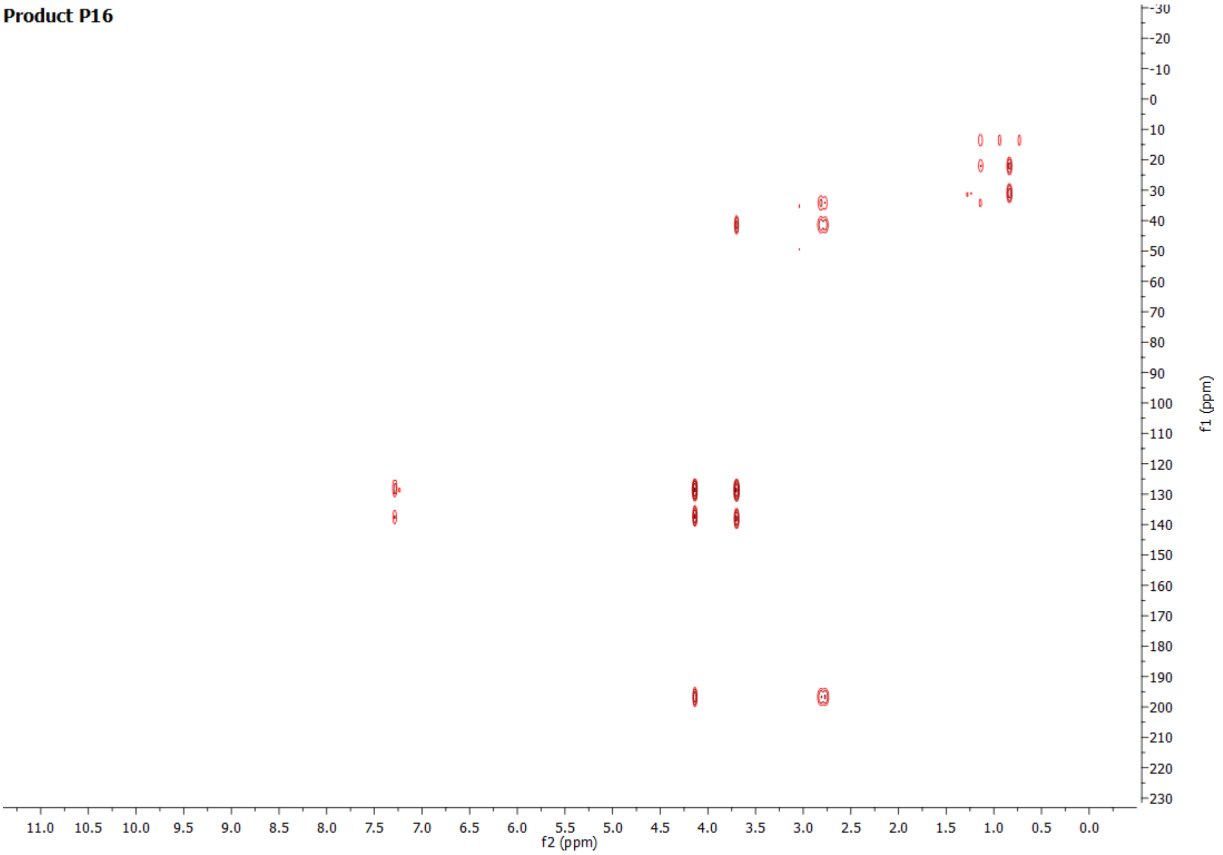

Figure S39. ^{1}H ^{13}C HMBC (600 MHz, $CDCl_3$) of product **P16**

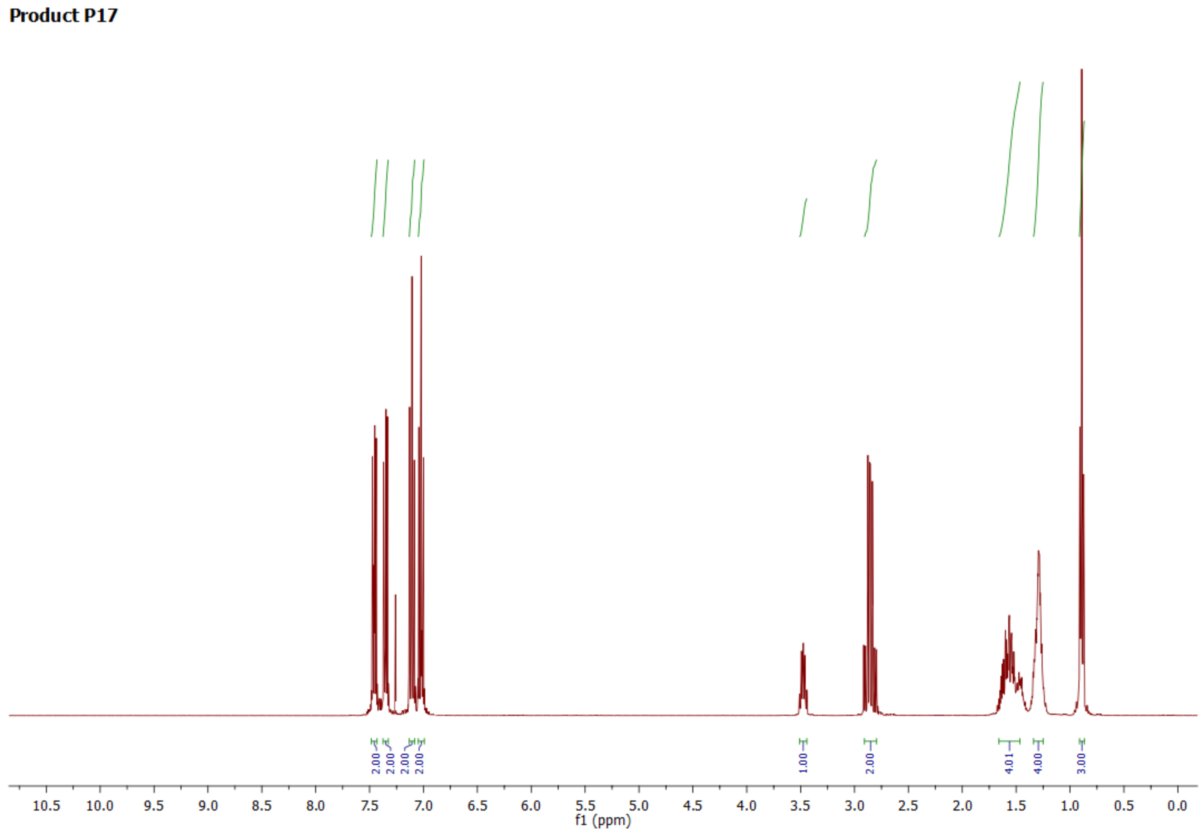

Figure S10. ^{1}H NMR (400 MHz, $CDCl_3$) of product **P17**

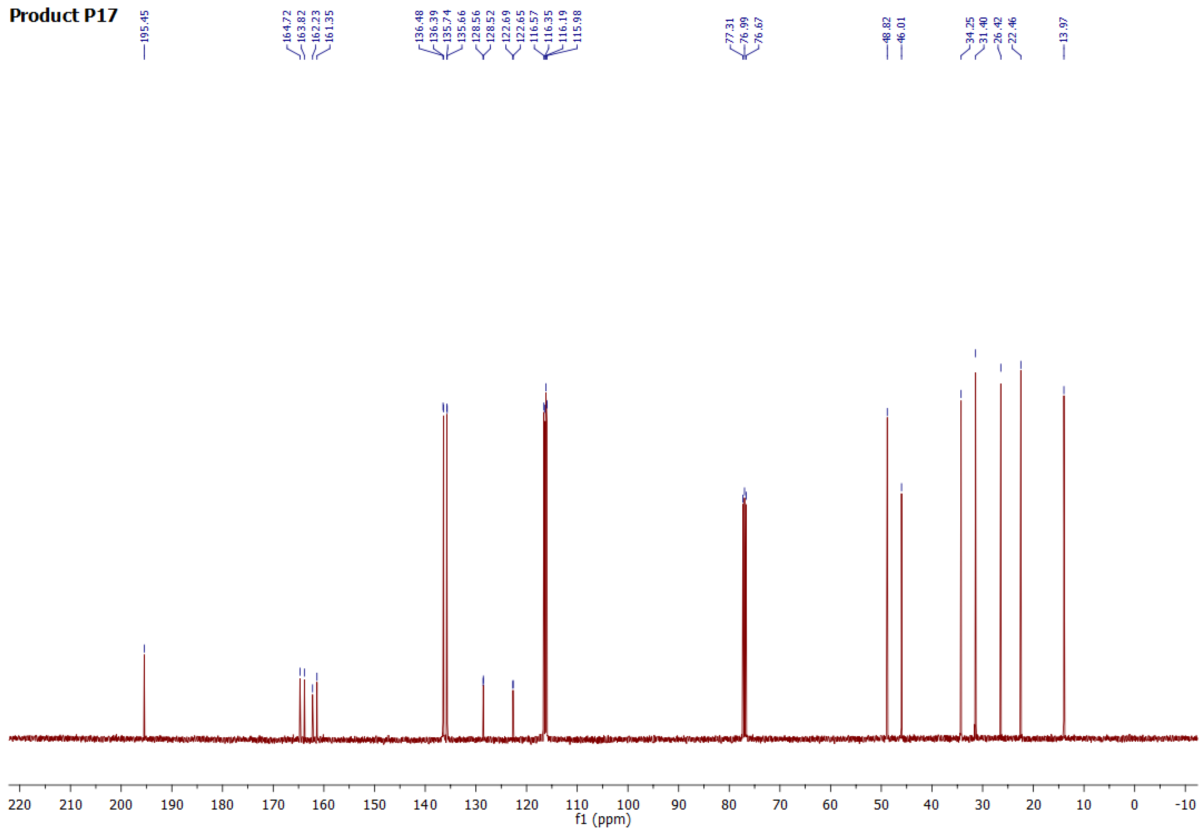

Figure S41. ^{13}C NMR (101 MHz, $CDCl_3$) of product **P17**

Product P17

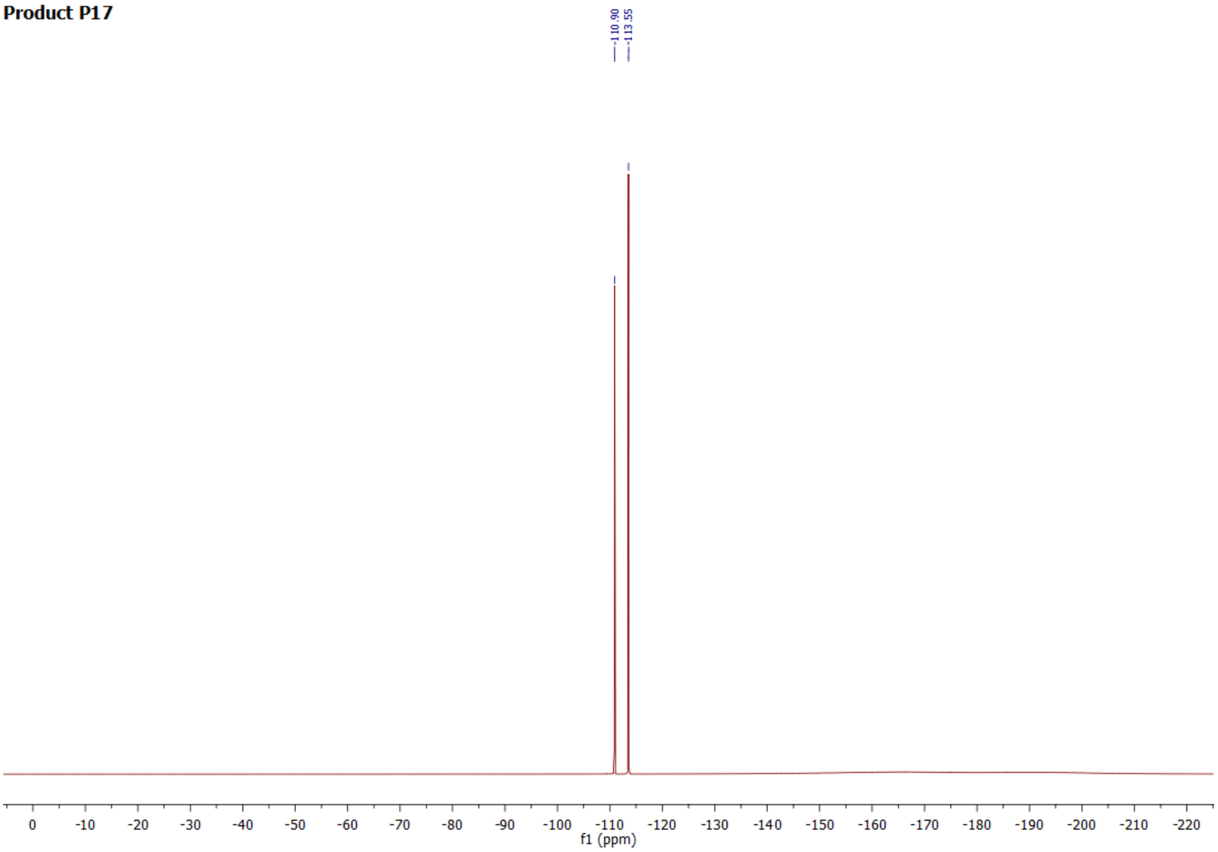

Figure S42. ^{19}F NMR (376 MHz, $CDCl_3$) of product **P17**

Product P18

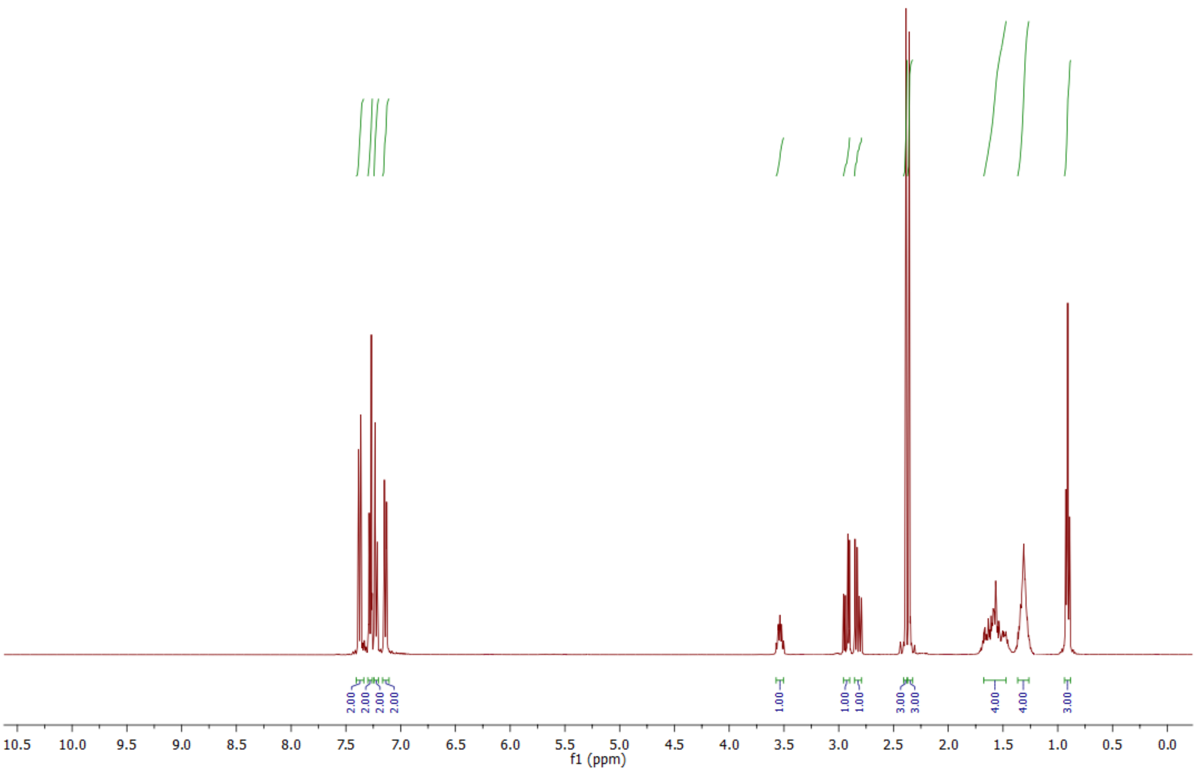

Figure S43. ^{1}H NMR (400 MHz, $CDCl_3$) of product **P18**

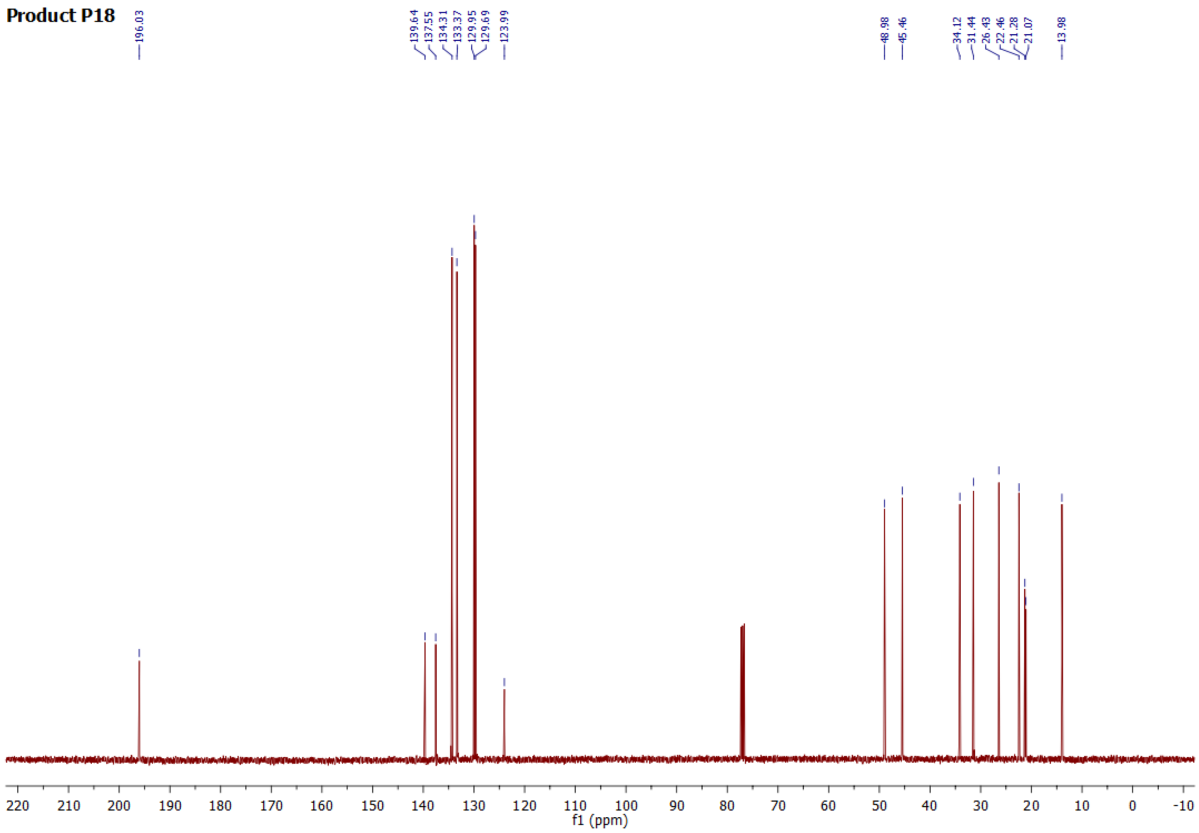

Figure S44. ^{13}C NMR (101 MHz, $CDCl_3$) of product **P18**

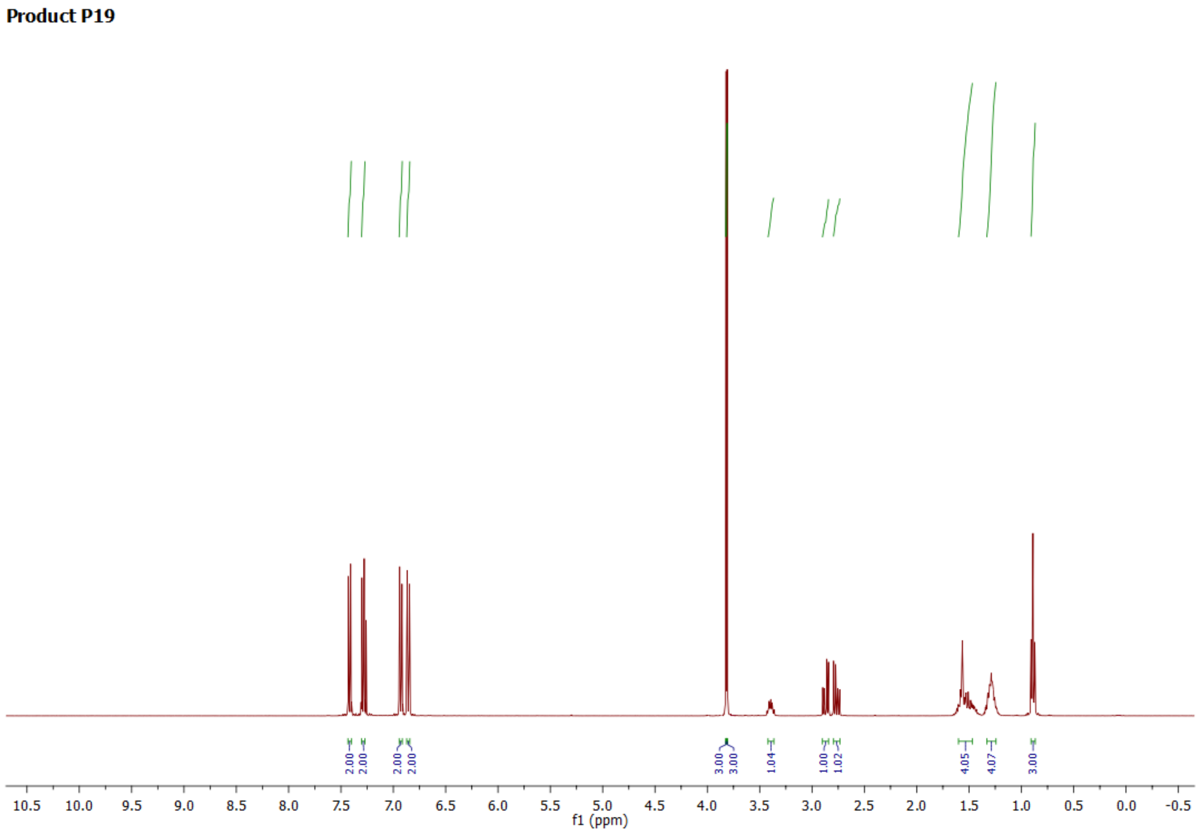

Figure S44. ^{1}H NMR (400 MHz, $CDCl_3$) of product **P19**

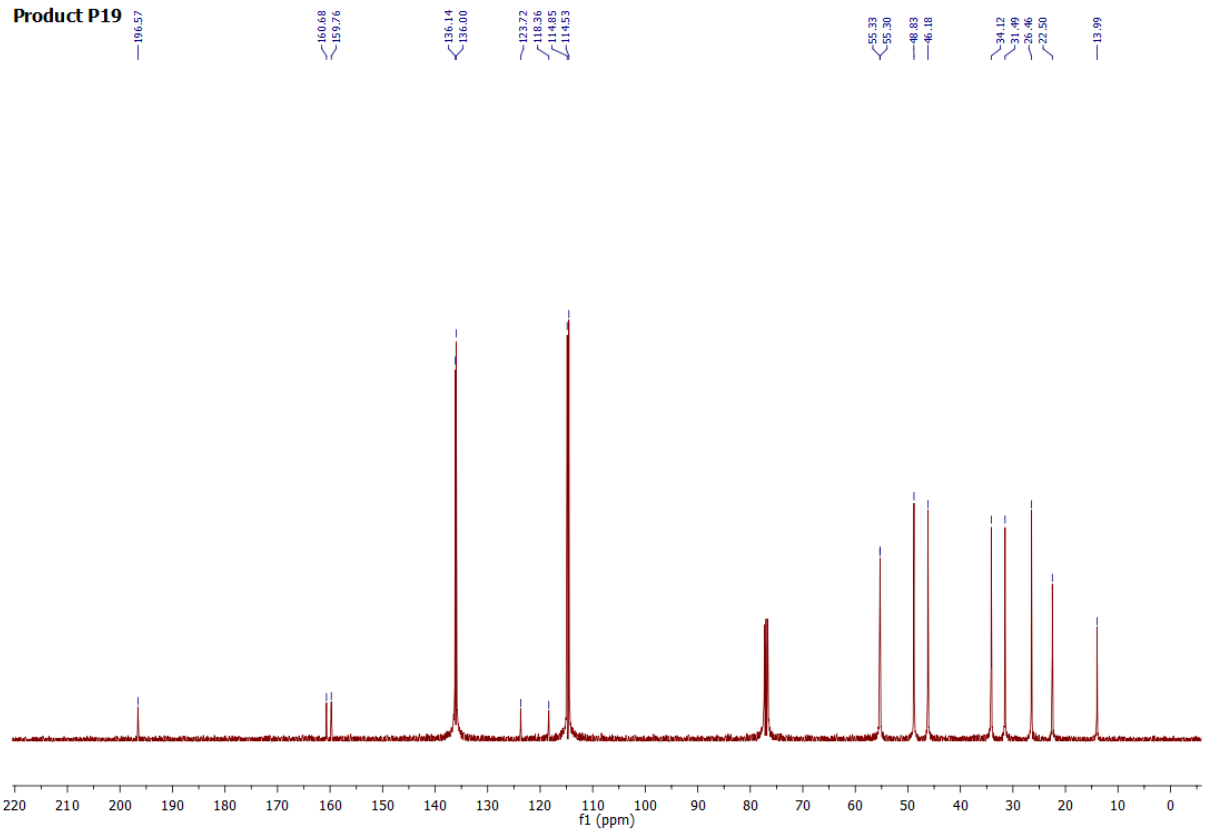

Figure S45. ^{13}C NMR (101 MHz, $CDCl_3$) of product **P19**

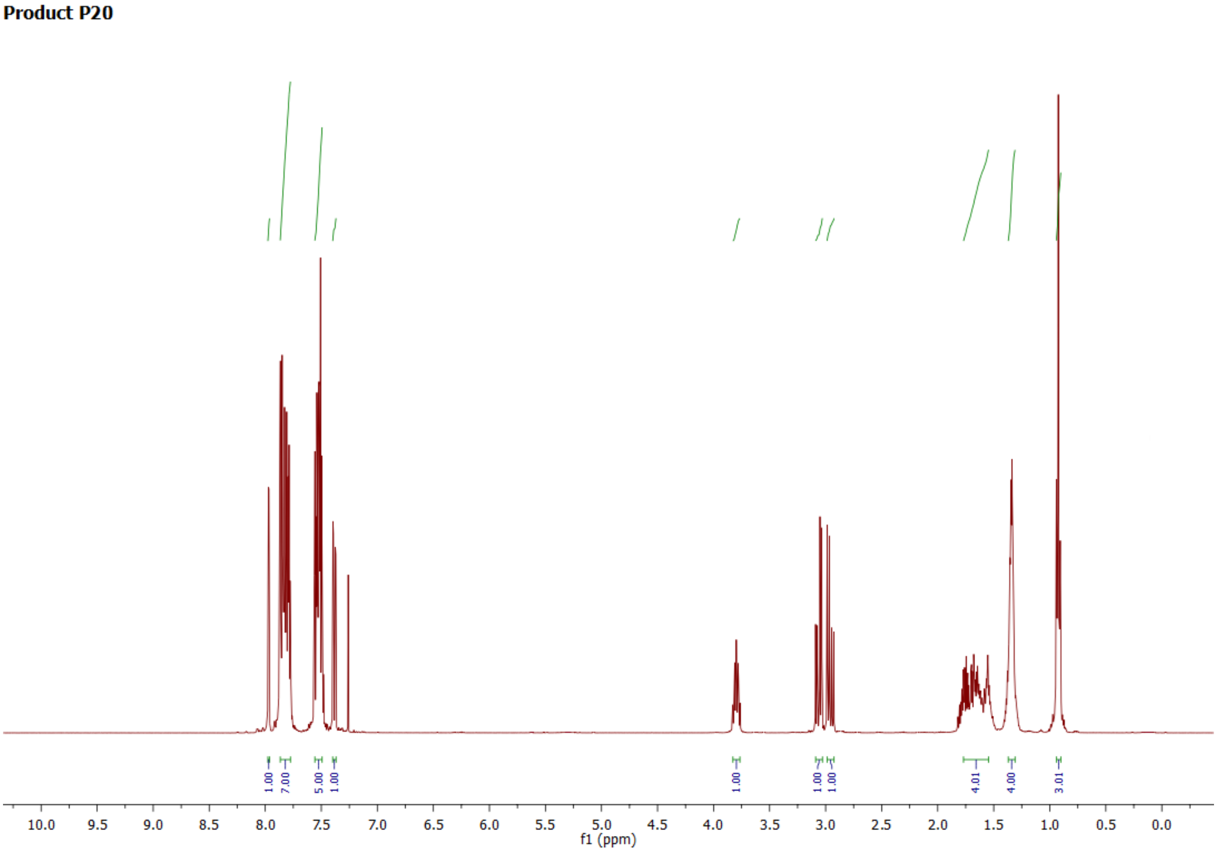

Figure S46. ^{1}H NMR (400 MHz, $CDCl_3$) of product **P20**

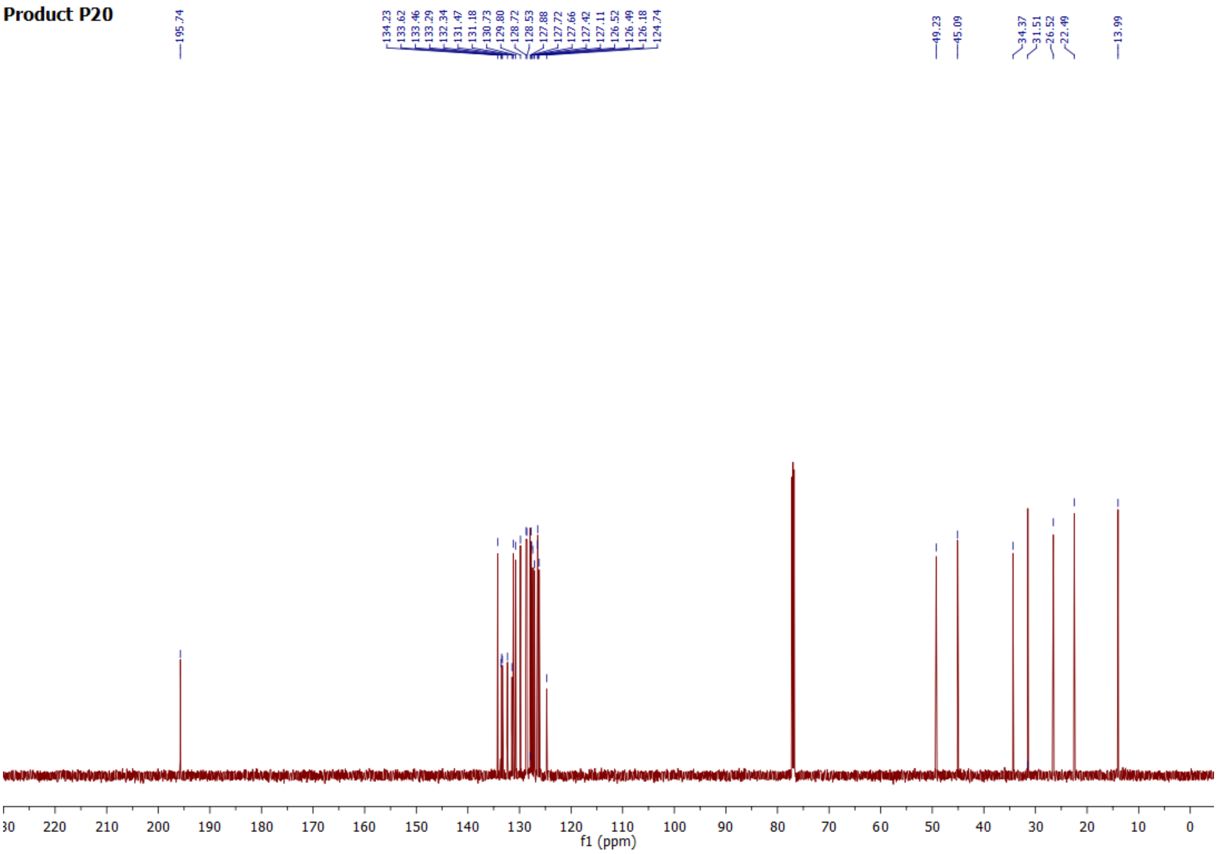

Figure S47. ^{13}C NMR (101 MHz, $CDCl_3$) of product **P20**

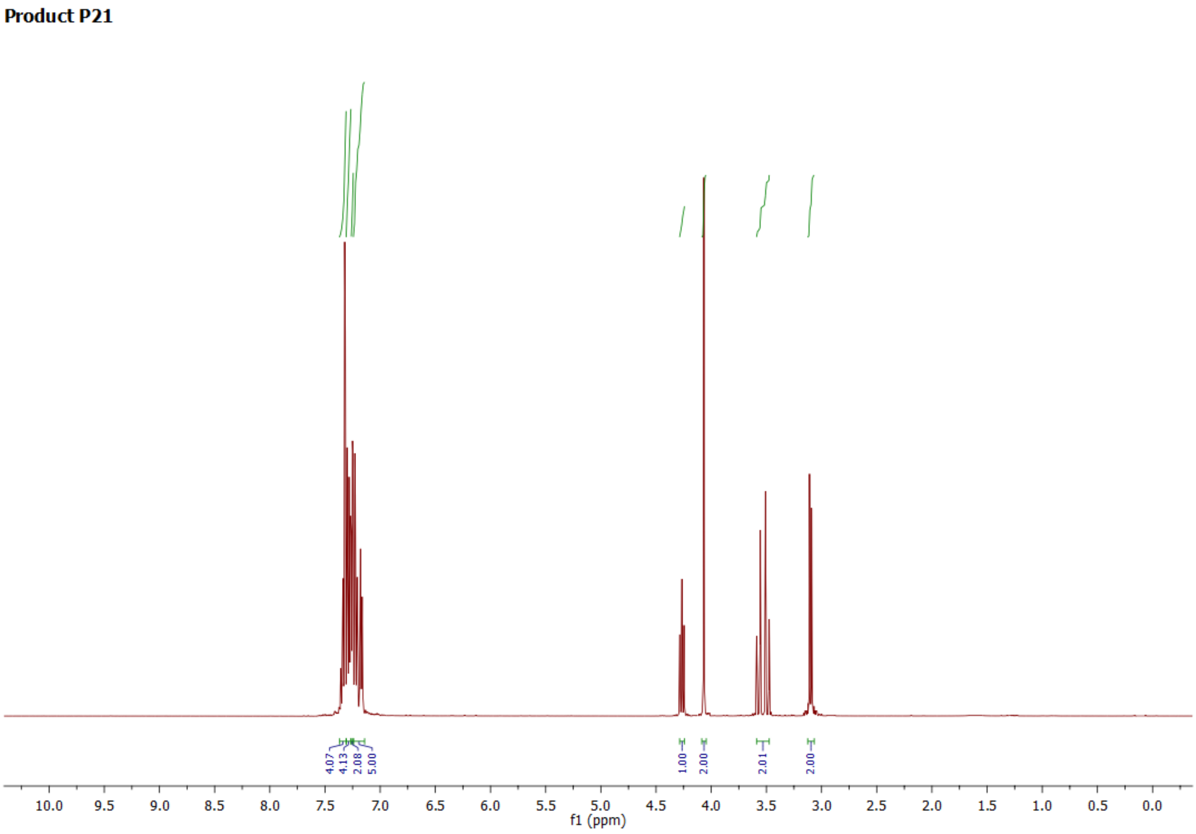

Figure S48. ^{1}H NMR (400 MHz, $CDCl_3$) of product **P21**

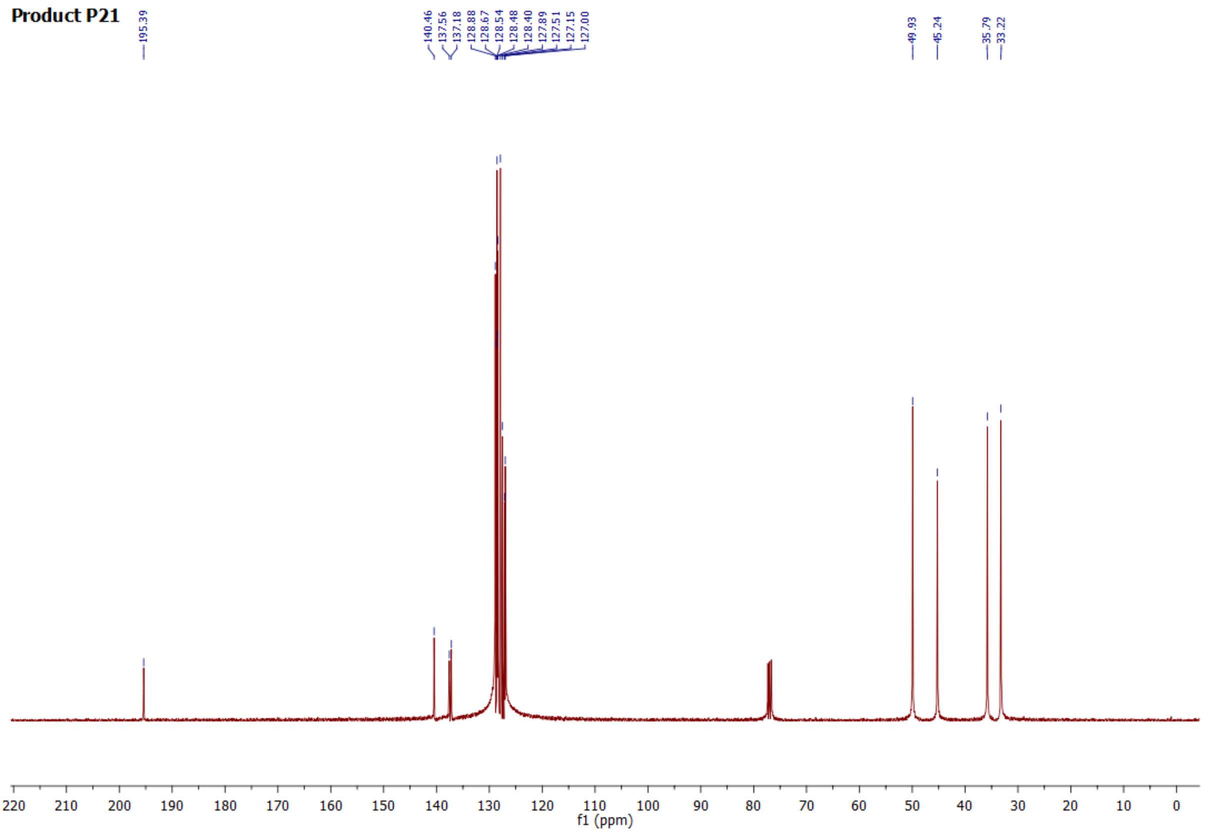

Figure S49. ^{13}C NMR (101 MHz, $CDCl_3$) of product **P21**

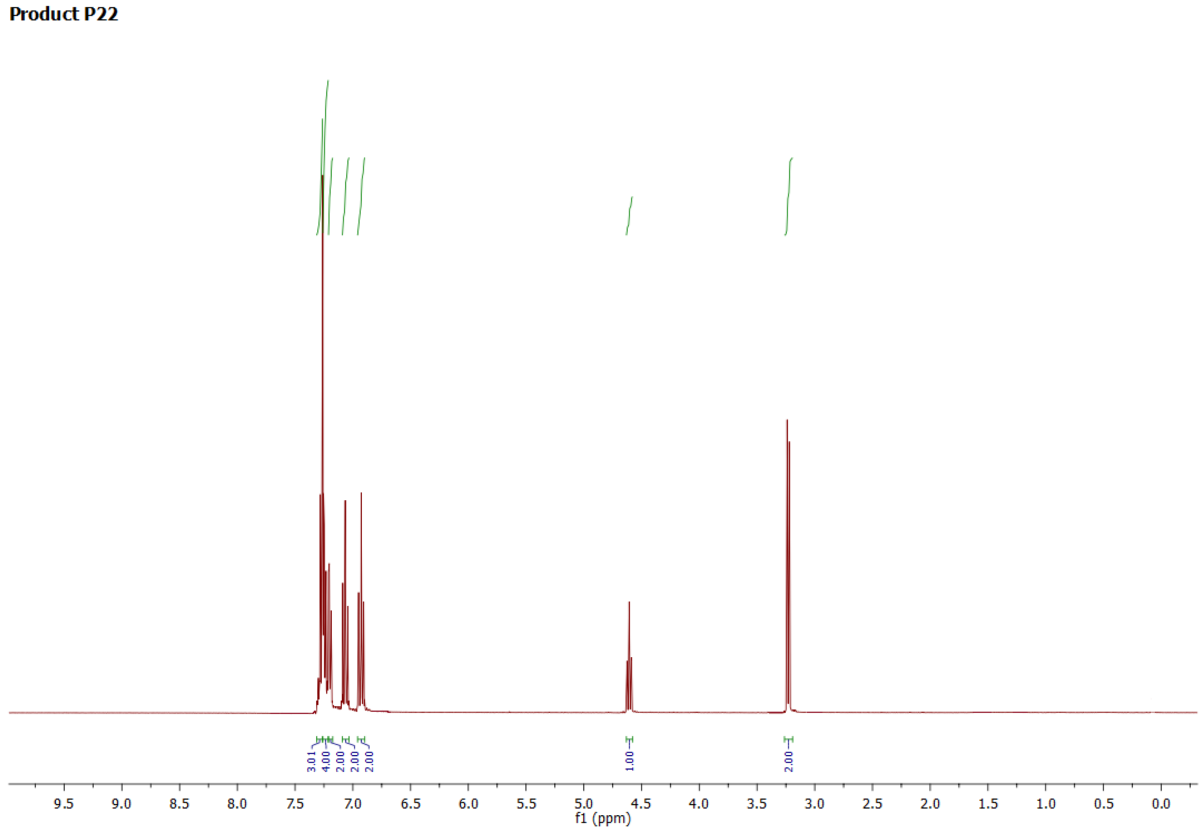

Figure S50. ^{1}H NMR (400 MHz, $CDCl_3$) of product **P22**

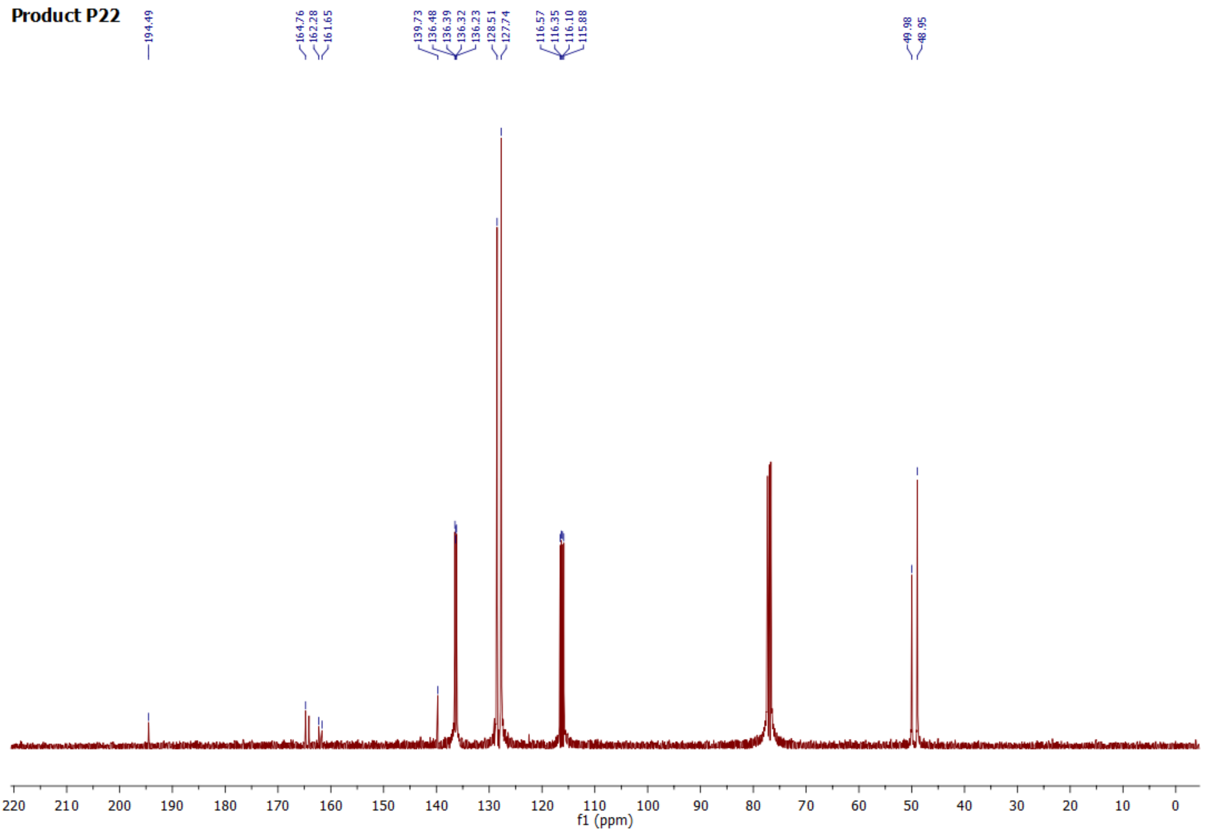

Figure S51. ^{13}C NMR (101 MHz, $CDCl_3$) of product **P22**

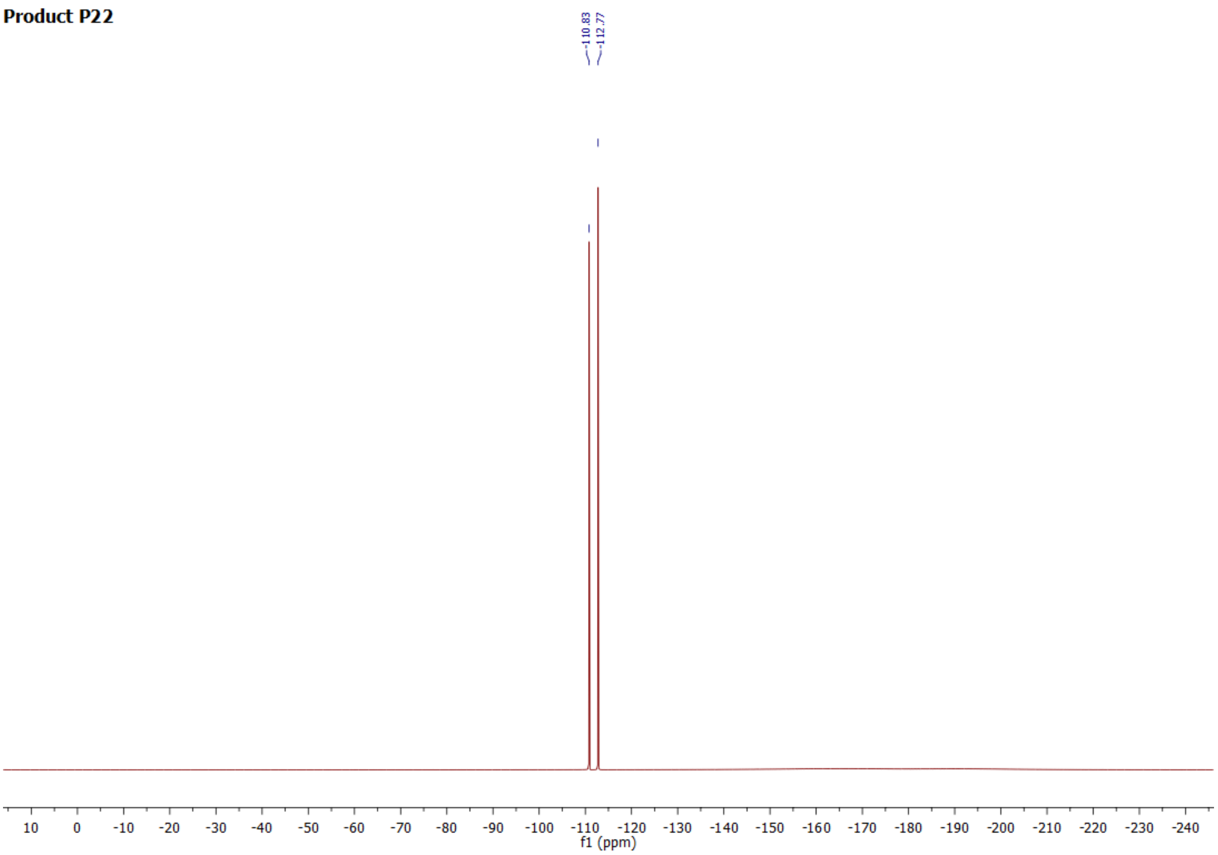

Figure S52. ^{19}F NMR (376 MHz, $CDCl_3$) of product **P22**

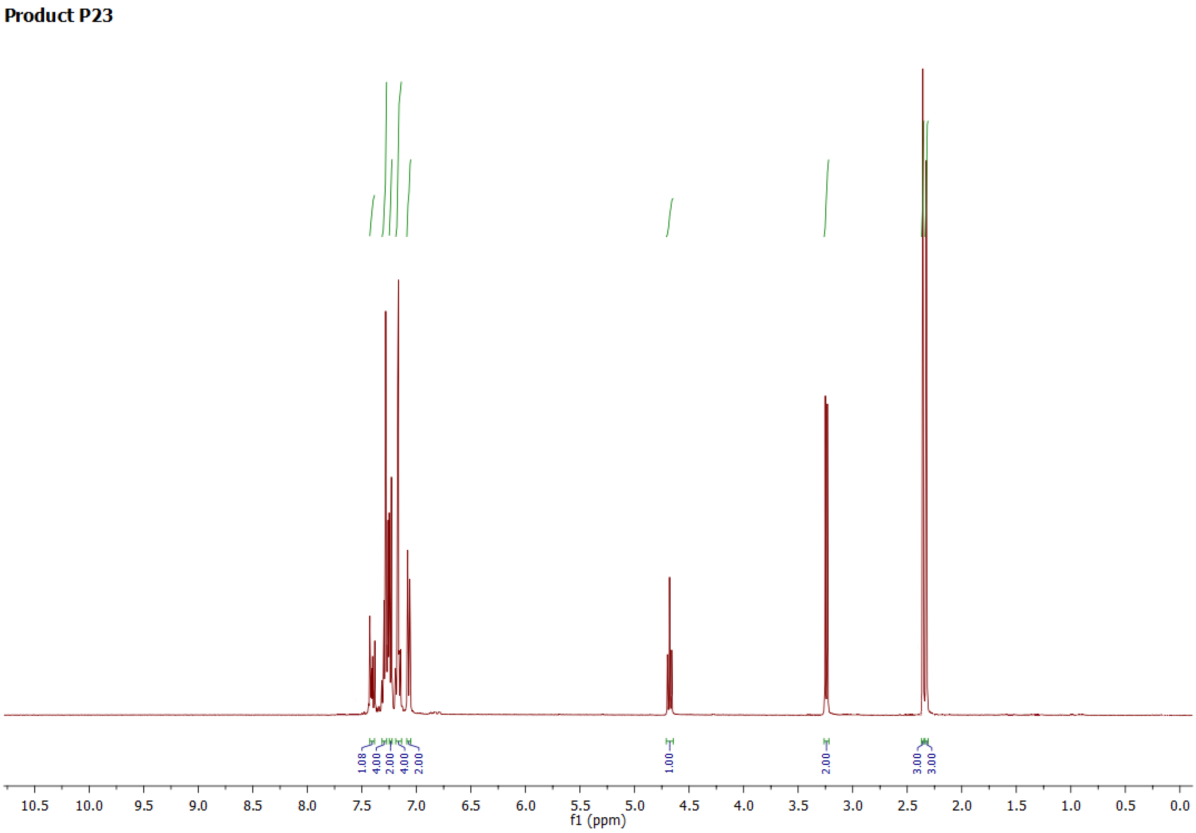

Figure S53. ^{1}H NMR (400 MHz, $CDCl_3$) of product **P23**

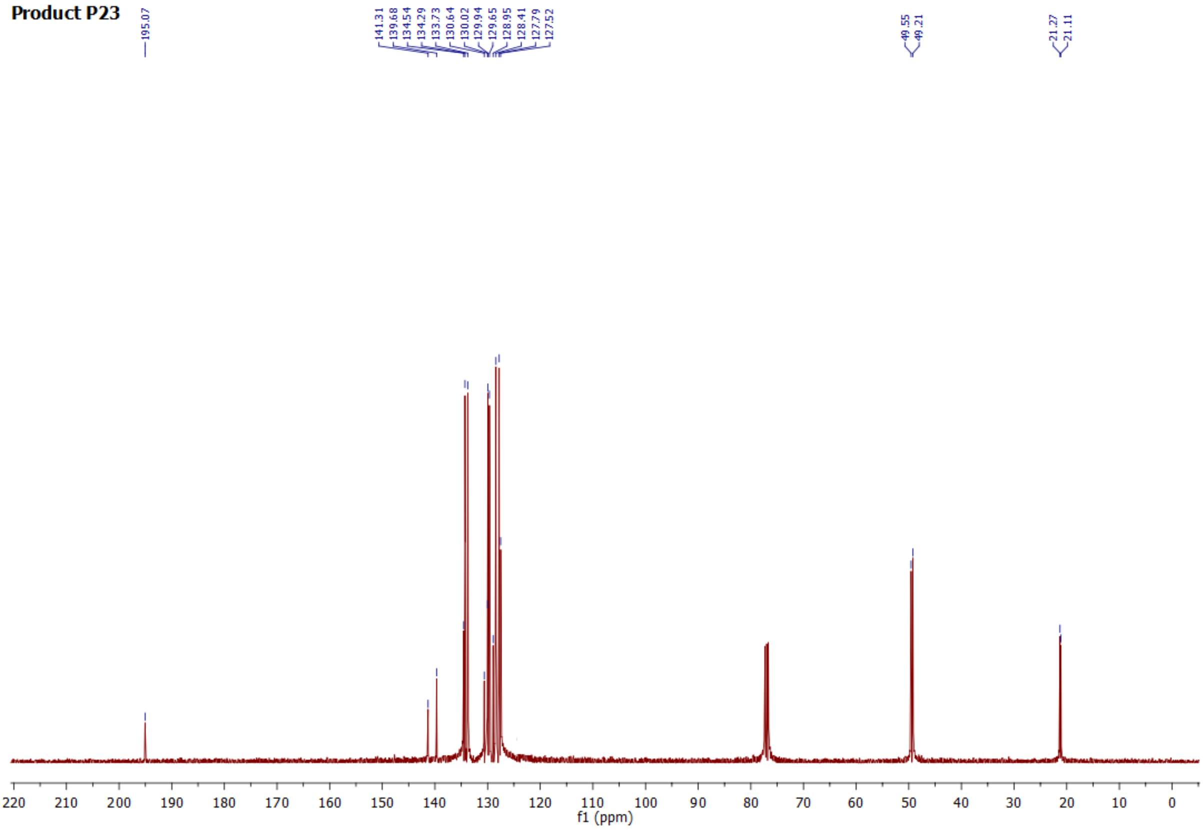

Figure S54. ^{13}C NMR (101 MHz, $CDCl_3$) of product **P23**

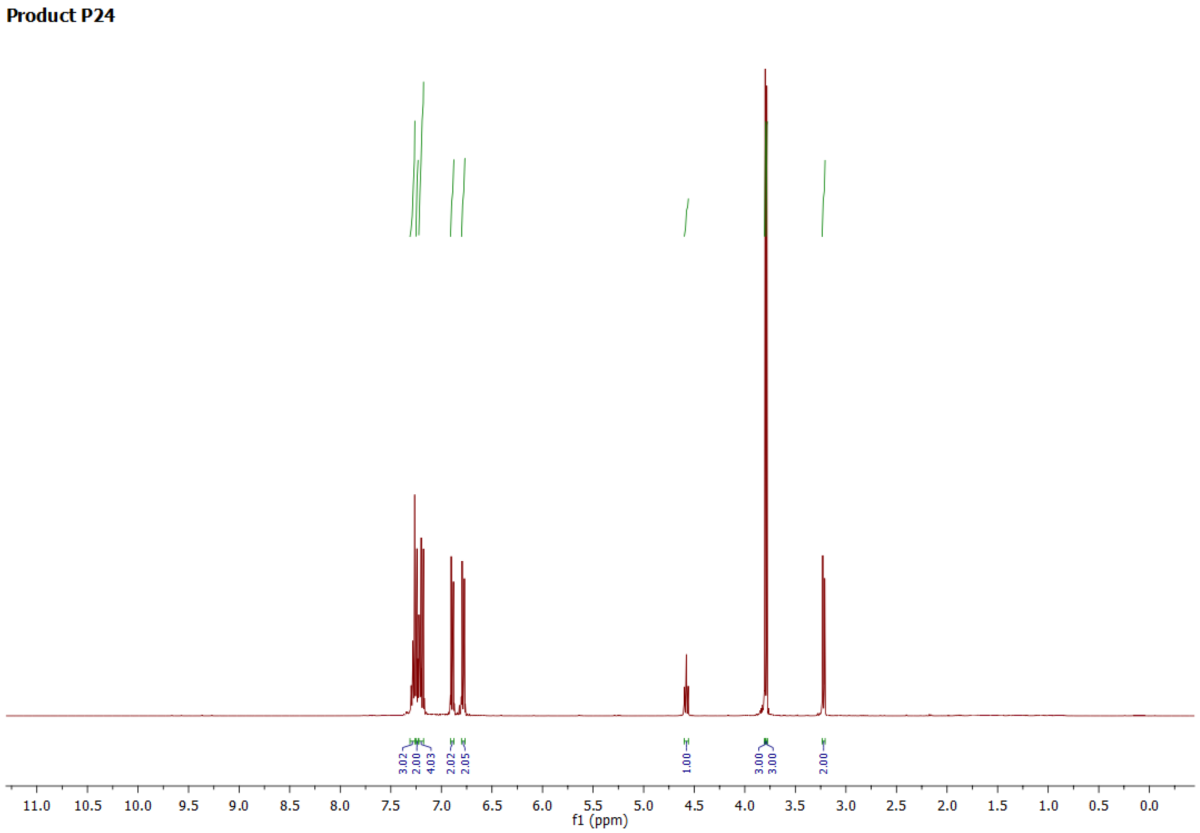

Figure S55. ^{1}H NMR (400 MHz, $CDCl_3$) of product **P24**

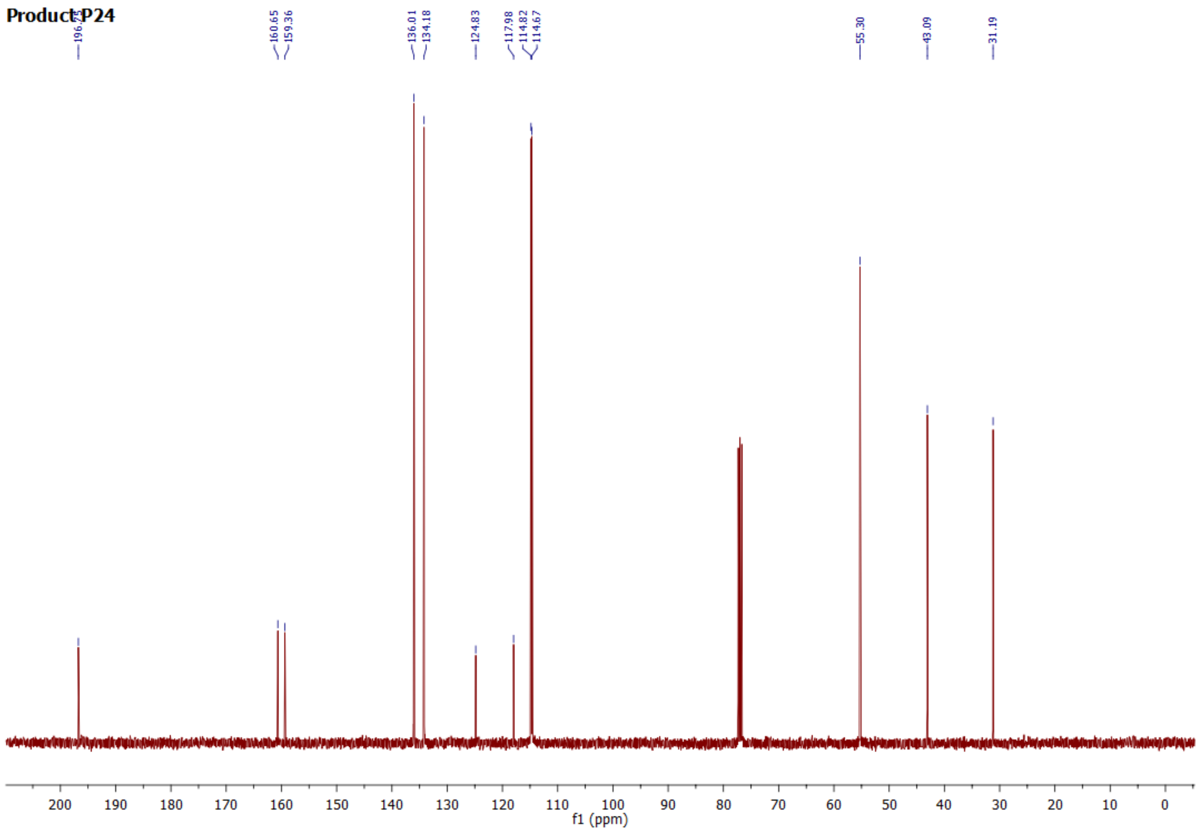

Figure S56. ^{13}C NMR (101 MHz, $CDCl_3$) of product **P24**

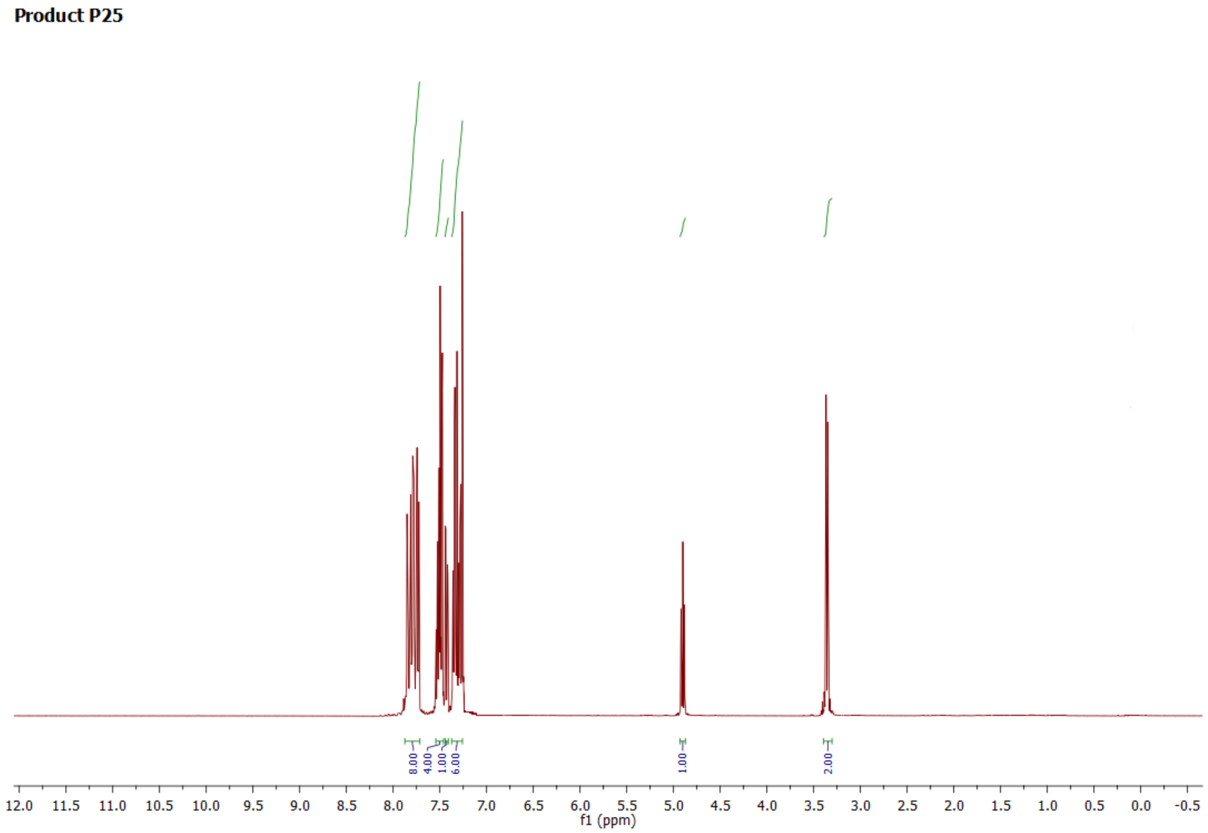

Figure S57. ^{1}H NMR (400 MHz, $CDCl_3$) of product **P25**

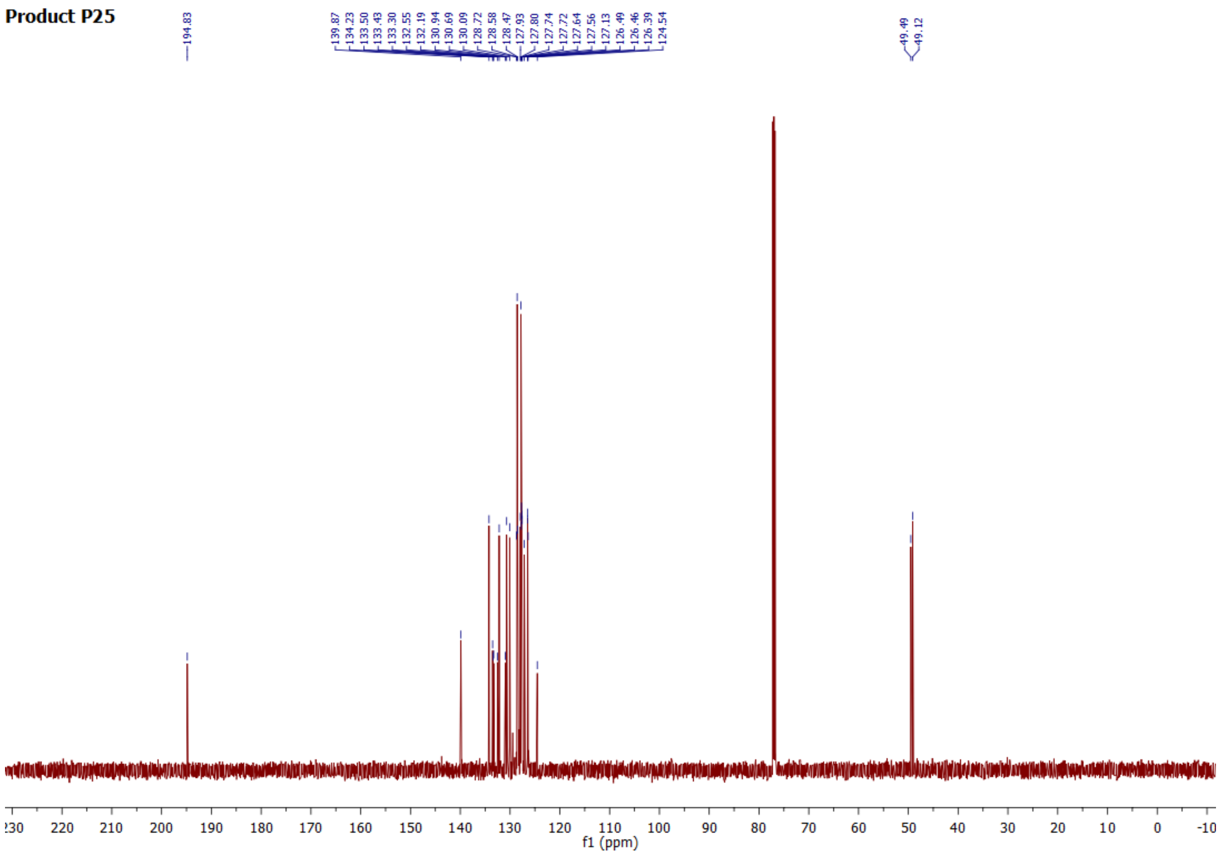

Figure S58. ^{13}C NMR (101 MHz, $CDCl_3$) of product **P25**

Product P26

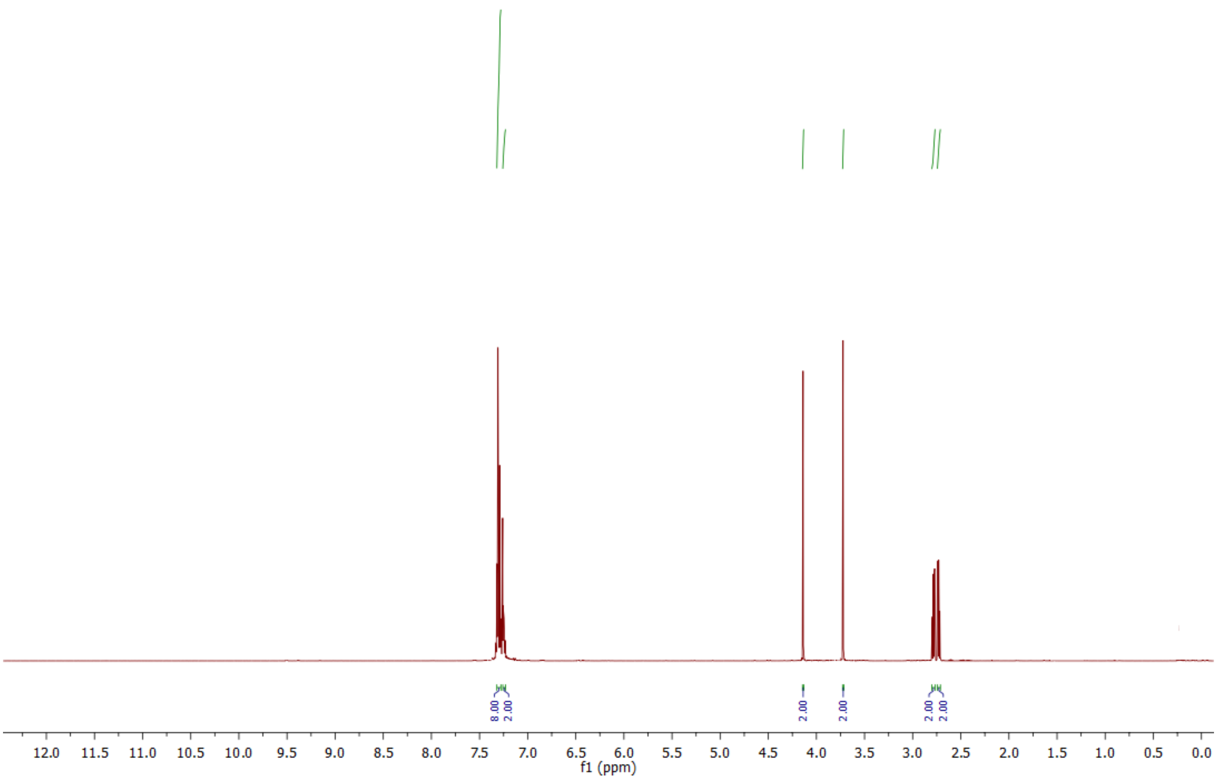

Figure S59. ^{1}H NMR (400 MHz, $CDCl_3$) of product **P26**

Product P26

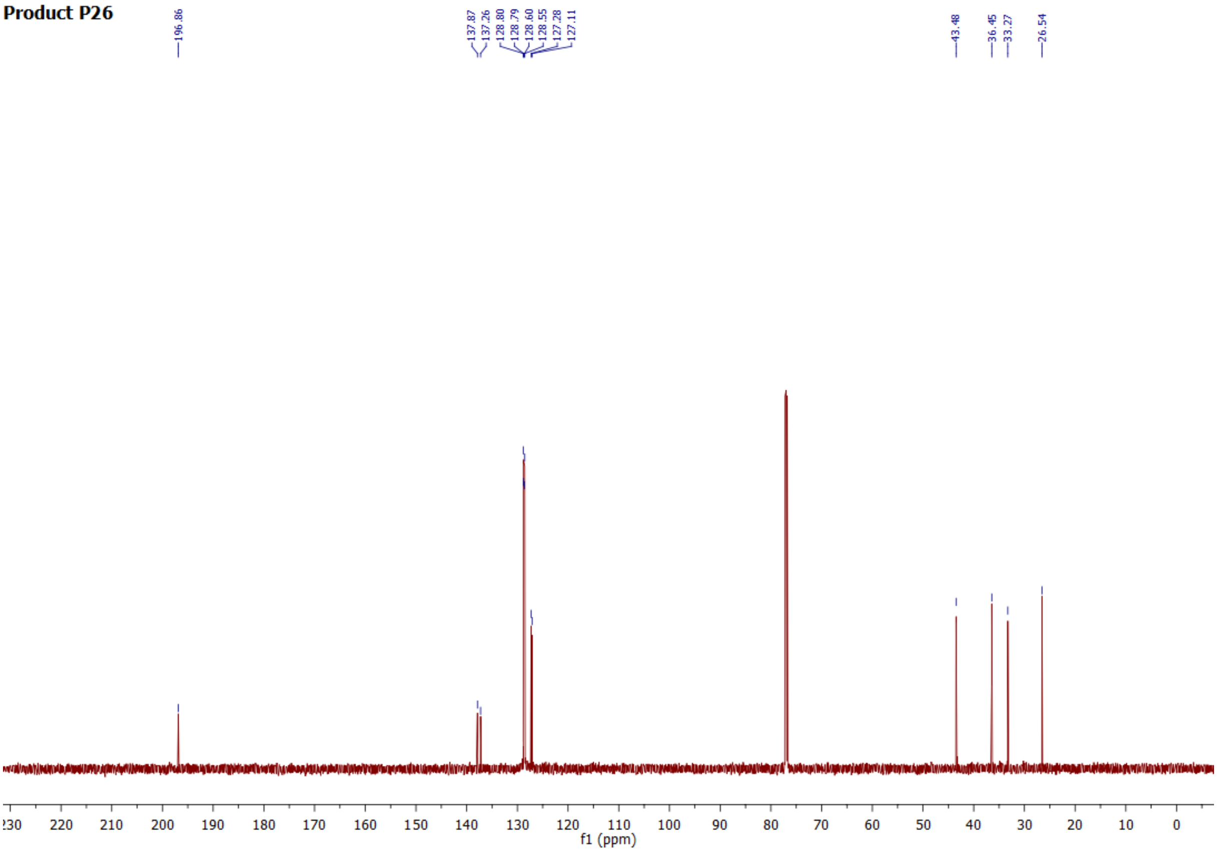

Figure S60. ^{13}C NMR (101 MHz, $CDCl_3$) of product **P26**

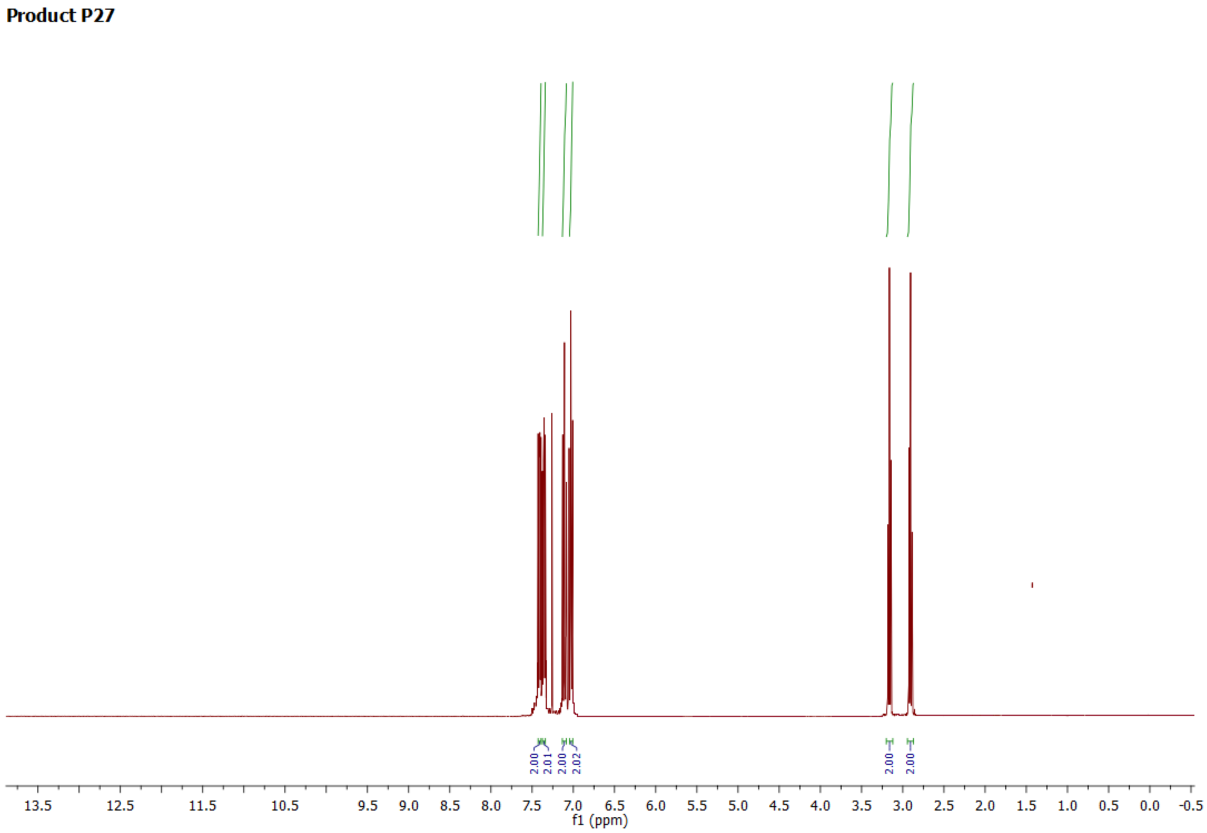

Figure S61. ^{1}H NMR (400 MHz, $CDCl_3$) of product **P27**

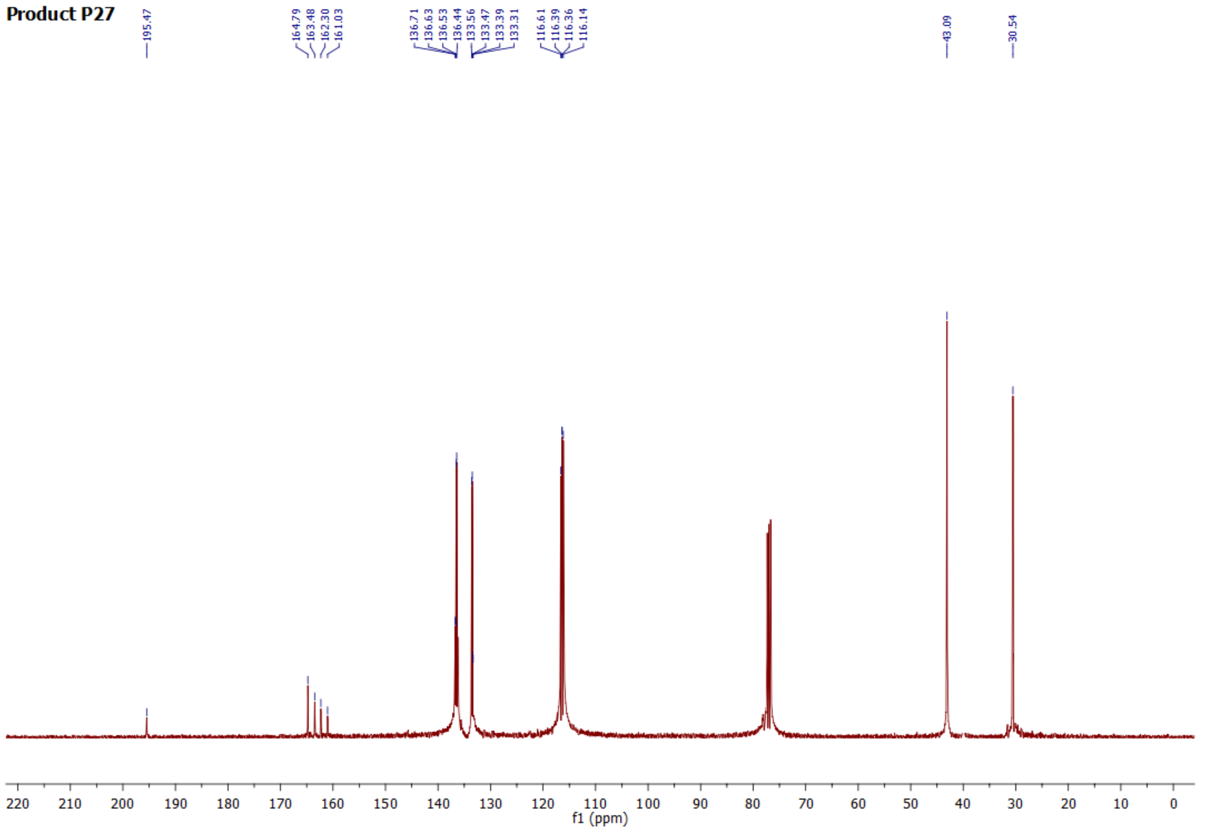

Figure S62. ^{13}C NMR (101 MHz, $CDCl_3$) of product **P27**

Product P27

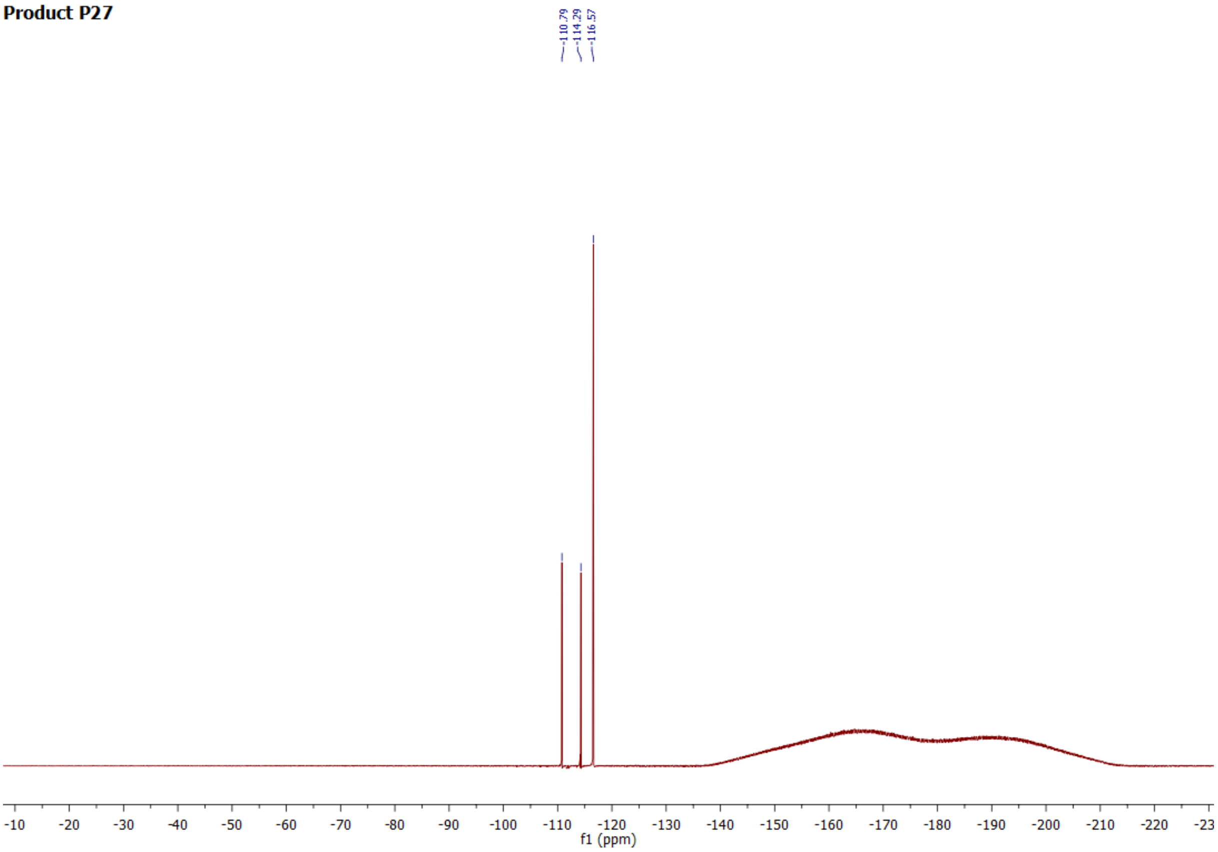

Figure S63. ^{19}F NMR (376 MHz, $CDCl_3$) of product **P27**

Product P28

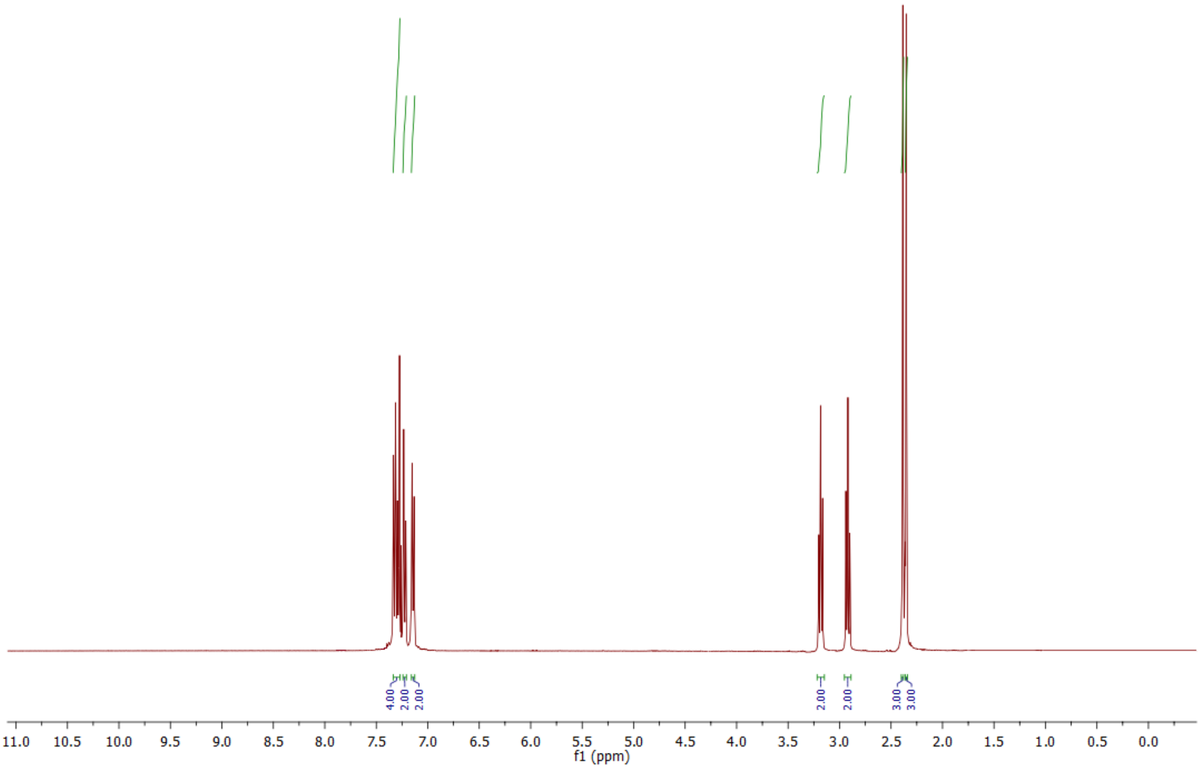

Figure S64. ^{1}H NMR (400 MHz, $CDCl_3$) of product **P28**

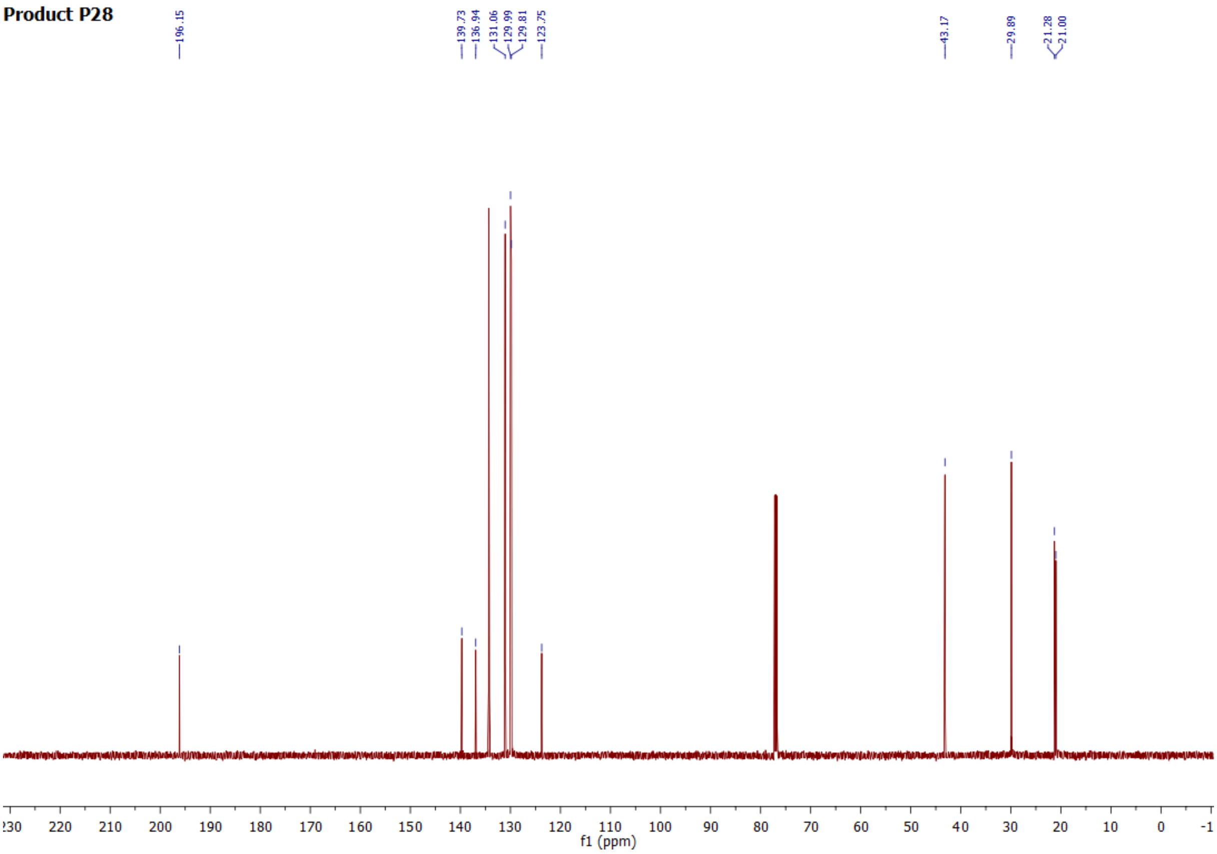

Figure S65. ^{13}C NMR (101 MHz, $CDCl_3$) of product **P28**

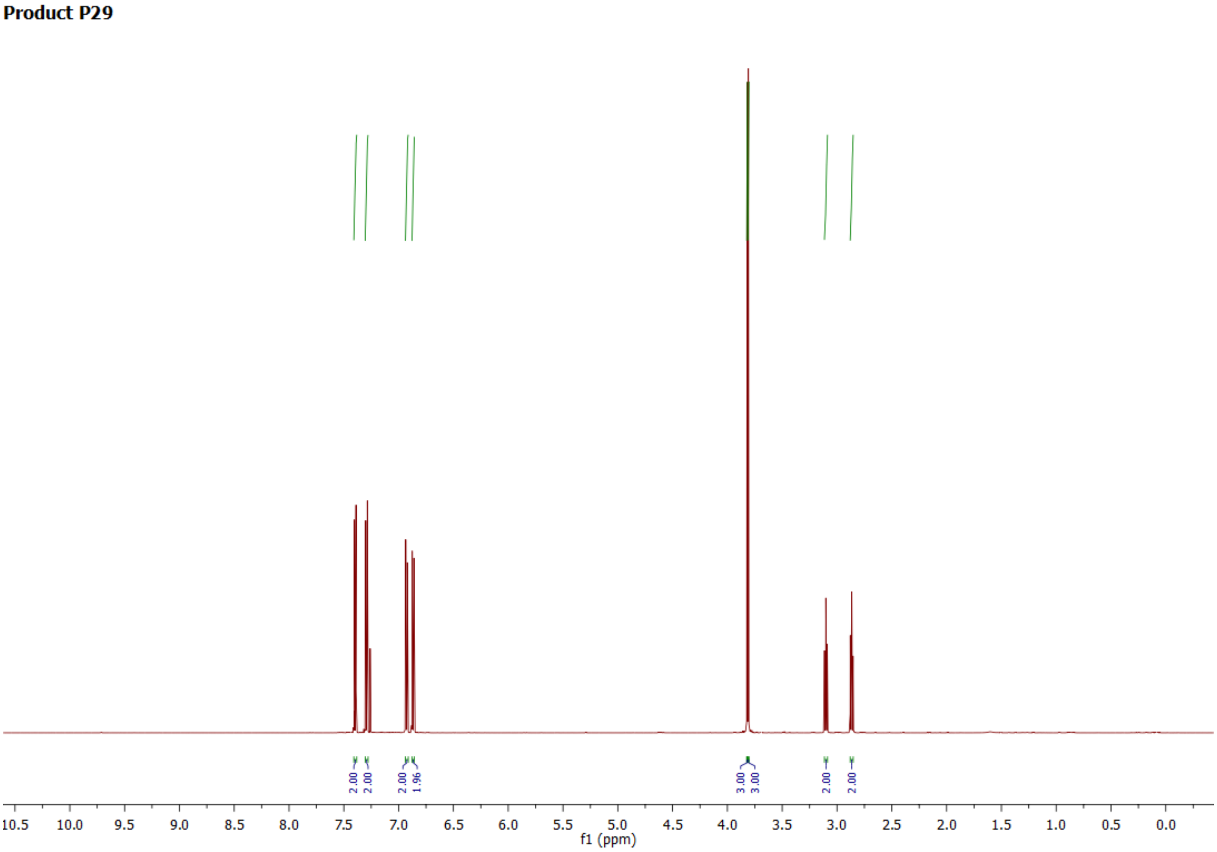

Figure S66. ^{1}H NMR (400 MHz, $CDCl_3$) of product **P29**

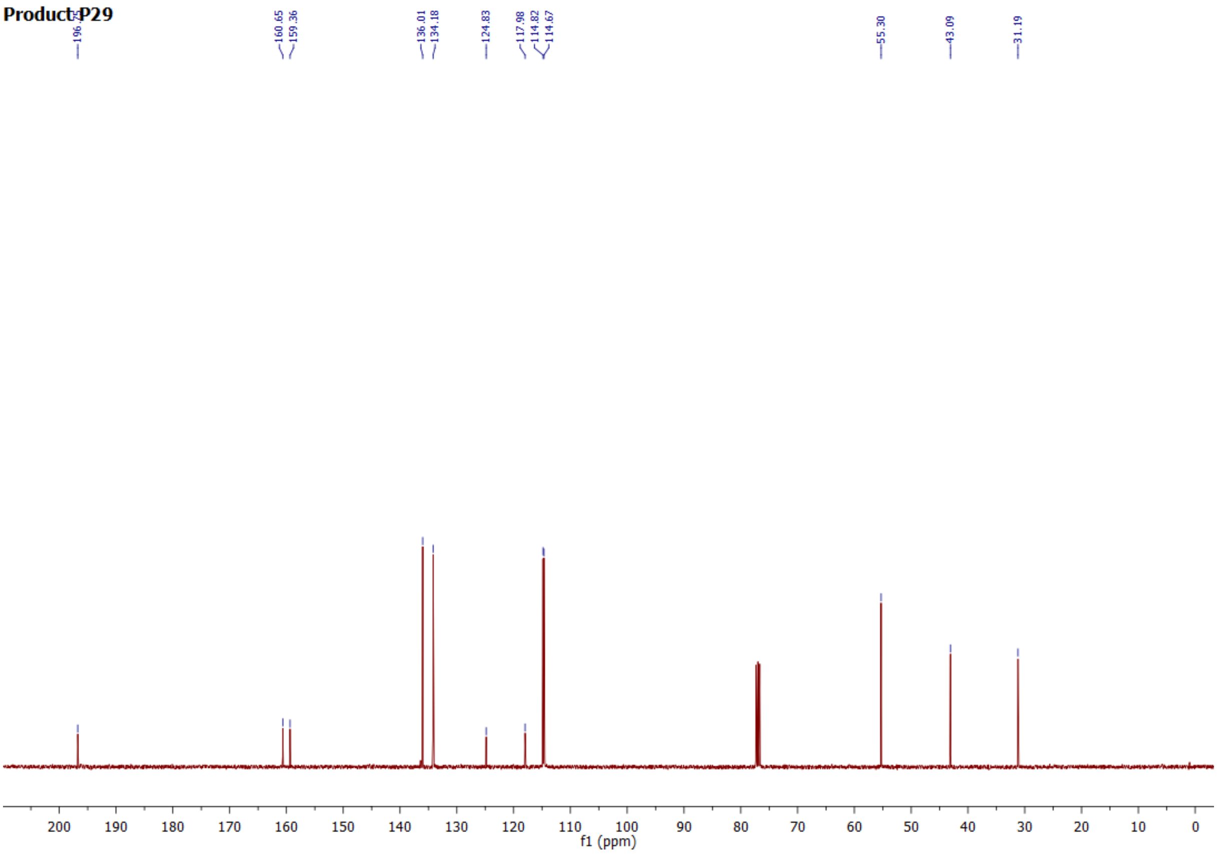

Figure S67. ^{13}C NMR (101 MHz, $CDCl_3$) of product **P29**

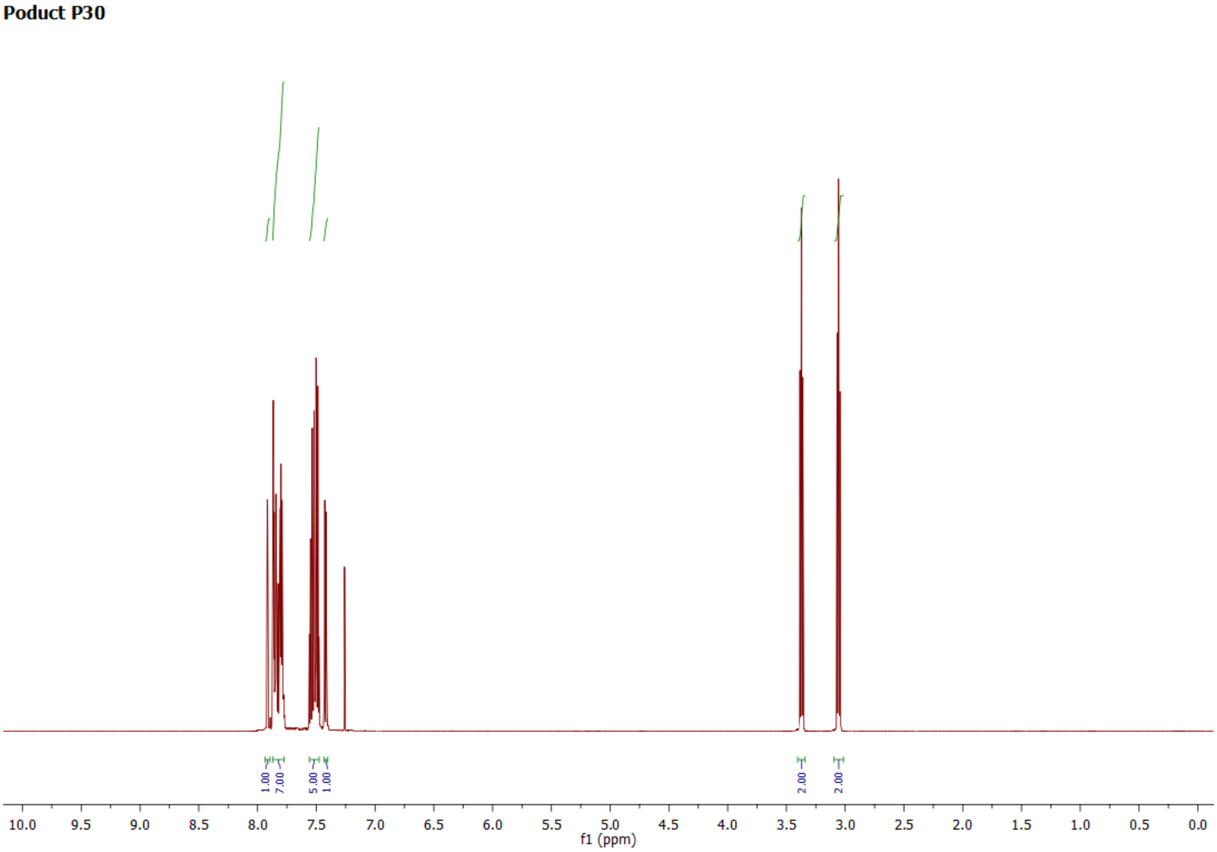

Figure S68. ^{1}H NMR (400 MHz, $CDCl_3$) of product **P30**

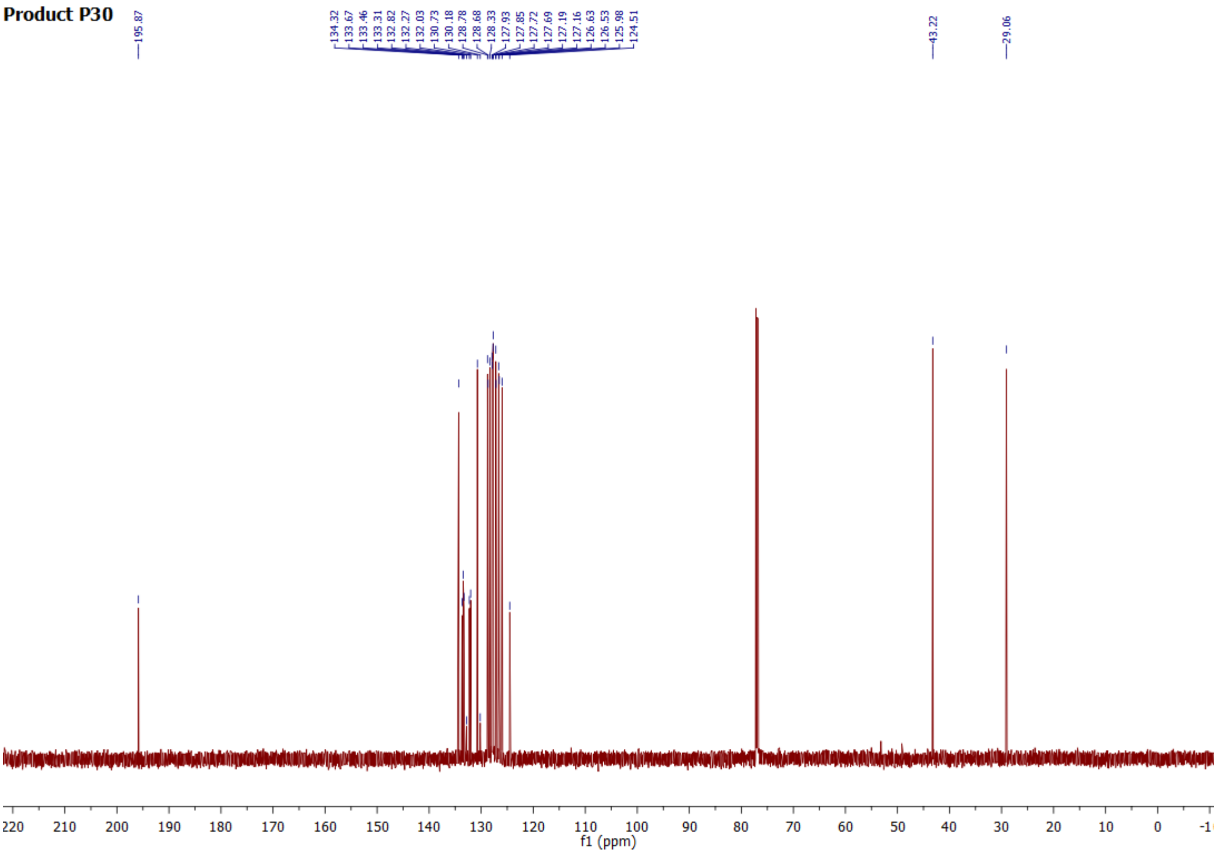

Figure S69. ^{13}C NMR (101 MHz, $CDCl_3$) of product **P30**

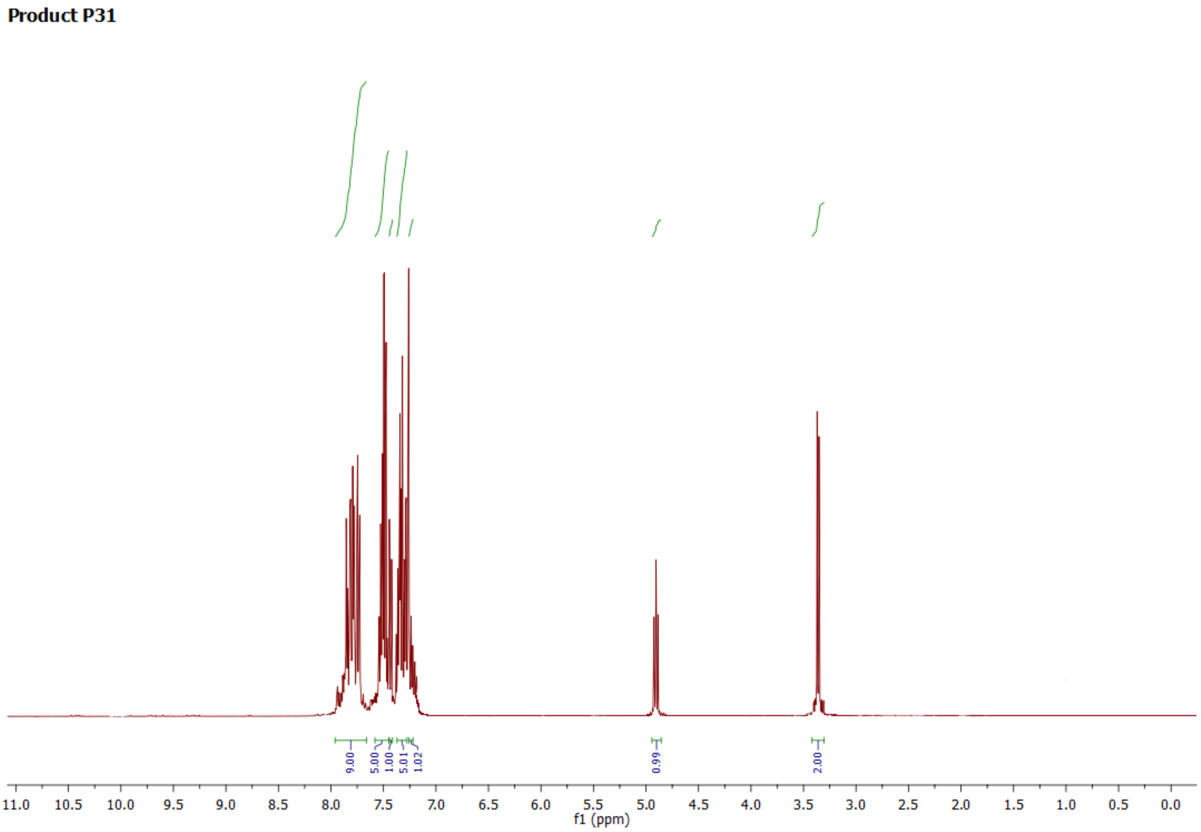

Figure S70. ^{1}H NMR (400 MHz, $CDCl_3$) of product **P31**

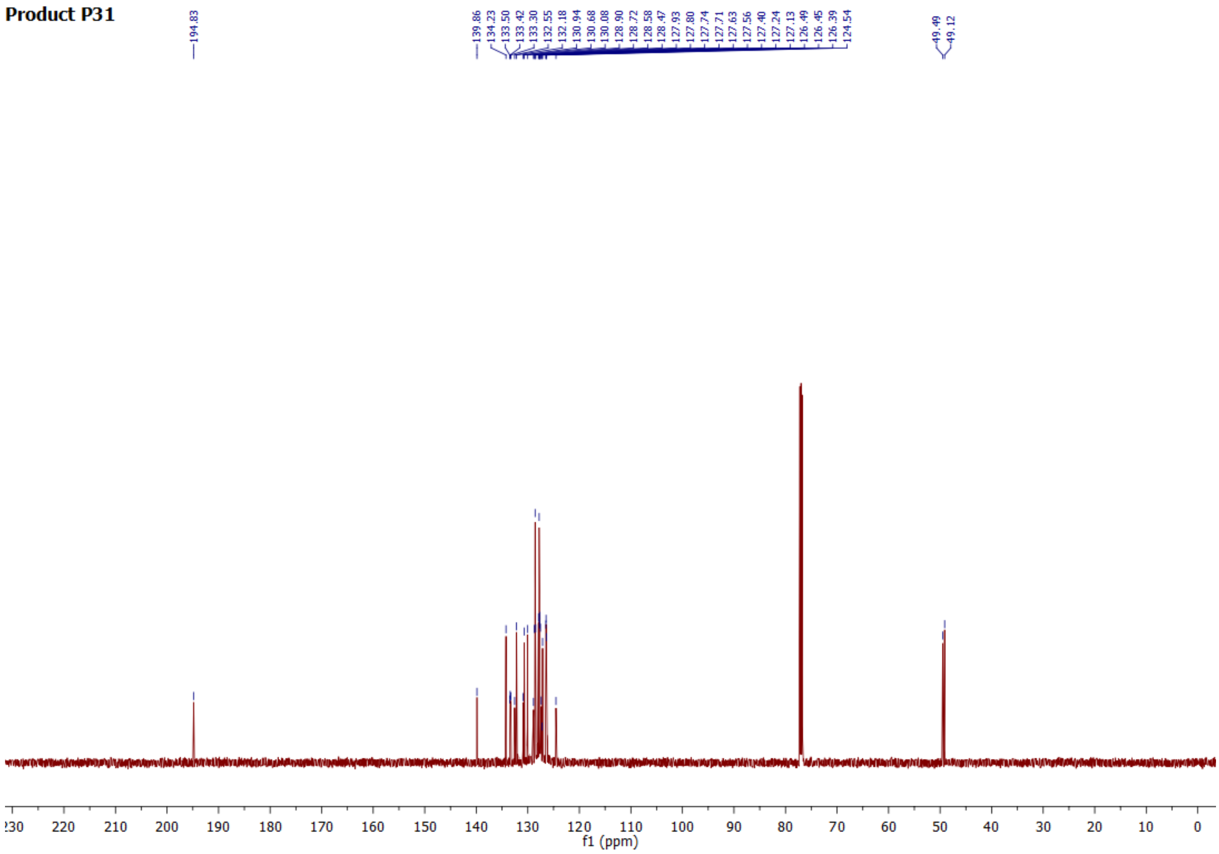

Figure S71. ^{13}C NMR (101 MHz, $CDCl_3$) of product **P31**

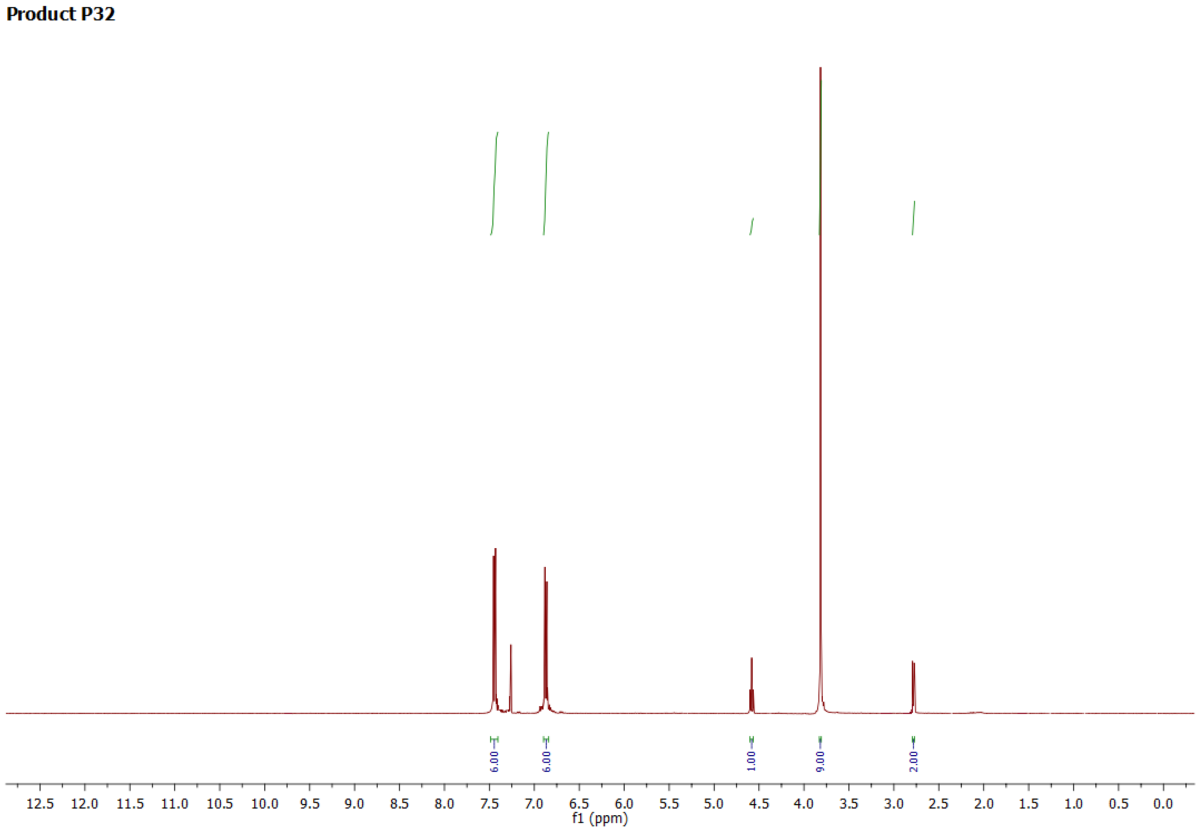

Figure S72. ^{1}H NMR (400 MHz, $CDCl_3$) of product **P32**

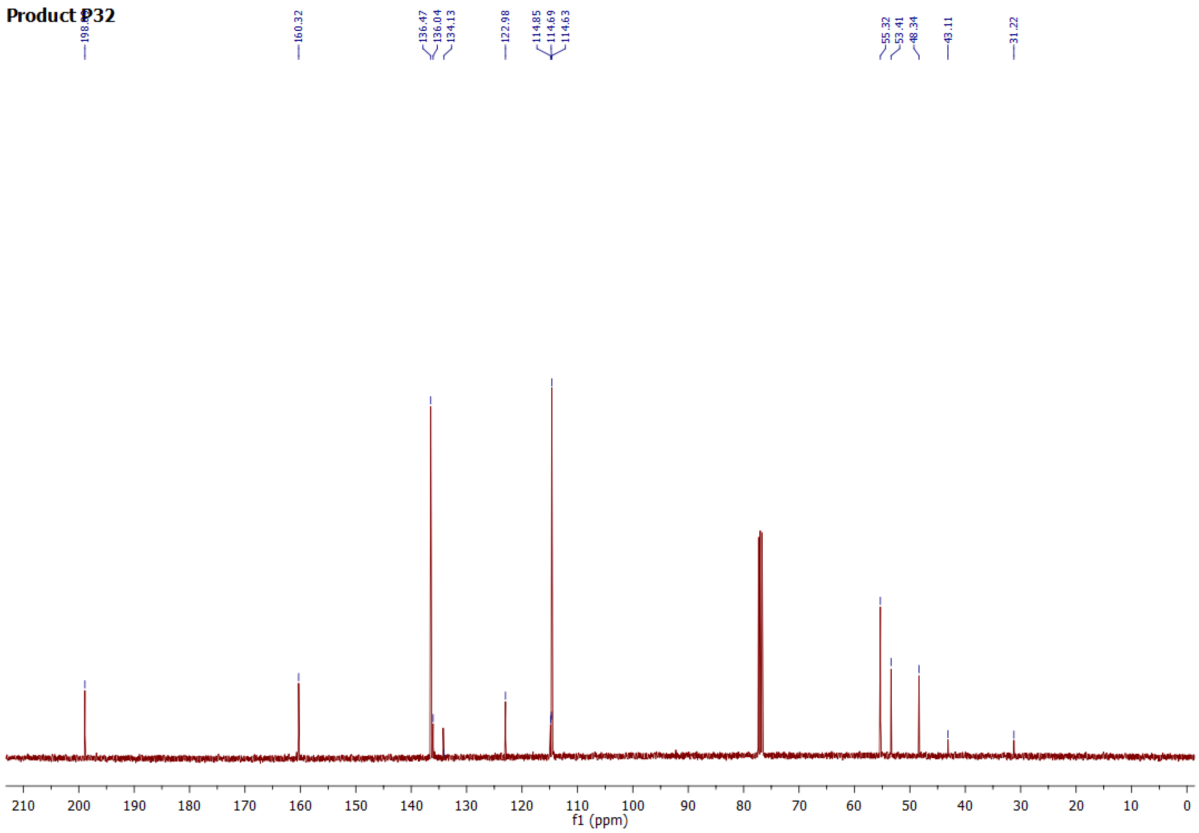

Figure S73. ^{13}C NMR (101 MHz, $CDCl_3$) of product **P32**

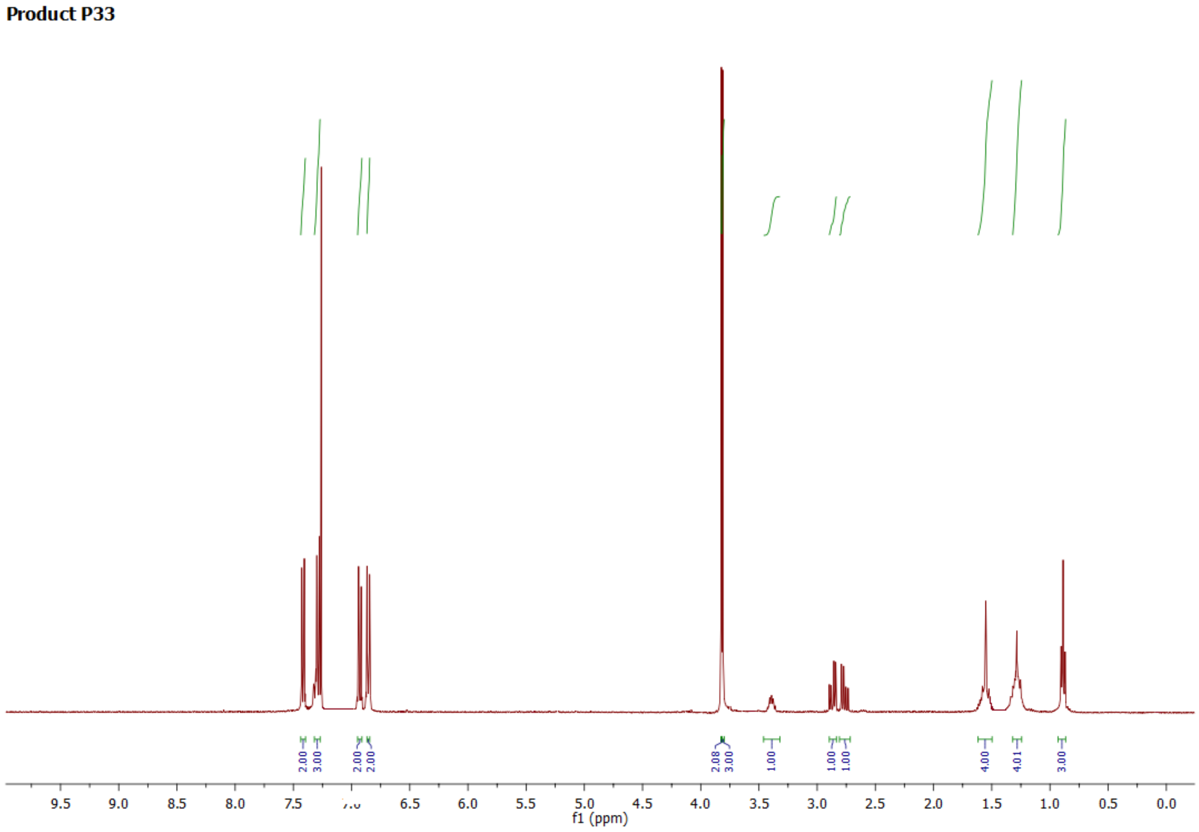

Figure S74. ^{1}H NMR (400 MHz, $CDCl_3$) of product **P33**

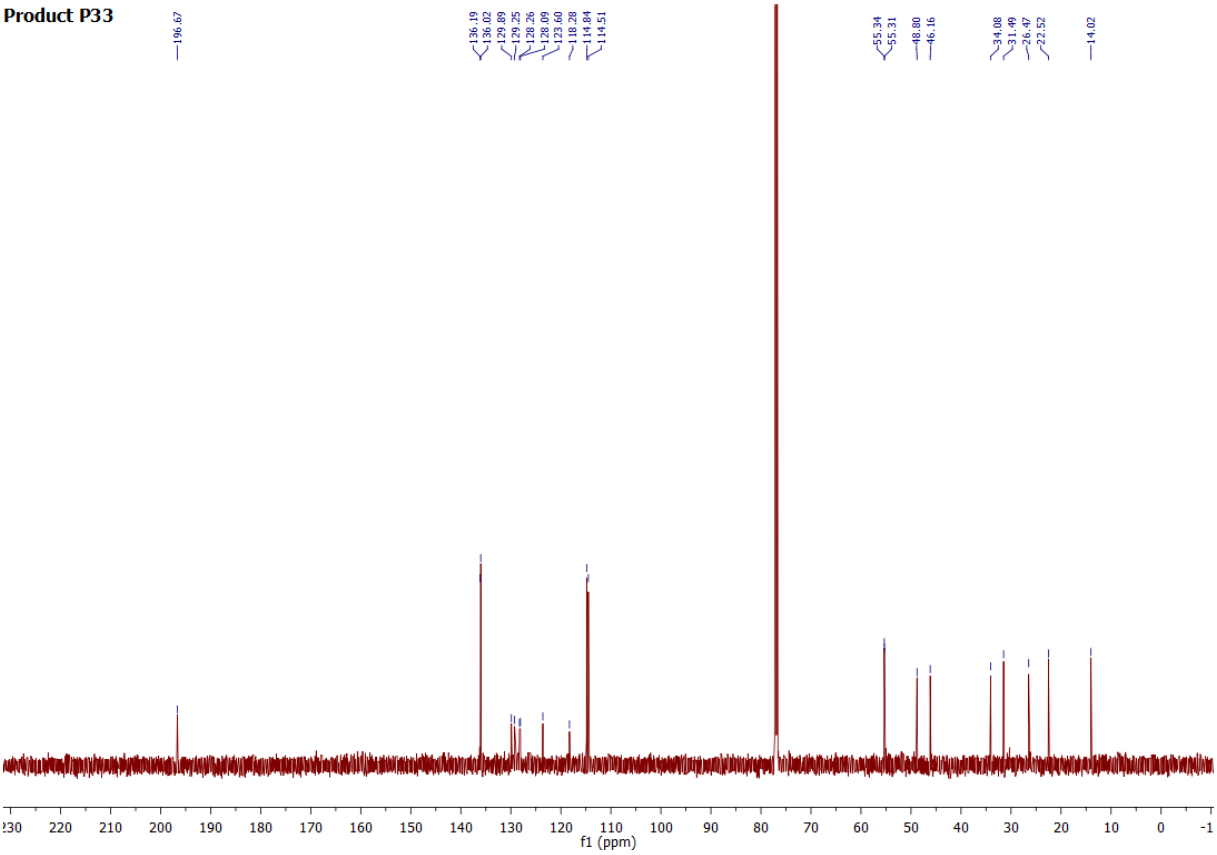

Figure S75. ^{13}C NMR (101 MHz, $CDCl_3$) of product **P33**

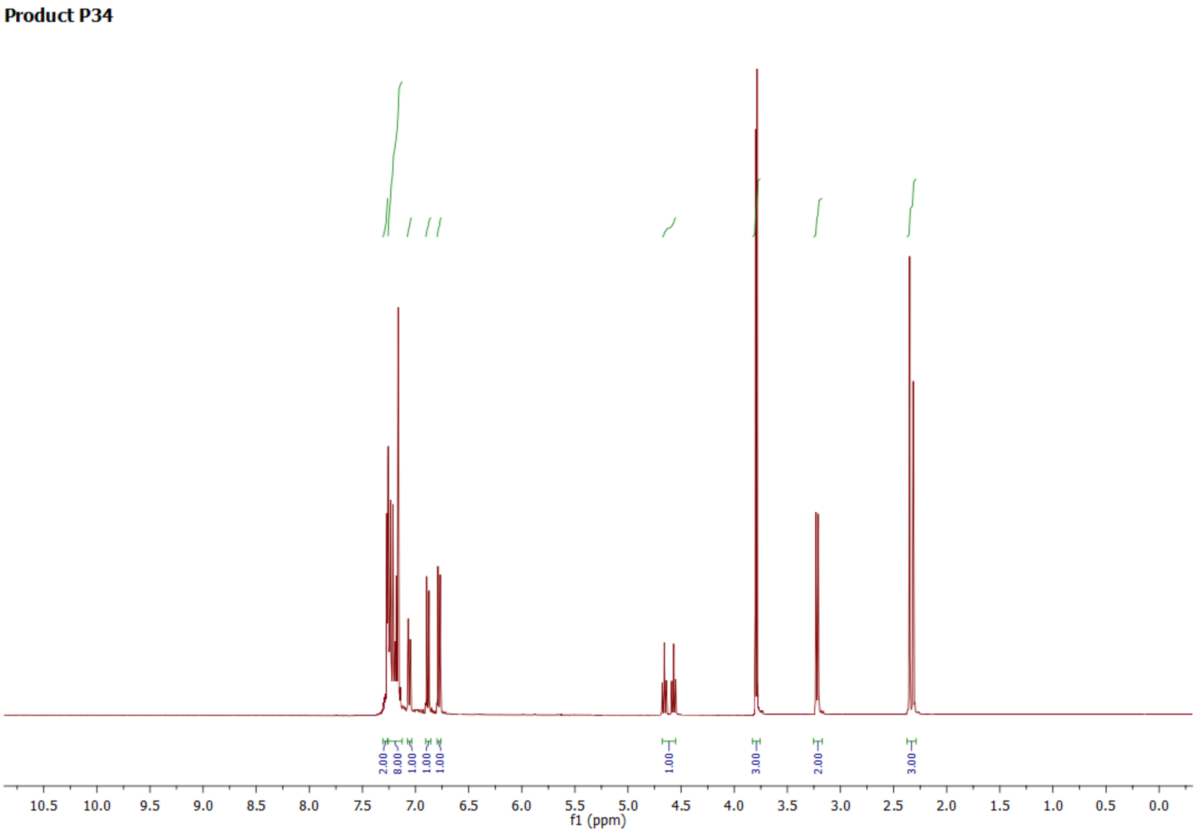

Figure S76. ^{1}H NMR (400 MHz, $CDCl_3$) of product **P34**

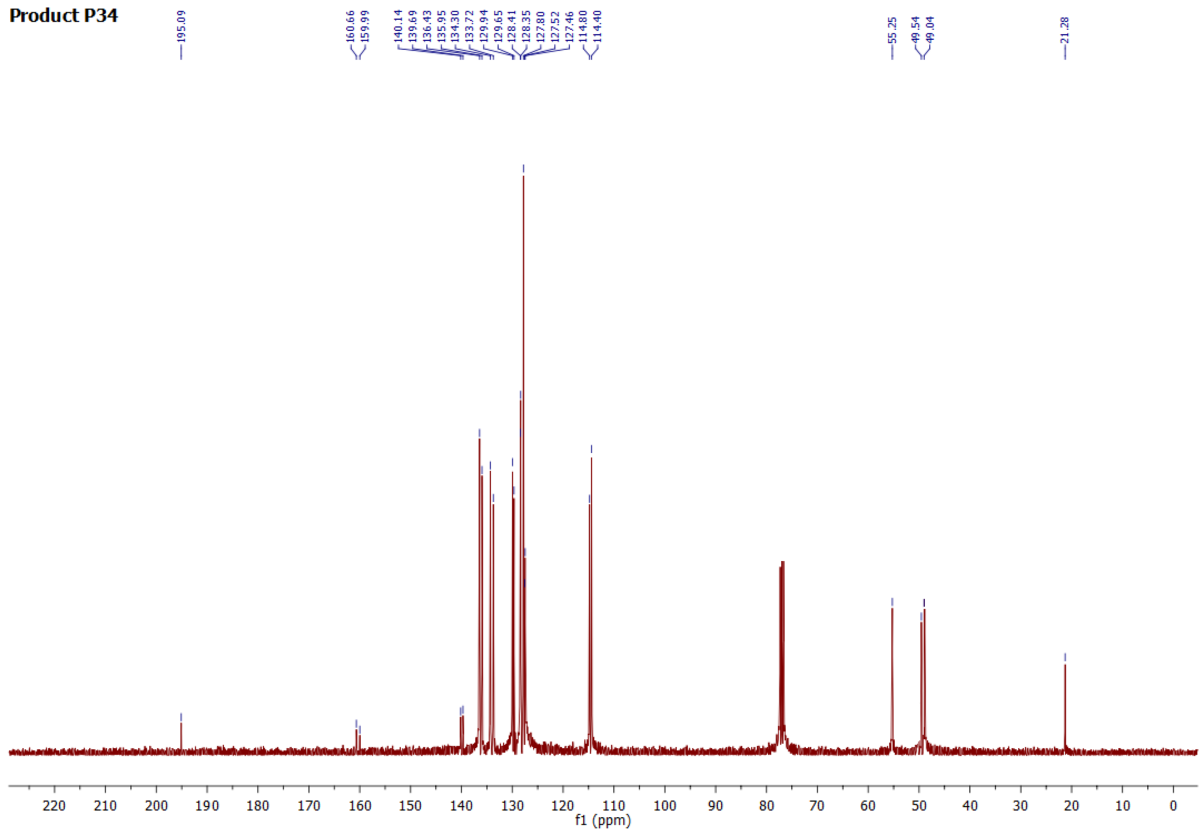

Figure S77. ^{13}C NMR (101 MHz, $CDCl_3$) of product **P34**

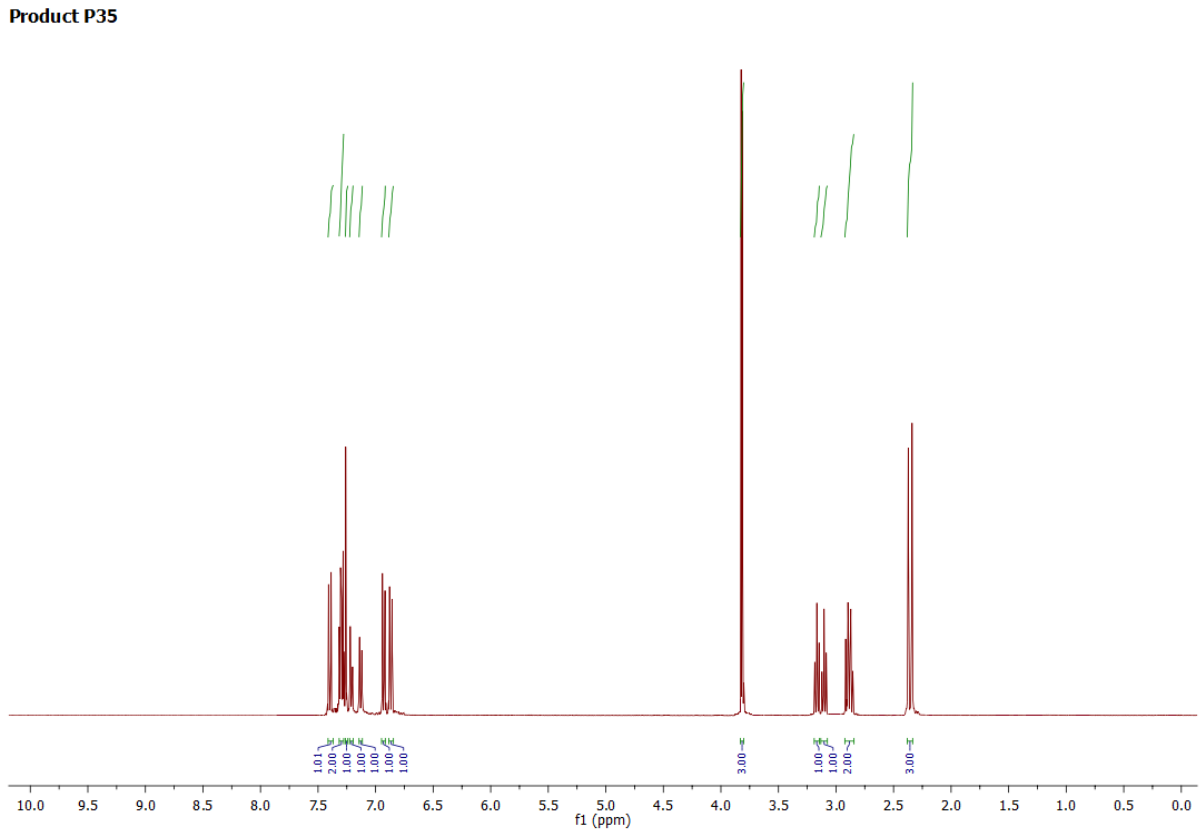

Figure S78. ^{1}H NMR (400 MHz, $CDCl_3$) of product **P35**

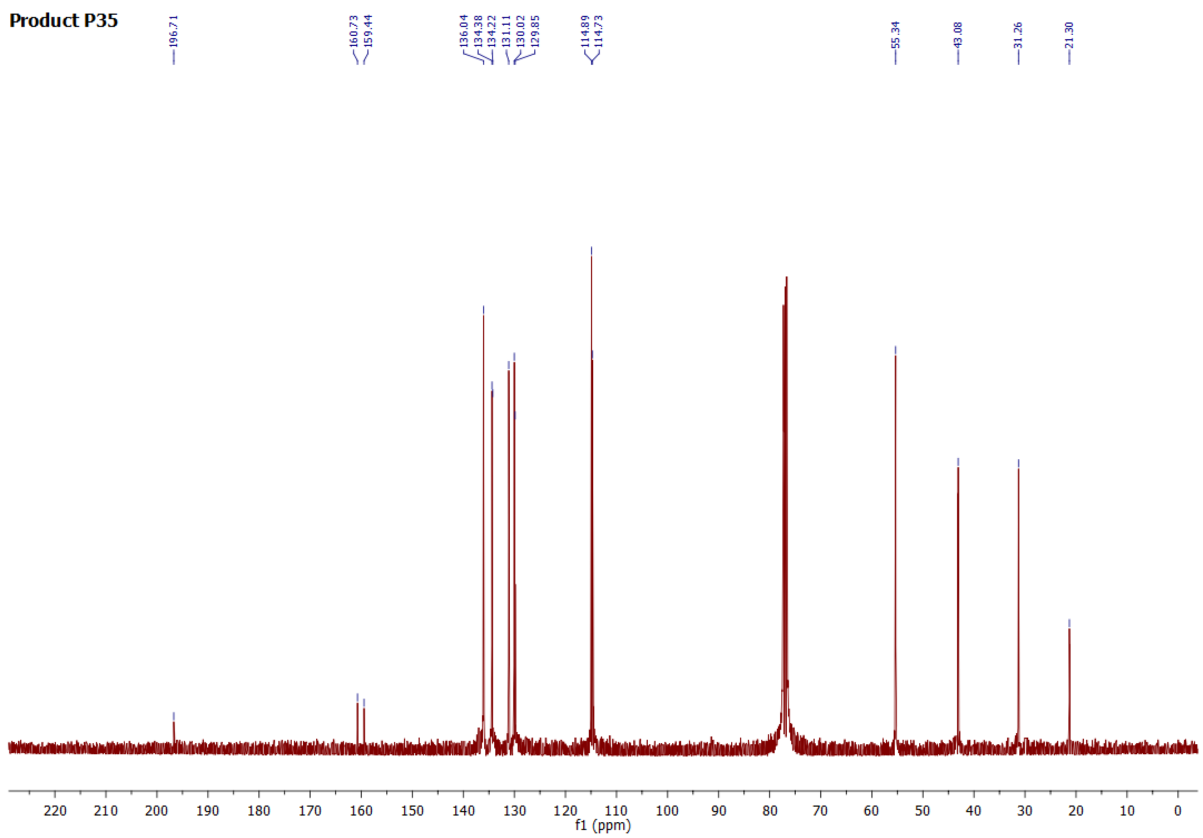

Figure S79. ^{13}C NMR (101 MHz, $CDCl_3$) of product **P35**

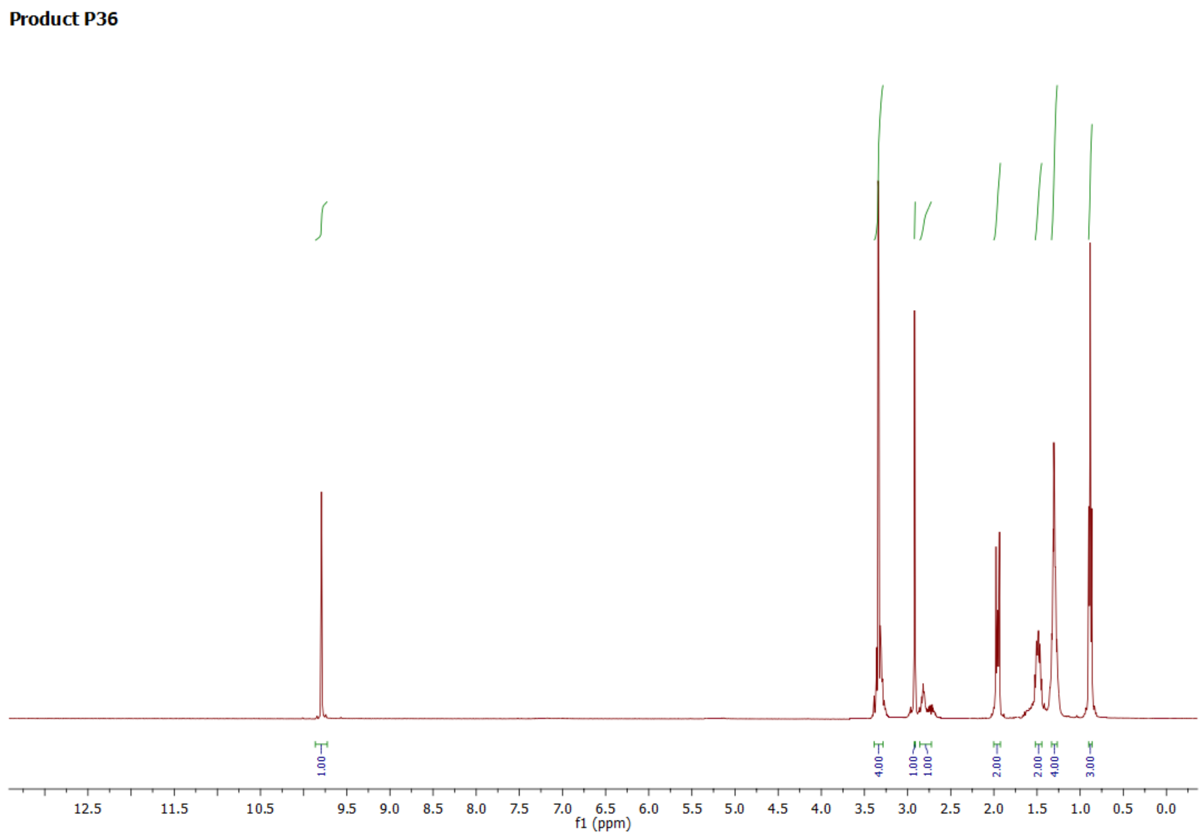

Figure S80. ^{1}H NMR (400 MHz, $CDCl_3$) of product **P36**

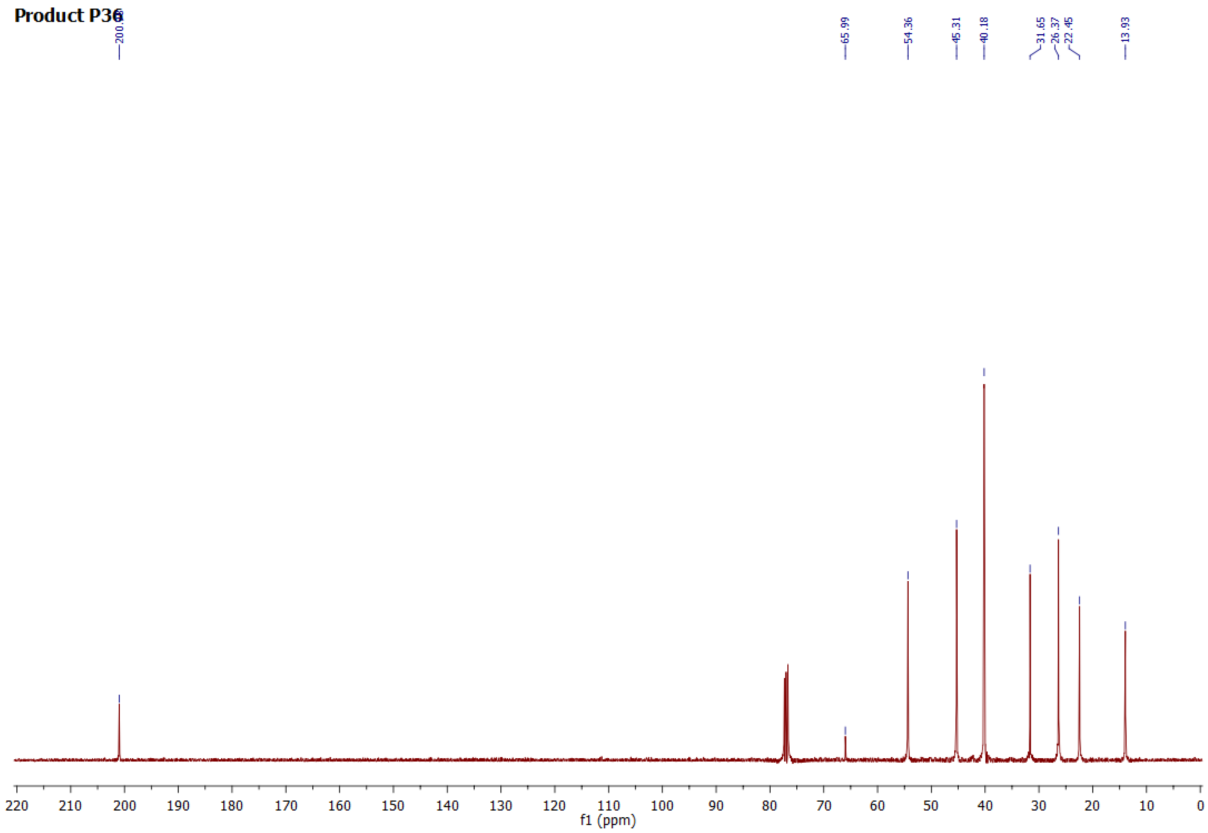

Figure S81. ^{13}C NMR (101 MHz, $CDCl_3$) of product **P36**

3. XRD analyses

Diffraction data were collected by the ω-scan technique on Rigaku SuperNova four-circle diffractometer (Atlas detector) with mirror-monochromatized CuKα radiation (λ = 1.54178 Å) for compounds **P9** and **P14** at room temperature, **P22** and **P24** at 130K. The data were corrected for Lorentz-polarization as well as for absorption effects.[S2] The structures were solved with SHELXT[S3] and refined with the full-matrix least-squares procedure on F^2 by SHELXL-2015[S4] using Olex2 software.[S5] All non-hydrogen atoms were refined anisotropically, hydrogen atoms were placed in idealized positions and refined as 'riding model' with isotropic displacement parameters set at 1.2/1.5 times U_{eq} of appropriate carrier atoms.

Table S1 lists the relevant experimental data and refinement details. Crystallographic data for the structural analysis has been deposited with the Cambridge Crystallographic Data Centre, Nos CCDC 2339336-2339339. Copies of this information may be obtained free of charge from: The Director, CCDC, 12 Union Road, Cambridge, CB2 1EZ, UK. Fax: +44(1223)336-033, e-mail:deposit@ccdc.cam.ac.uk or www: http://www.ccdc.cam.ac.uk

Table S1. Crystal data, data collection and structure refinement

Compound	P9	P44	P22	P24
Empirical formula	$C_{10}H_{10}O_2S$	$C_{16}H_{14}O_2S$	$C_{21}H_{16}F_2OS_2$	$C_{23}H_{22}O_3S_2$
Formula weight	194.24	270.33	386.46	410.52
Temperature/K	293(2)	293(2)	130.00(14)	130.00(10)
Crystal system	monoclinic	monoclinic	monoclinic	monoclinic
Space group	$P2_1/c$	$P2_1/n$	$P2_1/n$	$P2_1/c$
a/Å	14.1829(2)	8.8401(2)	16.9020(3)	22.37466(13)
b/Å	5.57480(10)	17.8685(4)	5.45573(9)	5.53864(3)
c/Å	13.1896(2)	9.0806(2)	19.8038(4)	16.42388(10)
α/°	90	90	90	90
β/°	103.4400(10)	105.159(2)	94.0782(17)	92.4939(5)
γ/°	90	90	90	90
Volume/Å^3	1014.30(3)	1384.45(6)	1821.54(6)	2033.41(2)
Z	4	4	4	4
ρ_{calc}g/cm^3	1.272	1.297	1.409	1.341
μ/mm^{-1}	2.557	2.030	2.885	2.544
F(000)	408.0	568.0	800.0	864.0
Crystal size/mm^3	0.4 × 0.3 × 0.3	0.2 × 0.15 × 0.15	0.4 × 0.2 × 0.1	0.3 × 0.3 × 0.05
Radiation	Cu Kα (λ = 1.54184)	Cu Kα (λ = 1.54184)	Cu Kα (λ = 1.54184)	Cu Kα (λ = 1.54184)
2Θ range for data collection/°	6.408 to 147.854	9.9 to 148.134	6.646 to 148.634	7.91 to 149.064
Index ranges	$-17 \le h \le 14$, $-6 \le k \le 4$, $-16 \le l \le 16$	$-10 \le h \le 10$, $-22 \le k \le 20$, $-10 \le l \le 11$	$-20 \le h \le 20$, $-6 \le k \le 5$, $-19 \le l \le 24$	$-27 \le h \le 27$, $-6 \le k \le 6$, $-20 \le l \le 20$
Reflections collected	5685	5815	7367	20623
Independent reflections	1980 [R_{int} = 0.0140, R_{sigma} = 0.0120]	2737 [R_{int} = 0.0176, R_{sigma} = 0.0213]	3628 [R_{int} = 0.0267, R_{sigma} = 0.0327]	4107 [R_{int} = 0.0195, R_{sigma} = 0.0125]
Data/restraints/parameters	1980/0/119	2737/0/173	3628/0/235	4107/0/255
Goodness-of-fit on F^2	1.069	1.041	1.032	1.053
Final R indexes [I>=2σ (I)]	R_1 = 0.0422, wR_2 = 0.1261	R_1 = 0.0331, wR_2 = 0.0891	R_1 = 0.0330, wR_2 = 0.0826	R_1 = 0.0544, wR_2 = 0.1367
Final R indexes [all data]	R_1 = 0.0444, wR_2 = 0.1295	R_1 = 0.0411, wR_2 = 0.0958	R_1 = 0.0431, wR_2 = 0.0891	R_1 = 0.0563, wR_2 = 0.1384
Largest diff. peak/hole / e Å^{-3}	0.19/-0.24	0.19/-0.16	0.27/-0.24	1.54/-0.74

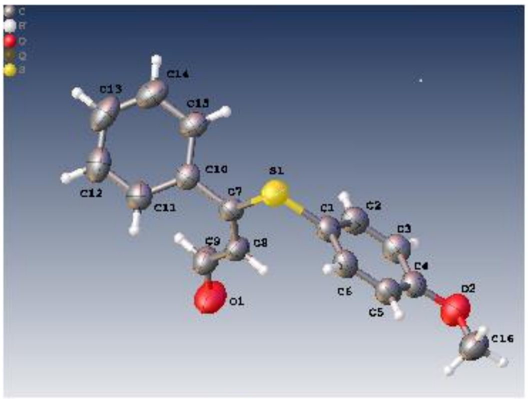

P9

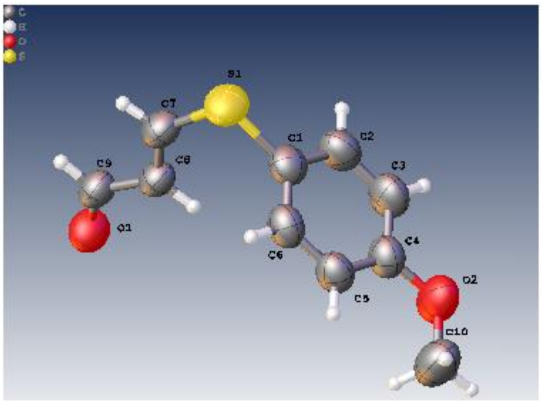

P14

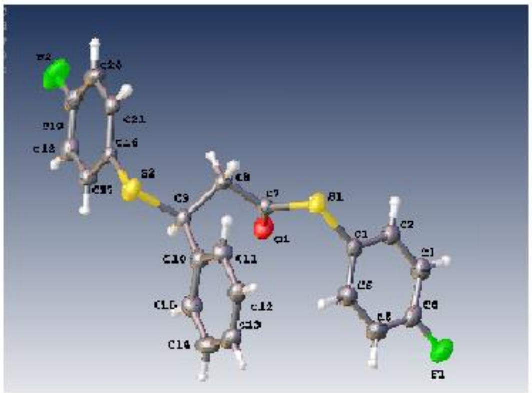

P22

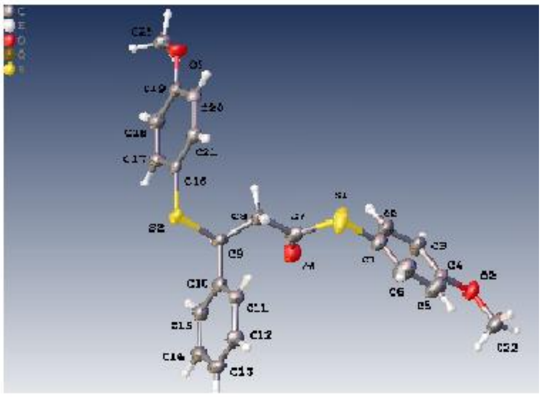

P24

Figure S82. A perspective views of the molecules **P9, P14, P22 and P24**

4. References

[S1] M. Kardela, K. Halikowska-Tarasek, M. Szostak, E. Bisz, *Catal. Sci. Technol.*, 2022, 12, 7275-7280.

[S2] CrysAlisPro 1.171.42.56a (Rigaku Oxford Diffraction, 2022).

[S3] G. M. Sheldrick, *Acta Crystallogr. Sect. A Found. Crystallogr.*, 2015, 71, 3–8.

[S4] G. M. Sheldrick, *Acta Crystallogr. Sect. C Struct. Chem.*, 2015, 71, 3–8.

[S5] O.V. Dolomanov, L.J. Bourhis, R.J. Gildea, J.A.K. Howard, H. Puschmann, J. *Appl. Crystallogr*., 42 (2009), pp. 339-341,
